# Predicted class-I aminoacyl tRNA synthetase-like proteins in non-ribosomal peptide synthesis

**DOI:** 10.1186/1745-6150-5-48

**Published:** 2010-08-02

**Authors:** L Aravind, Robson F de Souza, Lakshminarayan M Iyer

**Affiliations:** 1 National Center for Biotechnology Information, National Library of Medicine, National Institutes of Health, Bethesda, MD 20894, USA

## Abstract

**Background:**

Recent studies point to a great diversity of non-ribosomal peptide synthesis systems with major roles in amino acid and co-factor biosynthesis, secondary metabolism, and post-translational modifications of proteins by peptide tags. The least studied of these systems are those utilizing tRNAs or aminoacyl-tRNA synthetases (AAtRS) in non-ribosomal peptide ligation.

**Results:**

Here we describe novel examples of AAtRS related proteins that are likely to be involved in the synthesis of widely distributed peptide-derived metabolites. Using sensitive sequence profile methods we show that the cyclodipeptide synthases (CDPSs) are members of the HUP class of Rossmannoid domains and are likely to be highly derived versions of the class-I AAtRS catalytic domains. We also identify the first eukaryotic CDPSs in fungi and in animals; they might be involved in immune response in the latter organisms. We also identify a paralogous version of the methionyl-tRNA synthetase, which is widespread in bacteria, and present evidence using contextual information that it might function independently of protein synthesis as a peptide ligase in the formation of a peptide- derived secondary metabolite. This metabolite is likely to be heavily modified through multiple reactions catalyzed by a metal-binding cupin domain and a lysine N6 monooxygenase that are strictly associated with this paralogous methionyl-tRNA synthetase (MtRS). We further identify an analogous system wherein the MtRS has been replaced by more typical peptide ligases with the ATP-grasp or modular condensation-domains.

**Conclusions:**

The prevalence of these predicted biosynthetic pathways in phylogenetically distant, pathogenic or symbiotic bacteria suggests that metabolites synthesized by them might participate in interactions with the host. More generally, these findings point to a complete spectrum of recruitment of AAtRS to various non-ribosomal biosynthetic pathways, ranging from the conventional AAtRS, through closely related paralogous AAtRS dedicated to certain pathways, to highly derived versions of the class-I AAtRS catalytic domain like the CDPSs. Both the conventional AAtRS and their closely related paralogs often provide aminoacylated tRNAs for peptide ligations by MprF/Fem/MurM-type acetyltransferase fold ligases in the synthesis of peptidoglycan, N-end rule modifications of proteins, lipid aminoacylation or biosynthesis of antibiotics, such as valinamycin. Alternatively they might supply aminoacylated tRNAs for other biosynthetic pathways like that for tetrapyrrole or directly function as peptide ligases as in the case of mycothiol and those identified here.

**Reviewers:**

This article was reviewed by Andrei Osterman and Igor Zhulin.

## Findings

In addition to their role as basic players in protein synthesis, both tRNAs and AAtRS participate in non-ribosomal peptide synthesis [1]. These molecules, along with peptide ligases, are key players in synthesis of the oligopeptide chains of the Gram-positive type bacterial cell-wall [2,3]. In peptidoglycan biogenesis, the N-acetyl-muramyl-linked pentapeptide stem is synthesized by Mur ligases of the P-loop kinase fold [4]. The terminal D-Ala-D-Ala or D-Ala-D-lactate dipeptide of the stem is synthesized by peptide ligases of the ATP-grasp fold [5]. ATP-grasp peptide ligases are also required for synthesis of D-amino acid-containing cross-links of the Gram-positive-type cell-walls, such as D-Asp, D-Glu and D-Ala-D-Ser [6]. In contrast, glycine- and L-amino acid-containing cross-links are synthesized by the Fem ligases of the GCN5-like acetyltransferase (GNAT) fold. These enzymes utilize aminoacylated tRNAs as substrates for peptide ligation, with aminoacylation of tRNAs being catalyzed by the cognate AAtRSs [2,3]. Also related to the Fem ligases are the protein argininyl and phenylalanyl/leucyl transferases that utilize aminoacylated R-tRNA and F-/L-tRNAs to transfer these amino acids to the N-termini of proteins [5]. Ligation of these amino acids alters the N-termini of proteins and thereby affects their stability based on the N-end rule [1]. Similarly, tRNAs charged with the cognate amino acids by lysyl- or alanyl-tRNA synthetases are used by bacterial enzymes typified by *Clostridium perfringens *MprF in the aminoacylation of membrane phosphatidylglycerol [7]. This neutralizes the charge of the membrane and consequently makes it impermeable to antibacterial peptides. We recently observed that in several bacteria, like actinomycetes, the MprF-like proteins are fused to a paralogous version of the lysyl-tRNA synthetase that is likely to function exclusively in the context of lipid aminoacylation [5]. Likewise, another MprF-like enzyme from *Streptomyces viridifaciens*, VlmA, functions with a distinct seryl-tRNA synthetase paralog, VlmL in transferring serine to isobutylhydroxylamine in biosynthesis of valinamycin [8]. Our recent analysis of peptide ligases showed that MprF/VlmA-like enzymes are members of the GNAT fold and specifically related to the Fem ligases and R and F/L transferases [5]. Thus, this family of GNAT fold enzymes has evolved to function in conjunction with tRNAs, and regular or specific paralogous AAtRSs, charging the cognate amino acids in synthesis of amino acid derived antibiotics or in modifying lipids and proteins.

Recently two peptide bond-forming systems that depend on tRNAs or AAtRSs, but unrelated to the GNAT fold enzymes, have been characterized. One of these is the AlbC family of CDPSs involved in the synthesis of cyclic dipeptide (CDP) secondary metabolites, such as the *Streptomyces noursei *antibiotic albonoursin, a cyclo(L-Phe-L-Leu) derivative, the *Bacillus subtilis *siderophore pulcherriminic acid, a cyclo(L-Leu-L-Leu) derivative and a possible *Mycobacterium tuberculosis *siderophore, which is a cyclo(L-Tyr-L-Tyr) derivative [9]. These CDPSs use aminoacylated tRNAs as their substrates to catalyze the formation of a given cyclic dipeptide. Another distinct amide linkage is the amino-sugar cysteine linkage seen in mycothiol, a low molecular weight reductant seen in several bacteria such as *Mycobacterium *[10]. Formation of this linkage depends on a member of the class-I AAtRS superfamily, MshC which is a cysteinyl-tRS (CtRS) paralog that apparently functions independently of tRNA. It is believed to first adenylate the COOH group of cysteine followed by the transfer of the cysteinyl group to the NH_2 _group of glucosamine, thus resulting in a sugar-amino acid conjugate. These discoveries point to a remarkable variety of non-ribosomal peptide synthesis systems that depend on components recruited from the conventional protein synthesis apparatus. However, the full diversity of these systems and their catalytic possibilities remain to be explored.

Recently, we undertook a systematic analysis of peptide ligases and observed that majority of peptide ligases could be unified into a small set of folds [5]. However, the provenance of the CDPSs remains unclear as previous studies have failed to detect significant similarity to any of these known peptide ligase domains [9]. Secondly, the extent to which AAtRSs might function as peptide ligases remains unclear - is MshC a one-off example or are there more such instances? We used sensitive sequence analysis and comparative genomics techniques to investigate these questions. Consequently we found that the CDPSs belong to the HUP clade of Rossmannoid folds and are likely to have been derived from a class-I AAtRS-like precursor. We also present evidence that there are other instances wherein class-I AAtRSs participate in the synthesis of non-ribosomal peptide metabolites, suggesting that their role in such processes is a more general one.

## Results and discussion

### Sequence analysis of the CDPSs shows that it is a HUP clade Rossmannoid domain related to the class-I AAtRS catalytic domain

**Figure 1:**
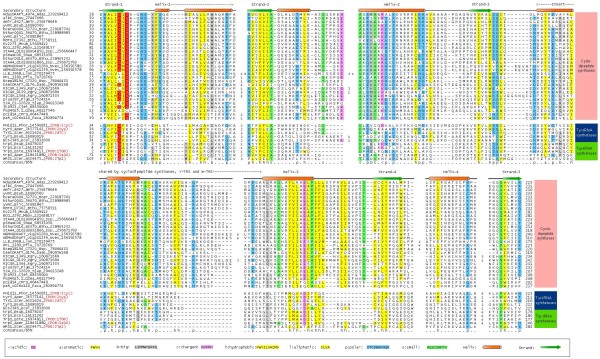
**Alignment of the CDPS and class-I AAtRS catalytic domains**. Sequences are labeled by their gene names, species abbreviations and Genbank index numbers separated by underscores. PDB ids, if available, are also shown. Sequences are colored based on 85% consensus derived from an alignment of the cyclopeptide ligases. A key for the coloring scheme, consensus abbreviations and secondary structure labels is shown in the box below the alignment. Familial affiliations of the sequences are shown to the right. Species names are expanded in Abbreviations.

To understand the origins of the CDPSs we initiated iterative PSI-BLAST searches [11] with different representatives of the family such as AlbC from *S. noursei *[9]. In addition to the previously characterized representatives from firmicutes, actinobacteria and *Photorhabdus*, we also recovered several divergent versions (e < 10^-3 ^at the time of first detection) from other bacteria such as *Parachlamydia, Pseudomonas fluorescens, Legionella, Sphingobium *and *Rickettsiella grylli *prior to convergence (For details on Material and Methods refer to Additional file 1). These searches also detected homologous proteins in eukaryotes, such as the fungus *Gibberella*, the annelid worm *Platynereis *and the sea anemone *Nematostella*. All these versions were standalone proteins with no fusions to any other domains. A multiple alignment of the recovered representatives followed by secondary structure prediction with the JPRED program [12] revealed an α/β fold with five strand-helix units comprising the core of the fold with helical inserts after the second strand-helix unit and after the third strand (Figure 1). The sequence conservation pattern revealed a GxSxxp (where p is a polar residue, usually an asparagine) between the first strand and helix (Figure 1). A further conserved polar residue was found at the C-terminus of the 3^rd ^predicted strand and a conserved glutamate in the helical region between strand-3 and strand-4 (Figure 1). These are predicted to comprise the active site of the CDPSs. The presence of five strand-helix units along with an active site loop after the first strand and a possible active site residue after the 3rd strand is reminiscent of the Rossmannoid domains and suggested that the CDPSs could adopt such a fold [13].

To test this conjecture we used a Hidden Markov Model (HMM) derived from the multiple alignment of the CDPSs in a profile-profile comparison against a library of HMMs generated from all known domains with structural representatives in PDB using the HHpred program [14]. This search recovered catalytic domains of the class-I AAtRSs, namely tyrosyl-tRS (PDB: 2cyc; p = 7 × 10^-6^) and tryptophanyl-tRS (PDB: 3foc; p = 4 × 10^-5^) and the HIGH-motif nucleotidyltransferase, phosphopantetheine adenylyltransferase (1od6; p = 3 × 10^-4^) as the best hits. The class-I AAtRSs and HIGH-motif NTases belong to the HUP (HIGH, UspA, Photolyase/PP-loop) superclass of Rossmannoid domains that, just as predicted for the CDPSs, contain a core sheet with 5 strands [13]. In all members of the HUP superclass the substrate-binding site is found in the loop between strand-1 and helix-1, consistent with the conservation pattern observed in the CDPSs. Indeed, profile-profile matches align the above-mentioned predicted active site loop of the CDPSs with the corresponding loop of the class-I aatRSs and HIGH NTases (Figure 1). Furthermore, most class-I AAtRSs contain a major insert, typically helical, between strand-3 of the core Rossmannoid fold and the helix prior to strand-4 that form a "cap" over the active site [13,15]. This is also the point of insertion of the helical insert observed in the CDPSs (Figure 1). Hence, this insert might form a cap over the core substrate-binding site in the CDPSs. Together these observations indicate that the CDPSs are novel members of the HUP superclass of Rossmannoid domains. However, given their restricted distribution in a relatively small set of bacteria and eukaryotes it is likely that they were derived later in evolution from a class-I AAtRS precursor like the YtRS or the WtRS (Figure 1). This would also explain how the CDPSs could use aminoacyl tRNAs (AAtRNAs) as substrates in peptide ligation--they are predicted to bind them at the active site, similarly to the class-I AAtRSs. However, in the CDPSs the HIGH motif was lost and a novel signature with a conserved serine was acquired, reminiscent of another superfamily of HUP clade, namely the PP-loop ATPases [13] (Figure 1). These changes are likely to be essential features of the CDPSs required to form the amide linkage from AAtRNAs, as opposed to the adenylation followed by ester formation seen in ancestral AAtRS.

**Figure 2:**
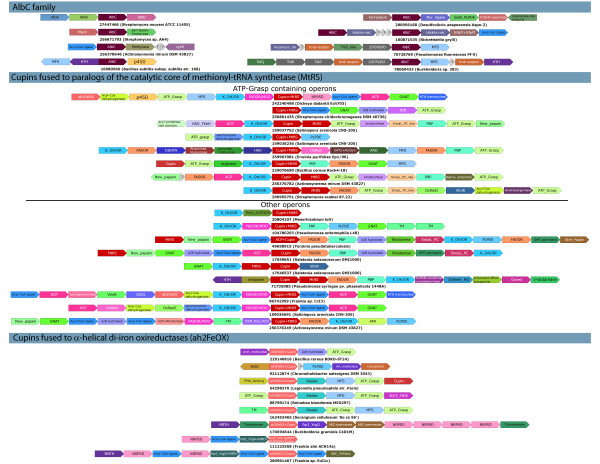
**Examples of predicted operons of novel peptide biosynthetic systems**. Genes are shown as arrows pointing from the 5' to the 3' end of the coding frame. Operons are labeled with the gi and species name of the primary AlbC, MtRS or cupin genes in that context. Gene identifiers are derived from the genome annotation provided by NCBI. Other than the standard domain names the remaining identifiers are provided in Abbreviations. In the AlbC operon AlbA encodes a nitroreductase family enzyme and AlbD a transmembrane protein.

**Figure 3:**
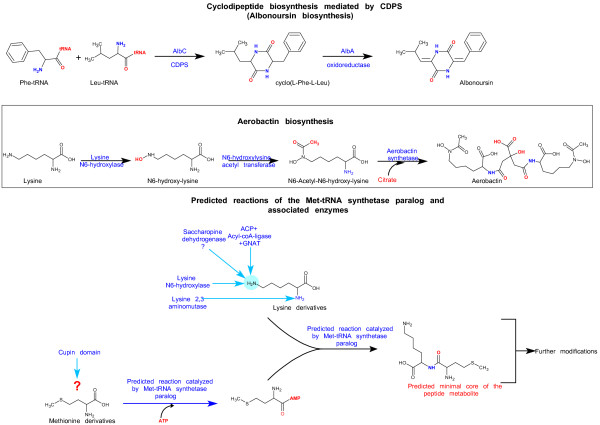
**Simplified scheme showing the core reactions catalyzed by the enzymatic systems described in this work**. The figure shows reaction schemes for the biosynthesis of the cyclopeptide albonoursin, the siderophore aerobactin, and possible substrates of enzymes encoded by the systems based on the MtRS paralogs and reactions they could potentially catalyze.

Previous studies on CDPSs have shown that they are typically encoded in a conserved operon with a gene for an enzyme of the cytochrome P450 family [16] (Figure 2). Studies in *B.subtilis *and *M.tuberculosis *indicate that the cytochrome P450 is required for further oxidative modification of the CDP (Figure 3). In *M.tuberculosis *it catalyzes the cross-linking of the two tyrosine rings of cYY [16]. In synthesis of *B.subtilis *pulcherriminic acid it catalyzes the addition of oxygens to the nitrogens of the diketopiperazin ring of CDP [9]. In contrast, the albonoursin-like operons found in certain actinomycetes links the CDPS with an oxidoreductase of the nitroreductase family (a Rossmann fold dehydrogenase), which is likely to catalyze the α-β desaturation of the two amino acid side chains in the dipeptide (Fig 2, 3). Among the newly detected versions in this study we found several conserved gene neighborhood associations for AlbC-like CDPSs that might be indicative of alternative modifications and synthetic mechanisms for the dipeptides generated by them. For example, in *Burkholderia *sp. 383 and *Pseudomonas fluorescens *we observed novel associations with genes encoding 2-oxoglutarate-dependent dioxygenases that could potentially catalyze hydroxylations of the amino acid side chains of the dipeptide (Figure 2)[17]. Interestingly, in some actinomycetes, like *Actinosynnema mirum *and *Streptomyces *sp. AA4 the AlbC-like CDPS shows a neighborhood association with genes encoding a methyltransferase and an acyl-coA ligase. The former enzyme is related to ubiquinone methylases of the UbiE type and could modify CDPs through methylation. The latter superfamily of enzymes adenylates carboxylate groups and subsequently ligates them to coA via a thiocarboxylate linkage to form an acyl-coA [18]. Hence, these CDPSs could in principle use aminoacyl-coAs generated by the action of the above enzymes as a substrate, rather than AAtRNAs. Indeed, enzymes of the acyl-coA ligase are part of the large condensation domain-dependent non-ribosomal peptide and polyketide synthesis systems [19]. In some cases the CDPS gene co-occurs in predicted operons with an additional peptide ligase (Figure 2) such as a Mur ligase of the P-loop kinase superfamily (e.g. *Desulfovibrio aespoeensis*) or a GNAT fold ligase (e.g. *Rickettsiella grylli*) that could mediate formation of further peptide linkages. These operons might also contain a SelA-like pyridoxal phosphate dependent enzyme (PLPDE) that could synthesize a modified amino acid that is incorporated into the peptide (Figure 2).

Beyond free-living bacteria with active secondary metabolism systems we also found CDPSs in several phylogenetically distant intracellular parasitic bacteria, such as *Legionella, Rickettsiella *and certain chlamydiae (Figure 1). Typically, these bacteria do not encode biosynthetic systems for secondary metabolites that are common in strongly competing slow-growing forms like actinomycetes and myxobacteria. Hence, the spread of such CDPSs across diverse intracellular bacterial pathogens/symbionts suggests a potential role for particular dipeptides in surviving in or manipulating host cells. Detection of a CDPS in the plant pathogenic fungus *Gibberella zeae *is consistent with early reports of the generation of CDPs by such fungi with a bioactive effect on their hosts [20]. Interestingly, the CDPS gene encoded by the annelid *Platynereis *is induced as part of the antibacterial innate immune response [21], suggesting that CDPs might play a role as endogenously encoded antibiotics in the immune response of certain animals.

### Identification of a widespread methionyl-tRNA synthetase paralog that might be involved in novel peptide synthetic pathways

The above finding that the CDPSs are highly derived offshoots of a class-I AAtRS-like domain, together with the identification of the paralogous CtRS as the amino acid ligase in mycothiol biosynthesis [10], indicated that AAtRS homologs could also directly function as peptide ligases. To investigate if other AAtRS could function in such a capacity we hoped to use contextual information from predicted operons and domain fusions to identify potential candidates. Recently we identified a paralogous version of the MtRS in several bacterial genomes that is fused to a double-stranded β-helix domain of the metal-binding cupin class [17]. Further analysis showed that related paralogous MtRSs are encoded by several other bacterial genomes - these lack fusion to the cupin domain, but in all these cases a standalone cupin is encoded by a neighboring gene in the genome (Figure 2). While a given bacterium might have 1-3 copies of this paralogous MtRS, it always also contains the conventional MtRS involved in protein synthesis (Additional file 1). This suggested that the paralogous MtRS is likely to be dedicated to a pathway involved in synthesis of a distinct metabolite, separate from protein synthesis. Given that the catalytic residues required for adenylation of the amino acid are intact in this paralogous family they are predicted to be catalytically active enzymes capable of adenylating methionine (Additional file 1). This also implies that the metabolite synthesized by these enzymes is likely to be a derivative of methionine.

To better understand the provenance of these MtRS paralogs, we included them in a phylogenetic analysis along with the conventional MtRS. The resultant tree showed that the conventional MtRSs fall in two major clusters separated by a long internal branch (Additional file 1). The first of these clusters is almost exclusively comprised of bacterial, chloroplast and mitochondrial MtRSs. Within this group several monophyletic bacterial lineages can be recognized such as cyanobacteria (including the chloroplast versions), firmicutes and various proteobacteria. This group is unified by the presence of a single Zn-ribbon inserted into the catalytic domain (Additional file 1). The second cluster includes the conventional MtRS from almost all archaea, eukaryotes (cytoplasmic) and certain bacterial lineages, such as actinobacteria, chloroflexi, spirochetes and bacteroidetes. This group is typified by presence of an insert with two Zn-ribbons or a variant thereof, i.e. a circularly permutated Zn-ribbon arising from the loss of the N- and C- terminal metal-chelating dyads, respectively of the first and second Zn ribbons (Additional file 1). This phylogenetic picture of the conventional MtRSs suggests that the internal long-branch represents the primary split between the archaeo-eukaryotic and bacterial lineages, with subsequent lateral transfer of the archaeo-eukaryotic versions to certain bacterial lineages along with xenologous gene-displacement [15]. The newly identified paralogous MtRSs, which are fused to or associated with the metal-binding cupin domain, are only found in bacteria and form a distinct cluster grouping with the "archaeo-eukaryotic" type MtRSs, albeit separated by a long branch (Additional file 1). This grouping is supported by the fact that they possess a duplicated segment-swapped Zn-ribbon, just like the "archaeo-eukaryotic" type MtRSs. This paralogous MtRS is widely distributed in various bacterial lineages, namely actinobacteria, proteobacteria and firmicutes. In particular, it is frequently found in diverse actinobacterial species, with *Salinispora arenicola *having three and *Actinosynnema mirum *two distinct paralogs. Interestingly, it is also found in several distantly related pathogenic or symbiotic bacteria that are known to secrete compounds into host cells: *Ralstonia solanacearum *(two paralogous versions), *Burkholderia phytofirmans*, multiple *Dickeya *species, *Erwinia pyrifoliae, Pseudomonas syringae*, *Frankia *species, *Sinorhizobium meliloti *and *Mesorhizobium loti*, which associate with plants and *Yersinia pseudotuberculosis, Photorhabdus luminescens, Pseudomonas entomophila, Bacillus cereus *and *Legionella pneumophila*, which infect animals. The affinities of this paralogous group, suggest that it was probably founded by an independent lateral transfer, perhaps from an archaeal source, into a bacterial lineage. Given its wide presence in actinobacteria, it is likely that this bacterial lineage was the actinobacterial lineage, from where it dispersed widely through lateral transfer to several distinct firimicute and proteobacteria lineages, probably in shared environments.

### Contextual evidence suggests that the paralogous methionyl-tRNA synthetases might catalyze synthesis of a novel peptide

To better understand the biosynthetic pathway wherein this MtRS paralog functions we resorted to genomic neighborhood analysis, which, along with domain fusions, has been shown to be a powerful tool to infer functions of uncharacterized proteins [22]. This group of MtRS paralogs is particularly suited for such analysis as they are highly mobile in evolutionary terms and any gene-neighborhood associations that are detected between distantly related species are likely to be indicative of functionally relevant interactions. We found two gene neighborhood/domain-fusion associations that occur without exception with all these MtRS paralogs (Figure 2): 1) the above-noted association with the metal-binding cupin. 2) A neighborhood association with a gene-encoding L-lysine 6-monooxygenase, a Rossmann fold oxidoreductase that catalyzes the NADPH-dependent hydroxylation of lysine at the N6 position. The N6-hydroxy-lysine is an intermediate in the biosynthesis of a non-ribosomally condensed peptide-derived siderophore, aerobactin [23]. In aerobactin biosynthesis the N6 position is further modified by acetylation by an acyltransferase of the GNAT fold (Figure 3). Interestingly, we found that several MtRS paralog gene neighborhoods additionally encode a member of the GNAT superfamily indicating that a similar reaction is likely even in this system (Figures 2 and 3). These N6 modifications of lysine serve to block the ε-NH_2 _group, thereby favoring dipeptide condensation utilizing the main-chain α-NH_2 _by the aerobactin synthetase that belongs to the protein kinase fold [5]. The NH_2 _group of glucosamine, which is cysteinylated by CtRS in mycothiol synthesis, is also initially blocked by an acetyl group (i.e. as N-acetyl-glucosamine) prior to amide formation [10]. Given that modifications of lysine N6 comparable to those seen in aerobactin biosynthesis are predicted to be catalyzed by enzymes encoded in the MtRS paralog gene neighborhoods, we reasoned that the lysine N6 is likely to be modified similarly for peptide bond formation even in this system (Figure 3). This also suggests that the other enzyme common to all these neighborhoods, the MtRS paralog, by analogy to the CDPSs and the cysteinyl ligase in mycothiol biosynthesis, is likely to catalyze formation of a peptide bond in this system (Figure 3). Thus, we propose that the core of the biochemical pathway specified by this conserved gene neighborhood involves the synthesis of a dipeptide through the condensation of the adenylated carboxyl group of methionine with the α-NH_2 _group of a lysine derivative (i.e. modified at the N6 position). The presence of a gene encoding acireductone dioxygenase, an enzyme involved in methionine salvage, in a subset of these gene-neighborhoods is also consistent with methionine being channeled into this metabolite (Figure 2).

In some organisms there are gene neighborhood associations suggestive of potential variations to the theme of modification of the N6 lysine. A subset of these predicted operons (e.g. in *Rhodococcus jostii *and two of the three paralogous gene-neighborhoods in *Salinispora arenicola*) also contain a tightly linked saccharopine dehydrogenase gene (Figure 2). This enzyme catalyzes the formation of saccharopine by linking 2-oxoglutarate to N6 of lysine. Thus, the modification of the ε-NH_2 _by this enzyme might effectively be similar to the acetylation of this position (Figure 3). Further, several of the predicted operons encode proteins such as (Figure 2): 1) acyl-coA ligase which ligates coA to a fatty acid [18]; 2) one or more acyl carrier proteins (ACP) that bear a serine-linked phosphopantetheinyl moiety, which in turn carries an fatty acyl group as a thioester; 3) Acyl condensation enzymes, which catalyze the condensation of an acyl-coA to another moiety resulting in an elongated chain due to addition of the acyl element; 4) enzymes that could catalyze a transacylase reaction that delinks the fatty acid from the ACP or transesterifies it. These transacylase-like enzymes might belong to the previously recognized NTN-hydrolase superfamily or the α/β-hydrolase superfamily or the BtrH family with a papain-like fold [5], or are representatives of a novel family of proteins that we determined as also belonging to the papain-like fold (Additional file 1); 4) Acyl-coA dehydrogenases, which catalyze the modification of fatty acids via desaturation. These genes are present in the neighborhoods only if the gene cluster also encodes a member of the GNAT fold, suggesting that they might be involved in synthesis of an acyl-coA substrate that could be used by the GNAT enzyme (in place of the default acetyl-coA) to modify the N6 position of lysine. However, in principle the ACP and associated enzymes could also be used as a substrate for attachment of the peptide synthesized by this system, comparable to what is observed in butirosin biosynthesis and peptide synthesis by giant multidomain peptide synthetases [24,25]. The other universally present component of this system, the metal-binding cupin, belongs to a large radiation of such enzymes, which comprise of a single double-stranded β-helix domain with four conserved positions involved in chelating a metal ion [17,26]. They catalyze two distinct types of reactions: 1) isomerization of linearized sugars through an enediol intermediate and 2) a dioxygenase reaction, incorporating two oxygens into the substrates (e.g. cysteine dioxygenase, wherein the SH group of cysteine is oxidized to sulfinate). However, unlike the 2-oxoglutarate-dependent dioxygenases of the double-stranded β-helix fold they are not known to catalyze single hydroxylations of substrates [17]. Given that this cupin is tightly associated with the MtRS (either fused or typically as the gene 5' to the MtRS gene (Figure 2), it is possible that it functions in close association with MtRS, perhaps catalyzing a reaction on methionine (Figure 3). However, the exact nature of this modification remains unclear as there are currently no precedents for such modifications in other characterized peptide modification systems.

Beyond the conserved core, majority of these neighborhoods encode several other enzymatic domains and transporters and peptide-binding proteins of the periplasmic binding protein superfamilies. While these tend to vary between neighborhoods, their close genomic linkage and predicted biochemistry suggests that they are part of the same biosynthetic pathway. One or more genes encoding ATP-grasp enzymes or multi-domain non-ribosomal peptide synthases with condensation domains are encountered in several of these operons. By analogy to other non-ribosomal peptide synthesis operons these enzymes are likely to catalyze the ligation of additional amino acids [5]. Consistent with this, enzymes for synthesis of other amino acids are also encountered in these operons (Figure 2, Additional file 1). These include a PP-loop ATPase that is closely related to the asparagine synthetase, suggesting that it might catalyze formation of asparagine. Furthermore, two ornithine generating enzymes, namely arginase and glycine amidinotransferase, proline-generating ornithine cyclodeaminase, citrulline-generating dimethylargininase and different PLPDEs are also encoded by some of these neighborhoods. The latter enzymes include the diaminobutyrate transaminase, which synthesizes 2,3-diaminobutyrate, and cysteine synthase which generates cysteine. It is possible that amino acids generated by the action of these enzymes are incorporated as further residues of the peptide or alternatively they modify the peptide via reactions such as decarboxylation (e.g. as catalyzed by the PLPDE, BtrK, in butirosin biosynthesis [24]). Other prevalent enzymes encoded by these predicted operons are diverse redox enzymes belonging to distinct folds (Figure 2): 1) First of these are Rossmann fold dehydrogenases which utilize either FAD or NADH cofactors. 2) Members of the flavin- or F420- dependent monooxygenase superfamily, which includes the monoxygenase BtrO that hydroxylates the amino acid side-chain in butirosin biosynthesis [24]. 3) double-stranded β-helix-fold 2-oxoglutarate- dependent dioxygenases, such as members of the JOR/JmjC superfamily, and the phytanoyl hydroxylase family of classical 2OGFeDOs [17]. 4) SnoaB/ActVA-Orf6-type ferredoxin-fold monooxygenases that catalyze the insertion of a single oxygen atom into substrates and are often found in the biosynthetic pathways of several antibiotics [27]. 5) α-helical diiron oxygenases of the heme oxygenase superfamily [28]. The last 4 classes of above enzymes could in particular catalyze hydroxylation or oxygenation of side chains of the peptide and/or the fatty acyl moiety if present. Thus, in conclusion majority of these systems centered on the paralogous MtRS are predicted to catalyze synthesis of a highly oxygenated derivative of the dipeptide Met-Lys, which in some cases might be further extended with additional residues.

The presence of this system in diverse actinobacteria, which are known to produce diverse secondary metabolites, suggests that this peptide derivative might function as an antibiotic. However, outside of actinomyctes, it is primarily found in bacteria showing symbiotic or parasitic associations with eukaryotic cells, which normally do not encode any antibiotic production systems. In these cases it is likely that this peptide metabolite has a role in host-parasite interactions. Consistent with this, in these organisms the predicted operons usually contain a peptide-binding protein and a transmembrane efflux transporter (Figure 2). One possibility is that it functions as a siderophore in these cases, with the variability probably arising due to selection against siderophore-stealing or host immunity.

### Identification of a parallel peptide synthesis system further supports the role of the MtRS paralog in peptide ligation

We also identified another set of predicted operons that closely paralleled the above system in diverse firmicutes, cyanobacteria, proteobacteria and actinobacteria (Figure 2). These were centered on a distinctive protein that combines a cupin domain, related to those fused or associated with the MtRS, with an N-terminal uncharacterized region (e.g. FRAAL4157, gi: 111223558). Iterative sequence searches seeded with this N-terminal region using the PSI-BLAST program recovered significant matches to the heme oxygenase superfamily of diiron oxygenases that were also detected in the above system (e.g. *Frankia *FRAAL4157 recovers the experimentally characterized oxygenase Ct610, PDB: 1RCW from *Chlamydia trachomatis*, e = 10^-3^, iteration 8). The matches showed that the N-terminal region of these proteins contain two diiron oxygenase domains - the N-terminal one predicted to be inactive and the C-terminal predicted to be active based on conservation of the iron-chelating histidines and acidic residues [28]. Thus, these proteins are predicted to possess two distinct oxygenase capabilities, with the diiron oxygenase domain probably functioning as a monoxygenase and the C-terminal cupin domain as a dioxygenase. They are encoded in predicted operons that show three related but distinct themes (Figure 2): 1) the simplest of these combine the gene encoding the oxygenase-cupin protein with one or two enzymes involved in modifying amino acids such as an amino acid methylase and a PLPDE. 2) The second set of these predicted operons combine the oxygenase-cupin gene with genes encoding one or two peptide ligases of the ATP-grasp fold [5]. Additionally, these operons encode a Rieske 2Fe-2 S iron-sulfur protein involved in electron transport in redox reactions. 3) The final set of predicted operons is similar to the above operons - in place of the ATP-grasp peptide ligases they encode giant multi-domain non-ribosomal peptide synthetases with condensation domains and also acyl-coA ligases, which could charge amino acids with coA for use by the former enzymes. This set of operons also encodes a protein of the uncharacterized "YqcI/YcgG" superfamily that contains an absolutely conserved N-terminal cysteine (Additional file 1). Given that the multi-domain non-ribosomal peptide synthetases might utilize a protein anchor for elongation of peptides, it is conceivable that the conserved cysteine of this family serves as a means for anchoring the initial residue via a thiocarboxylate linkage. Furthermore, all the three versions of these operons might additionally encode a transmembrane efflux transporter, suggesting that the metabolite synthesized by this operon is deployed in the environment (Figure 2). It is possible that in these gene-clusters, the cupin might play a role similar to the cognate cupin in the systems centered on the MtRS paralog, whereas the diiron oxygenase could function similarly to the lysine N6-monoxygenase. The simplest of these predicted operons are likely to modify a single amino acid. Those containing either of the two unrelated types of peptide-bond forming enzymes appear to be analogs of the system centered on the MtRS paralog with distinct oxygenases associated with at least one peptide ligase.

## General conclusions

Previous studies have pointed to the utilization of tRNAs and AAtRS in bacteria for the synthesis of diverse metabolites [1,10]. In the current work we present evidence that AAtRS are more widely used in such processes than has been previously appreciated. The examples uncovered in this study help illustrate the spectrum of exaptation of the AAtRS in non-ribosomal metabolite biosynthesis. Thus, we can now see a complete progression in the synthetic processes involving these enzymes: 1) Conventional AAtRS that ligate amino acids to tRNAs to be used by GNAT fold enzymes in peptidoglycan and N-end rule peptide ligations. Here the regular AAtRS involved in protein synthesis provide AAtRNA substrates to be used by peptide ligases. 2) Closely related paralogous AAtRS are dedicated to provide AAtRNAs for particular pathways. The membrane-associated bacterial KtRS are involved in lipid aminoacylation whereas the paralogous StRS VlmL is deployed in valinamycin biosynthesis. Both these class-II AAtRS merely serve in charging their cognate amino acids with tRNAs to be utilized by MprF-like peptide ligases of the GNAT fold. Further evidence for such paralogous class-II AAtRS functioning with MprF fold enzymes is provided by a novel group of orthologous proteins encoded by several ascomycete and basidiomycete fungi (e.g. *Aspergillus nidulans *AN0314.2, gi:67516065 [Additional file 1]). These proteins combine an N-terminal aspartyl tRNA synthetase module with a C-terminal MprF-like peptide ligase and might be required for the synthesis of a fungus-specific peptide metabolite. Similarly, a glutamyl tRNA synthetase paralog is deployed in synthesis of δ-aminolevulinic acid, a precursor for porphyrins [1]. 3) The paralogous CtRS involved in mycothiol synthesis and the paralogous MtRS. The example of the paralogous MtRS-based biosynthetic systems identified in this study points to the generality of the principle that was first presented by the CtRS paralog that functions as a cysteine ligase in mycothiol synthesis. Both these class-I AAtRSs are likely to represent a further development on the previous theme wherein they have become peptide ligases themselves. However, these enzymes, like the above enzymes, have diverged only to a limited degree relative to their "universal" paralogs involved in protein synthesis. 4) The CDPSs. The finding that the CDPSs are likely to be derived from a class-I AAtRS catalytic domain indicates the unexpected degree of divergence that might occur in these enzymatic domains while retaining the basic catalytic properties. Here, the catalytic domain has not only diverged greatly from that of the original class-I AAtRS, but it has also acquired new active site residues, converting it into a self-contained cyclic peptide forming enzyme, which can use charged amino acids, either of same or different type as substrates.

More generally this pattern of recruitment and diversification of the AAtRS mirrors what has been previously observed with other ligase domains, such as the ubiquitin E1-ligase-like, ATP-grasp, glutamine synthetase-like NH2-COOH ligase, GNAT fold, protein kinase-like and condensation domains [5]. In particular, the niche provided by the development of secondary metabolism for synthesis of antibiotics, siderophores, soluble cell-cell signals and host-interaction molecules allowed for the extensive diversification of these enzymes. These metabolites are under constant selection due to development of resistance, siderophore-stealing and host immunity, which favors the emergence of biosynthetic diversity. Concomitant to the diversification of ligases, there was also a similar explosion in the diversity of peptide-modifying enzymatic domains [5,17]. In particular, systems such as those described here and elsewhere [17] point to the recruitment of a great variety of oxygenases belonging to unrelated protein folds that utilize molecular oxygen. This suggests that the emergence of the extant type of bacterial secondary metabolism systems received a tremendous impetus from the primary oxygenation event in Earth's history caused by cyanobacterial photosynthesis [17]. The metal-binding cupins identified in the current study are a potential example of the evolutionary transition of an ancestrally sugar-binding double-stranded β-helix domain to an amino acid/peptide modifying enzyme. This latter activity was greatly developed in the 2-oxoglutarate-dependent enzymes that emerged within this fold [17].

In conclusion, we hope that the identification of these novel systems might spur further experimental investigations to test the presented hypothesis and to fill in the details of the biochemistry of the enzymes described here. Given the phyletic distribution of the systems described here, such studies are likely to be of potential importance to uncover novel metabolites and aspects of host-parasite interactions.

## Abbreviations

AATRS: aminoacyl-tRNA synthetases; AATRNA: aminoacyl tRNA; CDPS: cyclic dipeptide synthases: cyclodipeptide synthases; CDP: cyclic dipeptide; CTRS: cysteinyl-tRNA synthetase; CYY: cyclic dityrosine; GNAT: GCN5-like acetyltransferase; HUP: HIGH: UspA and Photolyase/PP-loop superclass of Rossmannoid nucleotidyl transferases containing the HIGH-motif; MTRS: methionyl-tRNA synthetase; PLPDE: pyridoxal phosphate dependent enzyme; YTRS: tyrosine-tRNA synthetase; WTRS: tryptophan-tRNA synthetase. **Gene and protein abbreviations**: 2OGFEDO: 2-oxoglutarate and iron(II)-dependent diooxygenase; AA_methylase: Rossmann-fold methyltransferases; ABC or ABC_FeTrans: ABC iron transporter ATPase/permease; APA: APA family antiporter; B3/4_FtRS: B3/4 domain of tRS beta subunits; EFffl_Trans: aminoacid efflux transporter; FADOR or FADOR/MOO: FAD-dependent oxidoreductase; FMN_binding: flavin reductase-like domain; VlmB: AurF-like ferretin oxidase; GAT2: glutamine amidotransferases class-II; HAD or HAD_FkbH: haloacid dehalogenase-like hydrolase; Hiskin+rec: signal-transduction one component system; K_OH/OR: L-lysine 6-monooxygenase (NADPH); Mur_ligase: Mur family glutamate ligase; NRPSD: non-ribosomal peptide synthase domains; NTN transacylase: acyl-coenzyme A:6-aminopenicillanic acid acyl-transferase; New_papain: novel family of papain-like cysteine proteases - described in this study; OxRed: OxRed2 or OxRed4_MO, oxidoreductases of diverse families and folds; PBP: periplasmic binding protein; PLPDE: pyridoxal-phosphate dependent enzyme; AsnSyn: PP-loop asparagine synthetase family; RRM_fold_MOO: RRM fold monooxygenase; Rieske: 2Fe-2 S oxireductase; Rossmann_OR: Rossmann fold oxireductase; SelA_PLPDE: L-seryl-tRNA selenium transferase; SnoaL_PC and SnoaL_PC_like: SnoaL-like polyketide cyclase and similar proteins; TM: proteins with transmembrane regions; Thi5_like: NMT1/THI5 family; VmlR: VlmR flavin reductase; AmidinoTase: amidinotransferases/arginine deiminases; prot_methylase: protein arginine N-methyltransferase. **Species abbreviations**: Amir: *Actinosynnema mirum*; Aper: *Aeropyrum pernix*; Bcer: *Bacillus cereus*; Blic: *Bacillus licheniformis*; Bsp.: *Burkholderia *sp.; Bsub: *Bacillus subtilis*; Bthu: *Bacillus thuringiensis*; CPro: Candidatus *Protochlamydia*; Cjei: *Corynebacterium jeikeium*; Daes: *Desulfovibrio aespoeensis*; Ecol: *Escherichia coli*; Gste: *Geobacillus stearothermophilus*; Gzea: *Gibberella zeae*; Llon: *Legionella longbeachae*; Mbov: *Mycobacterium bovis*; Mtub: *Mycobacterium tuberculosis*; Ndas: *Nocardiopsis dassonvillei*; Nvec: *Nematostella vectensis*; Paca: *Parachlamydia acanthamoebae*; Pflu: *Pseudomonas fluorescens*; Phor: *Pyrococcus horikoshii*; Plum: *Photorhabdus luminescens*; Rgry: *Rickettsiella grylli*; Scer: *Saccharomyces cerevisiae*; Shae: *Staphylococcus haemolyticus*; Sjap: *Sphingobium japonicum*; Snou: *Streptomyces noursei*; Ssp.: *Streptomyces *sp.

## Competing interests

The authors declare that they have no competing interests.

## Authors' contributions

LA, RFS and LMI performed the reported research and wrote the paper. All authors read and approved the final manuscript.

## Reviewer's Comments

### Andrei Osterman, Burnham Institute, La Jolla, CA, United States

A new study by L. Aravind and colleagues on evolution and functional diversification of the aminoacyl-tRNA synthetase (AAtRS) family is truly fascinating in a number of ways. Using a sophisticated comparative genomics approach, which combines the distant homology and genomic context analysis, they substantially expanded the boundaries of this family including a discovery of its previously unrecognized relationship with cyclodipeptide synthetases (CDPS). This discovery provided crucial evolutionary and mechanistic insights into this "lost tribe" of the AA-tRS clan and demonstrated an amazing functional versatility of the respective fold. The insightful analysis of conserved chromosomal clusters associated with some of the uncharacterized CDPSs opened a Pandora's box of completely unknown and unforeseen biochemical transformation generating a plethora of novel secondary metabolites. Experimental elucidation of these new pathways and their products, which has been enabled by this study, would strongly impact our knowledge of various signaling systems (e.g. host-pathogen interactions) and lead to a discovery of new classes of bioactive compounds. However, as if it was not enough, this paper takes us further, to the analysis of a widespread and extremely interesting family of Met-tRS paralogs, which leads to further expansion of a biochemical landscape of nonribosomal peptidoids. As in the CDPS case, an ingenious combination of phylogenetic and genomic context analyses (already a standard in the field) with fully innovative chemical reasoning allows Aravind et al. to build a very solid case for a number of predicted biosynthetic pathways. The predicted pathways share common themes (eg MettRS-driven condensation of Met and Lys) accompanied by species-specific variations (novel oxidative and nonoxidative modifications of both amino acids). Going back to the overall impact of this paper, one can't help notice that it is not only another triumphant example of creative comparative genomics contributing to better understanding of an important protein family. Its tremendous added value is in providing an incredible opportunity for a small army of experimental biochemists to uncover new processes and molecules with tremendous biomedical potential.

### Igor Zhulin, University of Tennessee, Oak Ridge National Laboratory

This reviewer had no comments.

## Supplementary Material

Additional file 1 **Alignments and contextual information for AAtRS paralogs and associated proteins**. Materials and methods, comprehensive multiple sequence alignments of domains described in the text, the MtRS phylogenetic tree and all comprehensive gene neighborhoods of the protein families described in the text. These can be accessed at: ftp://ftp.ncbi.nih.gov/pub/aravind/AATRS/Supplementary_material.html Click here for file
