# Supplementary material for: Predicted class-I aminoacyl tRNA synthetase-like proteins in non-ribosomal peptide synthesis
Source: Biol Direct. 2010 Aug 2;5:48. doi: 10.1186/1745-6150-5-48 (PMC2922099; doi:10.1186/1745-6150-5-48)
Supplement: Additional file 1 — Alignments and contextual information for AAtRS paralogs and associated proteins. Materials and methods, comprehensive multiple sequence alignments of domains described in the text, the MtRS phylogenetic tree and all comprehensive gene neighborhoods of the protein families described in the text. These can be accessed at: ftp://ftp.ncbi.nih.gov/pub/aravind/AATRS/Supplementary_material.html [file 1745-6150-5-48-S1.HTML]

SUPPLEMENTARY MATERIAL FOR: Identification of novel class-I aminoacyl-tRNA synthetase domains that function as non-ribosomal peptide ligases


  


**SUPPLEMENTARY MATERIAL
  
Identification of novel class-I aminoacyl-tRNA synthetase domains that function as non-ribosomal peptide ligases**
  
  

L. Aravind\*,
Robson F. de Souza and
Lakshminarayan M Iyer
  
  
\*

*Address for correspondence: L. Aravind (aravind@mail.nih.gov)*
  
  
*National Center for Biotechnology Information, National Library of Medicine, National Institutes of Health, Bethesda, MD 20894, USA*
  
  
  
  


---

**ABSTRACT**  

Recent studies point to a great diversity of non-ribosomal peptide synthesis systems with major roles in amino acid
and co-factor biosynthesis, secondary metabolism, and peptide modifications of proteins. The least studied of these
systems are those utilizing tRNAs or aminoacyl-tRNA synthetases (AAtRS) in non-ribosomal peptide ligation. Here we
describe novel examples of AAtRS-derived peptide synthesis systems that are likely to be involved in the synthesis
of widely distributed peptide-derived metabolites. Firstly, using sensitive sequence profile methods we show that
the cyclic dipeptide synthases (CDPSs) are members of the HUP class of Rossmannoid domains and are likely to be
highly derived versions of the class-I tRNA synthetase catalytic domains. We also identify the first eukaryotic
CDPSs in fungi and in animals, where they might be involved in the immune response. We also identify a paralogous
version of the methionyl-tRNA synthetase that is widespread in bacteria and present evidence using contextual
information that it functions independently of protein synthesis as a peptide ligase in the synthesis of a peptide
derived secondary metabolite. We show that this metabolite is likely to be heavily modified through oxygenations
catalyzed by a metal-binding cupin domain and a lysine N6 monooxygenase that are strictly associated with this
paralogous methionyl-tRNA synthetase (MtRS). Furthermore, we also identify an analogous system wherein the MtRS has
been replaced by more typical peptide ligases of the ATP-grasp and modular condensation-domain type. The prevalence
of these predicted biosynthetic pathways in phylogenetically distant pathogenic and symbiotic bacteria that are
intracellular or closely associated with animal or plant cells suggests that the metabolites synthesized by them
might have a major role in host-bacterium interactions. More generally, these findings point to a complete spectrum
of recruitment of AAtRS to the various non-ribosomal biosynthetic pathways, which spans the conventional synthetases
providing AAtRNAs for peptidoglycan and N-end rule peptide ligations, closely related AAtRS paralogs functioning as
peptide ligases or dedicated tRNA-charging enzymes in pathways such as that identified in this study and mycothiol,
tetrapyrrole and valinamycin pathways, and highly derived versions of the class-I AAtRS catalytic domain like the
CDPSs.


---

### **Contents**

1. Materials and Methods  
   - Phyletic distribution and gene-neighborhoods of various peptide ligases described in the study  
     - Phyletic distribution and gene neighborhoods of AlbC-like family [svg file with links], [textfile]  
       - Domain architectures, phyletic distribution and gene neighborhoods of the methionyl-tRNA synthetase paralogs [svg file with links] , [textfile]  
         - Domain architectures, phyletic distribution and gene neighborhoods for the cupins fused to alpha-helical diiron-monooxygenases [svg file with links] , [textfile]  
           - **Note added in proof:** gene neighborhood of paralogous Seryl-tRNA synthetase and alignment of the predicted enzymatic domain of Atu2574-like proteins [svg file with links] , [textfile]- Comprehensive multiple alignment of CDPS and AAtRS catalytic cores  
       - Phylogenetic tree of archaeal and bacterial methionyl-tRNA synthetases and paralogs  
         - Alignment of the archaeal and bacterial methionyl-tRNA synthetases and paralogs  
           - Alignment of members of the novel papain-like family  
             - Alignment of members of the YqcI/YcgG family  
               - List and domain architectures of MPRF-like acetyltransferases in Bacteria and Eukaryotes  
                 - List of species abbreviations used in MtRS phylogeny
  


---


**1. MATERIALS AND METHODS**
  


Profile searches were conducted using the PSI-BLAST program (Altschul *et al*., 1997) with a default profile inclusion expectation (E) value
threshold of 0.01. Profile-profile comparisons were performed using the HHpred program (S�ding, 2005; S�ding *et al.*, 2005). HMM searches were
conducted using JACKHMMER from the HMMER3 package (Eddy, 1998). Multiple alignments were constructed using Kalign (Lassman and Sonnhammer, 2005)
followed by manual adjustments based on PSI-BLAST results. Protein secondary structure was predicted using a multiple alignment as the input for
the JPRED program (Cuff *et al.*, 1998). Three dimensional structures were rendered using the PYMOL program (http://www.pymol.org/).

References  

- Altschul, S.F., Madden, T.L., Schaffer, A.A., Zhang, J., Zhang, Z., Miller, W. and Lipman, D.J. (1997) Gapped BLAST and PSI-BLAST: a new generation of protein database search programs, Nucleic Acids Res, 25, 3389-3402.  
  - Cuff, J.A., Clamp, M.E., Siddiqui, A.S., Finlay, M. and Barton, G.J. (1998) JPred: a consensus secondary structure prediction server, Bioinformatics, 14, 892-893.  
    - Eddy, S.R. (1998) Profile hidden Markov models, Bioinformatics, 14, 755-763.  
      - Lassmann, T. and Sonnhammer, E.L. (2005) Kalign--an accurate and fast multiple sequence alignment algorithm, BMC Bioinformatics, 6, 298.  
        - S�ding J. (2005) Protein homology detection by HMM-HMM comparison.Bioinformatics 21, 951-960. doi:10.1093/bioinformatics/bti125.- S�ding J, Biegert A, and Lupas AN. (2005) The HHpred interactive server for protein homology detection and structure prediction. Nucleic Acids Research 33, W244--W248 (Web Server issue). doi:10.1093/nar/gki40.


---


3. Comprehensive multiple alignment of CDPS and AAtRS catalytic cores
  

```
Secondary_structure_assignments       Str-1         Hel-1---------------->           Str-2	                                                       Hel-2                   Str-3              <--Insert shared by CDPS and tRNA syn------------>        Hel-3                      Str-4           Hel-4                    Str-5
TrpS_3a04.pdb                        EEEEEEE--------HHHH----HH-HHHHHHHHHHH----------EEEEEEH-HHHHHHH----H----------------------------------------HHHHHHHHHHHHHHHHHHHH-----------EEEEE-----------HHHHHHHHHHHH---HHHHHHHHH--------------------------------HHHHHHHHHHHHHHHHH---------------EEEEEEEE-------HHHHHHHHHHHH---------------EEEEEE-----
1i6mA.pdb                            EEEEEEE--------HHHH----HHHHHHHHHHHH------------EEEEEEH-HHHHH-----------------------------------------------HHHHHHHHHHHHHHHHHHH------------EEEEE-------------HHHHHHHHHHHHHHHHHHHHHHHHHHHHH-------------------------HHHHHHHHHHHHHHHHH----------------EEEEE---------HHHHHHHHHHHHHHhh-----------EEE--------
Secondary_structure_prediction       -EEEEEE--------HHHH----HHHHHHHHHHHHH-----------EEEEEEE-HHHHHHHHH-------------------------------------------HHHHHHHHHHHHHHHHHH------------EEEEEE------------HHHHHHHHHH--HHHHHHHHH------------------------------------HHHHHHHHHHHHHH----------------EEEEEEEE--------HHHHHHHHHHH---------------EEEEE------
NdasDRAFT_4974_Ndas_229209413        EHALIGISA-GNS-YFSQE----RIAQLLRWAQRHFA----------EVDVLYA-DLHLDTMYM-ASGGSREH----------------------------------ASSRANRALKDVRRRIRRAVEAAA-PDAGNVRVRALSQCT------GLPGYRDVAYRLDREHATDPRVRRACEEHVRHVIGA-QP---------------DPDGARLRAGLAYLRAELPLLLSTPRVLGLPSS--VCCYHALMPI-----LSRLRGATSCFHP-----------GQGHVILRPVDRHL\CDPS
albC_Snou_27447466                   EHALIGISA-GNS-YFSQK----NTVMLLQWAGQRFE----------RTDVVYV-DTHIDEMLI-ADGRSAQE----------------------------------AERSVKRTLKDLRRRLRRSLESVG-DHAERFRVRSLSELQ------ETPEYRAVRERTDRAFEEDAEFATACEDMVRAVVMN-RPGDGV-----------GISAEHLRAGLNYVLAEAPLFADSPGVFSVPSS--VLCYHIDTPI-----TAFLSRRETGFRAA---------EGQAYVVVRPQELAD|
Amir_4627_Amir_256378646             EHVVFGVSP-GNS-YFQVP----RMAELFGWLRGESD----------RIDVVIP-DSALVHTYL-ALGYEERR----------------------------------AERKARAEVNVLRNRVSRAWEAAG-GPRPGDGLNLMSELE------GGEVYRARLAECERALREDEVLRGTSAEMSREVLAL-KGHRG------------MASDEQVERAMRYLLAELPFFLASSEIFDVPTS--VNFYHRKLPL-----AEVVFSGESLLRAS---------PRQAYATIRPVG---|
yvmC_Bsub_16080560                   RHVLVGISP-FNS-RFSED----YIYRLIGWAKAQFK----------SVSVLLA-GHEAANLLE-ALGTPRGK----------------------------------AERKVRKEVSRNRRFAERALVAHG---GDPKAIHTFSDFI------DNKAYQLLRQEVEHAFFEQPHFRHACLDMSREAIIG-RARGVSLMME-------EVSEDMLNLAVEYVIAELPFFIGAPDILEVEET--LLAYHRPWKL-----GEKISNHEFSICMR---------PNQGYLIVQEMAQML|
bcere0004_52720_Bcer_229187741       NHALIGISP-FNS-RFSDE----YINRLIEWALHTFD----------DVSVLLA-GKEAANLLE-ALGTPKGK----------------------------------AERKVRKEVSRNRRSAEKALKEHG---GNINAIHTFSDFN------DNYAYNNMRTEAEHAFVNEPHFRNACLEMSHAAILG-RARGTNIDID-------QISSDMLNIAVEYVIAELPFFIGGAEILGIEET--VLIYHKPWEL-----GERIVRNDFSIKMK---------PNQGYLMVQEMEHMC|
bthur0001_56070_Bthu_228988985       NHALIGISP-FNS-RFSDE----YINRLIKWALHTFD----------GVSVLLA-GKEAANLLE-ALGTPKGK----------------------------------AERKVRKEVSRNRRSAEKALIEHG---GDVNAIYTFSDFN------DNNAYNCMRAEAEHNFLNETHFRNACLEMSHAAILG-RARGTNIDID-------QISNDMLNIAVEYVIAELPFFIGGAEILGTQET--VLIYHKPWEL-----GEQIVRNDFSIKMK---------PNQGYLMVQETEDLS|
yvmC_Blic_52081867                   RHVLVGISP-FNS-RFSED----YIHRLIAWAVREFQ----------SVSVLLA-GKEAANLLE-ALGTPHGK----------------------------------AERKVRKEVSRNRRFAEKALEAHG---GNPEDIHTFSDFA------NQTAYRNLRMEVEAAFFDQTHFRNACLEMSHAAILG-RARGTRMDVV-------EVSADMLELAVEYVIAELPFFIAAPDILGVEET--LLAYHRPWKL-----GEQISRNEFAVKMR---------PNQGYLMVSEADERV|
RBTH_07362_Bthu_75758511             KHAIIGISP-FNS-RFSDE----YINRLIEWALHTFD----------DVSVLLA-GKEAANLLE-ALGH-QSK----------------------------------RKEKLGKKISRNRRSAEKALKEHG---GNVNAIHTFSDFN------DNNAYSCMRAEAEHIFLSETVFRNACLEMSHAAILG-RARGTNIDID-------QISNDMLNIAVEYVIAELPFFIGGAEILGTQEA--VLIYHKPWEL-----GEQIVRNDFSIRMK---------PNQGYLMVQEMENLS|
Rv2275_Mtub_15609412                 DHAVIGVSP-GNS-YFSRQ----RLRDLGLWGLTNFD----------RVDFVYT-DVHVAESYE-ALGDSAIE----------------------------------ARRKAVKNIRGVRAKITTTVNELD-PAGARLCVRPMSEFQ------SNEAYRELHADLLTRLKDDEDLRAVCQDLVRRFLST-KVGPRQ-----------GATATQEQVCMDYICAEAPLFLDTPAILGVPSS--LNCYHQSLPL-----AEMLYARGSGLRAS---------RNQGHAIVTPDGSPA|
BCG_2292_Mbov_121638157              DHAVIGVSP-GNS-YFSRQ----RLRDLGLWGLTNFD----------RVDFVYT-DVHVAESYE-ALGDSAIE----------------------------------ARRKAVKNIRGVRAKITTTVNELD-PAGARLCVRPMSEFQ------SNEAYRELHADLLTRLKDDEDLRAVCQDLVRRFLST-KVGPRQ-----------GATATQEQVCMDYICAEAPLFLDTPAILGVPSS--LNCYHQSLPL-----AAMLYARGSGLRAS---------RNQGHAIVTPDGSPA|
StAA4_010100004850_Ssp._256666447    RHAVFGVSP-GNS-YFNVD----RLRSTLDWLQTEFK----------QIDVIIP-DSALKHTFI-ALGYDEPK----------------------------------AAKKARSETNVLRNRVLRAWTALA-GPRPTDGLHRMSELQ------ADPVYTEVLAHCAQLTATDPALAAASTEMTQEVLAA-KGLTGE-----------A-TPAQLAEAKKYLIAELPFFVSSCRIFDVDAS--LNFYHQPLPL-----ANVIFSGASALKPD---------PGQGYATIRPA----|
pSHaeC06_Shae_68535056               KHVILGISP-FTS-KYNES----YIRKIIQWANSNFD----------DFSILLA-GEESKNLLE-CLGYSSSK----------------------------------ANQKVRKEIKRQIRFCEDEIIKCN--KTITNRIHRFSDFK------NNIYYIDIYKTIVDQFNTDSNFKNSCLKMSLQALQS-KGKNVNTSI--------EITDETLEYAAQYVLAELPFFLNANPIINTQET--LMAYHAPWEL-----GTNIINDQFNLKMN---------EKQGYIILTEKGDNY|
bthur0014_63370_Bthu_228905252       KHAIIGISP-FNS-RFSDE----YINRLIEWALHTFD----------DVSVLLA-GKEAANLLE-ALGTPKGK----------------------------------AERKVRKEVSRNRRSAEKALKEHG---GNVNAIHTFSDFN------DNNAYSCMRAEAEHIFLSETVFRNACLEMSHAAILG-RARGTNIDID-------QISNDMLNIAVEYVIAELPFFIGGAEILGTQEA--VLIYHKPWEL-----GEQIVRNDFSIRMK---------PNQGYLMVQEMENLS|
StAA4_010100031806_Ssp._256671793    EHACVGVSL-FNG-YFTAD----RLTRLVAWTAEHFP----------EYHFFVP-DEVAVYTLE-ALGYPAAR----------------------------------AKQKVHRQSCHVHNKIASALRVLG-VPDPERRILGIARLR------TMPDYRELLREAETQLESDPAFRAAAYDASSWVLQG-KTGV-------------APDSEALRLAVRYLLAEIPLFAGSGLITGNPRS--MFVYHQRVPF-----LRRFFDREFAFRPR---------VGQGFLVVRDSVFAA|
NEMVEDRAFT_v1g241260_Nvec_156392580  GVVLFGMSP-GNP-YYKQP----IIEQYVEFLGKEPR----------KIVVFVP-QQPSVHTYR-AMG--SKD----------------------------------AVKRAKKHADYLRAHCKRAIKKLS-KLRELPGDFHFVDWP--REVATHVVYQEKLEEMTRLYHTHALFRQDAVREVARVLGK-TPRDEMSDKESadfypdLYNDDSIREAVLYIIEEFAYILASKALYDADEV--TFIYHRSWPI-----LEKLINGEYDGKPR---------QGYGFCIIC------|
NEMVEDRAFT_v1g241259_Nvec_156392578  GVVLFGMSP-GNP-YYKQP----IIEQYVEFLGKEPR----------KIAVFVP-QQPSVHTYR-AMG--SKD----------------------------------AVKRAKKHADYLRAHCKRAIKKLS-KLRELPGDFHFVDWP--REVATHVVYQEKLDEMTRLYHTHALFRQDAVREVARVLGK-IPRDKMSDKESadfypdVYNDDSIREAVLYIIEEFAYILASKALYDADEV--TFIYHRSWPM-----LEKLINGEYDGKPR---------QGYGFCIIC------|
LLB_2968_Llon_270159475              TKDLVLLSIRGNE-YCTGE----YLGAIVQQAVATHQtpidhsgakgKATFLIA-DEIYWHNIK-QMISSESEveilkqeaikigadyfltnlgafltpldmtieqfNEKYPEKSVDEIITIINQLADEQG----KNFEIVRWKTWV------AQNDSQEKINKMMGFYDSVEGLKESVIRTQNNFVKR-HGKDGD-------------EELWKLRSHDYLIEESPAVM--WLAASLGYN--FIVYPGEILPPfe--ATKEFFVVPNHVPR---------ISQGKNIIEECEHNE|
PFL_1389_Pflu_70728769               KRVVLAVSV-GQE-YHEDQ----KLRSTIHLINQSGFs---------HVKVVVA-DTLQRHNKH---GKSPGE----------------------------------ALSAAIRDGDAWLARNQSILDGL----RVPYHITRWNQEL------ASDRYAELRQQLNQVYQQREELRDAIDSTI-GVFTE-RLRLRDEH---------ADIERAAAQCREYILEEIPIIL--PLWADEGYH--YVLYPQQMTTAmatsRELLIEPHSPDRVR----------WLPLKFKKRGIPIP|
Bcep18194_C7320_Bsp._78060433        KTALLLISV-GKE-YHEGE----KLAATVDKINVAGFg---------QCVIGVA-DTLQRHNYV---TGSAHS----------------------------------NYQKSRDRGDAWLRDHSDTLAKL----SMRREILRWDDLL------RRDDYSQYYHLITNEYYSNHEYRHAINDTI-GVFAE-RNGLVQGT---------PEHEAAFYRSLFYILEECPIIM--PMWAQDGVD--FIIYPKQMTAAmsktREIFVQQGSDTRAN----------WLAVKFKKKSQSNS|
DaesDRAFT_0176_Daes_280956168        WNPYLGISI-NNR-AFTCE----YLHSYMKWAACRANtrfa------VVIVDII-QHINNQVFD---RSKPLS----------------------------------AIEKAFRKADEIRLLCEQAKSKLSTEEANRLVILEWTDLV------VDECFAHNLKIFREEYANNEKFKNAIVSITKNNLGT-IVKRL--------------NDIELEMLGQYLVNELPELATGFNYDGVHYN--LNVYPGNIASI----YAELLKMDFFHRIL-----------SKIRMIGEIASVE|
RICGR_1349_Rgry_160871669            KNLIYLVSV-GKE-KFENK----WVERFVNFVKEVKPq---------KVLIVVA-DSLQRFNIE---VDDNID-------------------------------pkeAFTKSIKQGEKWVMNYKPLFSSL----TINYEFVHWEALK------EDKDFEHYFHEIKKLDEEDNNFKEAL-EISSKEYTH-RPSRVIG----------VAYQKAEENSRQFLIEECAVF---HVLAKDKENL-AIVYPGAVT-------NILHYAIKHINEN------nrtKEHAFHWLDLRPTKT|
RICGR_0139_Rgry_160871694            ASLIIGVSV-GSK-KHDGE----ALAALVEAINRLHKsvrit-----NCTIAVC-DSLQRHNYR-IDGKTDEE---------------------------------kALSMSKKAGAEWIEANIETLKHL----NVDYTIVRWDTWL-----KDEKRYREAFLEISNLLAQDYAFQKTMDNSI-QVFSS-RFNKRYQELGIsap---INDDVLQKSCRDYLLEECAIIMKMWPLYKQEHSQ-YILYPEKMTEA----LEYVYKQVVSQKNL--------fKWVNFRFKKIMQLKG|
RICGR_1344_Rgry_160871535            QCLLMGISM-NQT-HQSGEE---LYAFINEIKKYTNIk---------KVIFVIT-DYLHRHYVQLETGLPLEE----------------------------------AGKEAEKMGESWLQLNEASLNSL---SPVELQLVQWKSLVEGSNQIEDTSYSDCLSKTENCYRDDP-FFQQMVDTYSNEFGQ-KHCNRLKNRIE------ITLEACQQAAKNYFLEESTIIL---KFISLNFD--VITYPGKCNQ-----GINYIYNKYIGKPL---------NFISYRFRSEHVKNS|
plu0297_Plum_37524314                DHALIGISP-FNS-RFSKD----YVVDLIQWSSHYFR----------QVDILLPCEREASRLLV-ASGIDNVK----------------------------------AIKKTHREIRRHLRNLDYVISTAT-LKSKQIRVIQFSDFS------LNHDYQSLKTQVENAFNESESFKKSCLDMSFQAIKG-RLKGTGQYFG-------QIDLQLVYKALPYIFAEIPFYLNTPRLLGVKYS--TLLYHRPWSI-----GKGLFNGSYPIQVA---------DKQSYGIVTQL----|
SJA_C1-32620_Sjap_294013248          GHALIAVSL-FNS-YYSTD----RITTLVRWAKHTFE----------RSHIPVF-DLPHAYTLS-ARKGHSPG----------------------------------SVRRARKEGRKLHNKIARIHAEEG-IDSDAHRLLTWDDLV------RNDDYLQLRVDVDRAFCTDACFRQACLTMADTLLDP-EITDE------------DDRRAACFLAARYLLDELPLLIDLPSIIKCDSS--VFLYHRAPSL-----LKQLFEGRFTLKPS---------DRQGYFILSDASEIE|
jk0923_Cjei_68536000                 EHLIVGVSP-FNP-RFTPE----WLSSAFQWGAERFN----------TVDVLHP-GEISMSLLT-STGTPLGR----------------------------------AKRKVRQQCNRDMRNVEHALEISG-IKLGRGKPVLISDYL------QTQSYQCRRRSVIAEFQNNQIFQDACRAMSRAACQS-RLRVTN-----------VNIEPDIETAVKYIFDELPAYTHCSDLFEYETA--ALGYPTEWPI-----GKLIESGLTSLERD---------PNSSFIVIDFEKELI|
FG04615.1_Gzea_46117546              GHCAVAICL-GET-PLDDP---SNIYDLIMSLQQRFS----------DIVFLIC-DEIHKYEMMIPRNMTITR----------------------------------AQRLAIKKGDNMDAILNNVFEHLR-QADQLLANLTILRWS-------QIEDQDYQKVHDIMYQYRNQFEQELSTSSGFYIKR-RLAVA------------TLTEERLENFTKYTLAELPV-----QLLGFNHN--NRQYTTIFHP------VYPRKNADGSAGD---------VNSAYVSPIETVVNA|
pc1814_CPro_46447448                 KPVIILVST-HSS-FHEKISGDLKMNAFVSTIRNHVKg---------KITVLLS-DRAHINTMSLRFQNDLQK----------------------------------AQEECLIKAHALRNRYQSYFEN--------CNVVYGHSYI-----SQNKNFASFLKVIESLAENDSTFHELLLKDAESAYSN-TFIHLF-----------PDKNLFIKNTREDILTQCASSL---VLIDKGYR--YQFYPGSSYES-----LDYLNRIFISQEK----------QLSWIRVFLTIEKK|
pah_c026o110_Paca_282890774          APVILPISI-HSN-FHEGFQGDFKMNAFISTIKKHVKg---------KITVLLT-EKAHVKTLSLKYSNNFER----------------------------------AFEECLKSAHMLAQRYESIFEN--------CNVVYWHSYI-----CQDPYFKKIVELLEDLYSSDLNFQKLLHEDAEISYENLHHRESY-----------MNKSLYIEKCIEDLIEQCACLL---VLVNKGYK--FQFYPGKPCLS-----TEYVNRIFVPKDN----------RISWIDVFLSIEKK/
PH1011_Phor_14590851_(PDB:2cyc)      LQHYIGFEI-SGY-IHLGT----GLMAGAKIADFQKAg--------iKTRVFLA-DWHSWINDK-LGG---------------------------------------DLEVIQEVALKYFKVGMEKSIEVMGGDPKKVEFVLASEIL------EKGDYWQTVIDISKNVTLSRVMRSITIMGRQMG---------------------EAIDFAKLIYPMMQVADIFYQG-------------VTIAHAGMDQR----KAHVIAIEVAQKLRyhpivhegeKLKPVAVHHHLLLGL\YTRS
tyrS_Aper_74577141_(PDB:2cya)        IKGYIGYEP-SGV-AHIGW-----LVWMYKVKDLVEAg--------vDFSVLEA-TWHAYINDK-LGG---------------------------------------DMDLIRAAARIVRRVMEAAG-----VPVERVRFVDAEELA------SDKDYWGLVIRVAKRASLARVRRALTIMGRRAEE--------------------AEVDASKLIYPLMQVSDIFYMD-------------LDIALGGMDQR----KAHMLARDVAEKLGr---------KKPVAIHTPIISSL|
TYS1_Scer_6321624_(PDB:2dlc)         LKLYWGTAP-TGR-PHCGY-----FVPMTKLADFLKAg--------cEVTVLLA-DLHAFL-DN-MKA---------------------------------------PLEVVNYRAKYYELTIKAILRSIN-VPIEKLKFVVGSSYQ------LTPDYTMDIFRLSNIVSQNDAKRAGADVVKQV----------------------ANPLLSGLIYPLMQALDEQFLD-------------VDCQFGGVDQR----KIFVLAEENLPSLGy---------KKRAHLMNPMVPGL|
tyrS_Bsub_16080019                   IRLYSGFDP-TADSLHIGH-----LLPILTLRRFQLAg--------hHPIALVG-GATGLIGDP-SGKKAERTl---------------------------------NTADIVSEWSQKIKNQLSRFLDFE-AAENPAVIANNFDWI------GKMNVIDFLRDVGKNFGINYMLAKDTVSSR-IE---------------------SGISYTEFSYMILQSYDFLNLY---RDKN------CKLQIGGSDQW----GNITAGLELIRKSEe-------eGAKAFGLTIPLVTKA|
ECs2346_Ecol_15831600                IALYCGFDP-TADSLHLGH-----LVPLLCLKRFQQAg--------hKPVALVG-GATGLIGDP-SFKAAERKl---------------------------------NTEETVQEWVDKIRKQVAPFLDFD-CGENSAIAANNYDWF------GNMNVLTFLRDIGKHFSVNQMINKEAVKQRLNRED-------------------QGISFTEFSYNLLQGYDFACLN---KQYG------VVLQIGGSDQW----GNITSGIDLTRRLHq---------NQVFGLTVPLITKA/
trpS_Bsub_16078207                   QTIFSGIQP-SGS-VTLGN----YIGAMKQFVELQHD---------yNSYFCIV-DQHAITVPQ-------------------------------------------DRLELRKNIRNLAALYLAVG-----LDPEKATLFIQSEVP------AHAQAGWMMQCVAYIGELERMTQFKDKSKGN-----------------------EAVVSGLLTYPPLMAADILLYG-------------TDLVPVGEDQK----QHLELTRNLAERFNkk---yndiFTIPEVKIPKVGARI\WTRS
trpS_Ecol_16131262                   PIVFSGAQP-SGE-LTIGN----YMGALRQWVNMQDD---------yHCIYCIV-DQHAITVRQ-------------------------------------------DAQKLRKATLDTLALYLACG-----IDPEKSTIFVQSHVP------EHAQLGWALNCYTYFGELSRMTQFKDKSARYA----------------------ENINAGLFDYPVLMAADILLYQ-------------TNLVPVGEDQK----QHLELSRDIAQRFNal---ygeiFKVPEPFIPKSGARV|
TrpS_Gste_16974813_(PDB:1i6m)        KTIFSGIQP-SGV-ITIGN----YIGALRQFVELQHE---------yNCYFCIV-DQHAITVWQ-------------------------------------------DPHELRQNIRRLAALYLAVG-----IDPTQATLFIQSEVP------AHAQAAWMLQCIVYIGELERMTQFKEKSAGK-----------------------EAVSAGLLTYPPLMAADILLYN-------------TDIVPVGEDQK----QHIELTRDLAERFNkr---ygelFTIPEARIPKVGARI|
trpS_Aper_118431880_(PDB:3a04)       VAVLTGFMP-SGK-FHFGH----KL-TVDQLIYLQKNg--------fKVFVAIA-DAEAFAVRR-IG----------------------------------------REEAVRIAVEEYIANMIALG-----LDPKDTEFYFQTNRGT-----PYFRLIQLFSGKVTAAEMEAIY--------------------------------GELTPAKMMASLTQAADILHVQ--LDEYGGYR---HVVVPVGADQD----PHLRLTRDLADRMA-----gvveLERPASTYHKLQPGL|
WRS1_Scer_6324475_(PDB:2ip1)         FFLYTGRGPSSDS-MHLGH----MI-PFVFTKWLQEVfd-------vPLVIELT-DDEKFLFKH-KL----------------------------------------TINDVKNFARENAKDIIAVG-----FDPKNTFIFSDLQYMGG----AFYETVVRVSRQITGSTAKAVFGFN-----------------------------DSDCIGKFHFASIQIATAFPSS--FPNVLGLPDKTPCLIPCAIDQD----PYFRVCRDVADKLK---------YSKPALLHSRFFPAL/
consensus/95%                        ...hh.hS...p....p.......h.........................h.h.........h.........p..................................s.p.s....p.........................h.ph........................pp..h.....p.....h.................................hh.b.s.......h...........Y.....................................s...........
consensus/90%                        ..hhh.lS...p....p.......h......................ph.h.hs........h.........p..................................s.pbs.p..p.........h...............h.ph...........a............pp..h...h.p.....h...p.........................s..alh.Ehshh.....hh..........Y..............h.......p..............s...........
consensus/85%                        .phlhslS...pp.bap......bh..hh.b...p............ph.hhhs......p.h....s....c..................................A.pbs.cp.p.....hpp.h............h..hpph........p..a.p.b..h.p.h.pp..hb.sh.p....hh...p..................p...b..s..Ylh.Ehshhh....hh....s..h.hY...h..........h.......p..............ua...p......
consensus/80%                        .phlhulS...sp.bap......bl..hh.h..pp.p..........ph.hhls.sp...p.h....s....c..................................A.pcs.+p.p..b..hpp.hp...........l..hpph........p..app.b.ph.p.h.pp..hbpsh.p.s..hh...c..................p...b..s.pYll.Ehshhh....lh....s..h.hY...h..........h.....s.p.............bua.hlp......
consensus/75%                        pphllulSs..ss.bap.p....bl.phlphh.pp.p..........plsllls.sp...p.hp...u.s..c..................................Abccsb+psp..b..hppshp...........l..hsph........p.sYpphb.phpp.h.pc..a+psh.c.s..hh...+..................pp..hp.s.pYll.EhPhhh....lhs...s..hbhY...h.b......pblhp...s.+.p...........bua.hlp......
consensus/70%                        pphllGlSs..ss.bas.p....bl.phlpah.pphp..........plsllls.sp...p.hp...G.s..c..................................Abc+sb+psp..bpphppshpp.s........l.phsch.......pp.sYpphb.phpp.a.sc..F+psh.c.sp.hl...+..................pp..hp.u.pYll.EhPhhh....lhs...s..hhhY...h.h......pblhp...s.+.p...........buahhlp......
consensus/100%                       ...hh.hs...p....p.................................h..........................................................p................................................................h...........h....................................b.s.......h...........Y.................................................
```

---


4. Phylogenetic tree of archaeal and bacterial methionyl-tRNA synthetases and paralogs.

  
  
For bootstrap values of internal nodes, download the Newick formatted tree file.
  
Species abbreviations are provided in the table at the end of this page.
  

  

  


---


5. Alignment of the archaeal and bacterial methionyl-tRNA synthetases and paralogs
  

```
metG_Mpul_15828961               5  IYITTPIYYPSGDLHLGHIY-STNIAWVLRNYKKIQ---GYETFFSTGSDEHGQKIFNKAQELKLETQDYVDRQANK-FIDFW-KKANIDYDFF-ART-TNKE----HKE----VVREIFHKL--KEKNIIYLDKYVGLYSV-SDEEFLTETQALKK-DN----KFF--H-PVSNHELI----------------------------------KIE-EESYFFNLNL-------------------FIDW-IKEFL----D------QDIISSKAIVNELKSNFI------------------------------------------------------------NKGLENLSVTR---IKLDW-GIKID--------------------------------------QSSKHVIYVWLDALFQYLTNLG----Y--GSK-------NQSLYEKFW--------------------------KNGDERVHVV-GKEITRF-HCIYWPIFLKSL-----------------NVKMPTK----IISHGW-IVT-PEGK-MSKSKG---NVVDPVV-LIEK--------------------Y--GSEVLKYFLIA-KLSIKKD-GVFSEELLVSAYNNDLVNTFSNLISRTV   366
metS_Mgen_12044871               4  CYITTPIYYASGKPHIGHAF-TTILADVIKRFKIQN---GYEAFLLVGSDEHGNKIESKAKSLNLDPKTFVDINAQA-FKLMW-KTLNISFDHF-IRT-TDEI----HKQ----QVQKTFQDL--YDKKLIYQSEWKGAYCVECEQNYFTFNKQTML--------------CEIGHNLS----------------------------------LVQ-EPCWFISFSS-------------------TKNW-IETTI----GK-N---QLNIIPKSRASELKNNFI------------------------------------------------------------NNGLNDLALTR---KNVTW-GIKVP--------------------------------------FDPNQTIYVWFDALFSYITNLG----F---RN-------GDPNFIKWW-----------------------NNDNKEREVIHLI-SREITRF-HCIYWPIFLHLL-----------------DIKLPTQ----FLSHGW-IVDGEGRK-MSKSLN---NVISPEQ-LIDQ--------------------F--GVDGTRYCLLK-EMRLDKD-NRCSVSILKEIYNADLANSFGNHVSRTF   365
metS_Bsub_16077106               8  FYITTPIYYPSGKLHIGHAY-TTVAGDAMARYKRLK---GFDVRYLTGTDEHGQKIQQKAEQENITPQEYVDRAAAD-IQKLW-KQLEISNDDF-IRT-TEKR----HKV----VIEKVFQKL--LDNGDIYLDEYEGWYSI-PDETFYTETQLVDIERNEKG-EVIGGKSPDSGHPVE----------------------------------LIK-EESYFFRMGK-------------------YADR-LLKYY----EE-N---PTFIQPESRKNEMINNFI------------------------------------------------------------KPGLEDLAVSR---TTFDW-GVKVP--------------------------------------ENPKHVVYVWIDALFNYLTALG----Y--DTE-------NDELYQKYW----------------------------PAD-VHLV-GKEIVRF-HTIYWPIMLMAL-----------------DLPLPKQ----VFAHGW-LLM-KDGK-MSKSKG---NVVDPVT-LIER--------------------Y--GLDELRYYLLR-EVPFGSD-GVFTPEGFVERINYDLANDLGNLLNRTV   375
metS_Linn_16799293               8  FYITTPIYYPSGKAHIGHAY-TTVAGDAMARYKRLK---GYDVFYLTGTDEHGQKIQAKAKERGISEQEYVDEIAEG-FQELW-KKLEISNTDF-IRT-TQDR----HKT----SVEKIFEQL--LEQGDIYLGEYEGWYSV-SDEEYFTETQLEEVYKDENG-KVIGGKAPS-GNEVE----------------------------------LVK-EESYFFRMSK-------------------YADR-LVEYY----NS-H---PEFILPESRKNEMINNFI------------------------------------------------------------KPGLEDLAVSR---TTFDW-GIKVP--------------------------------------GNPKHVVYVWIDALSNYITALG----Y--NTD-------NDTKFQKYW----------------------------PAD-VQIV-GKEIVRF-HTIYWPIMLMAL-----------------DLPLPKM----VFGHGW-ILM-KDGK-MSKSKG---NVVDPYM-LIDR--------------------Y--GLDALRYYLLR-EVPFGSD-GLFTPEDFVDRVNYDLANDLGNLLNRTV   374
metS_Lpla_28377354               8  YYITTPIYYPSGKLHIGNSY-TTIACDTLARYKRAM---GYDVYFLTGTDEHGLKIEEKAEKLNTDPKSYVDGMAKQ-IKDLW-QLLEISNDKF-IRT-TDDY----HER----AVQEIFDRL--LKNGDIYLGEYEGWYSV-DDEEYFTETQLAEVFRDDNG-KVIGGKAPS-GHEVE----------------------------------LVK-EQSYFFKMSK-------------------YADW-LLDYY----QS-H---PDFIEPANRMTEMINNFI------------------------------------------------------------KPGLEDLAVSR---TSFTW-GVPVK--------------------------------------SDPKHVVYVWIDALTNYITALG----YA-TGD-------SEDLFNKFW----------------------------PAD-VQMV-GKEIVRF-HTIYWPIILHAL---------------G--LPLPKK----VFGHGW-LLM-KDGK-MSKSKG---NVIYPET-LVER--------------------Y--GLDALRYYLVK-AMPYGND-GLFTPEDFVARVNYDLANDLGNLLNRTI   375
metG_Smut_24380018               7  FYITTPIYYPSGKLHIGSAY-TTIACDVLARYKRMM---NHDVFYLTGLDEHGQKIQQKSEEAGITPQAYVDGMAVG-VKELW-KLLDISYDKF-IRT-TDDY----HEK----VVADVFEKL--LAQGDIYLGEYSGWYSV-SDEEFFTESQLEEIFRDEDG-KVIGGIAPS-GHEVE----------------------------------WVS-EESYFLRLSN-------------------YADR-LVDFF----HA-H---PDFIQPDGRMNEIIKNFI------------------------------------------------------------EPGLEDLAVSR---TSFTW-GVKVP--------------------------------------SNPKHVVYVWIDALLNYATALG----YG--QD-------AHANFDKFW----------------------------NGTVFHMV-GKDILRF-HSIYWPILLMML-----------------DLKMPER----LIAHGW-FVM-KDGK-MSKSKG---NVVYPEM-LVER--------------------Y--GLDPLRYYLMR-SLPVGSD-GTFTPEDYVGRINYELANDLGNLLNRTV   374
metG_Mlep_15827034               4  YYITTAIAYPNAAPHIGHAY-EYIATDAIARFKRLD---GLDVRFLTGTDEHGLKVAQAAEAAGVPTAQLARRNSGV-FQRMQ-EALHISFDRF-IRT-TDAD----HYK----AAKEIWRRM--DAAGDIYLGTYSGWYSV-RDERFFVDSE-TKLL-D-NGIRVA----VETGTLVT----------------------------------WTEKEQTYFFRLSA-------------------YVDK-LLAHY----DA-N---PDFIGPEVRRNEVI-SFV------------------------------------------------------------SGGLEDFSISR---TSFDW-GVQVP--------------------------------------EHPDHVMYVWIDALTNYLTGAG----FP--DT-------DSELFGRYW----------------------------PAN-LHMI-GKDIIRF-HAVYWPAFLMSA---------------G--IELPRR----IFAHGF-LHN-HGEK-MSKSVG---NIVDPMA-LVQT--------------------F--GVDQVRYFLLR-EIPFGQD-GNYSEEAIITRMNTDLANEFGNLAQRSL   365
Lebu_0685_Lbuc_257125470         5  FYITTPIYYPNAAPHVGTAY-TTIICDVVARYKRLA---GEEVGFMTGVDEHGQKIQEAAEKNGFTPQQWVDKMSLN-FTTLW-GKLNISNTDF-LRT-TQER----HLK----TVREIIKKV--HDKGDIYRGEYVGKYSV-SEETFVPENQL---V-D--G-KYM-------GKEVI----------------------------------DVK-ETSYFFKLSK-------------------YENA-LLEHI----EK-N---PDFIKPEGKKNEVI-AFI------------------------------------------------------------KQGLQDLSISR---TTFDW-GIPLE--------------------------------------LEEGHIIYVWFDALNIYLTGAG----FS---T-------DTEKFDKFW---------------------------TNGTVNHVV-GKDILRF-HAIIWPAMLMSA---------------G--IKLPDT----IAAHGW-WTV-EGEK-MSKSLG---NVVNPEE-EVEK--------------------Y--GLDAFRYYLMR-EATFGQD-ADYSKKAMVQRINADLANDLGNLLNRTI   359
metG_Smel_15965354               7  FYITTAISYPNGKPHIGHAY-ELIATDAMARFQRLD---GREVFFLTGTDEHGQKMQQTAKKEGISPQELAARNSAE-FQNMA-RLLNASNDDF-IRT-TEQR----HHE----ASQAIWMRM--GEAGDLYKDSYAGWYSV-RDEAYYQENE-TELRGD--GVRYGPQ-----GTPVE----------------------------------WVE-EESYFFRLSA-------------------YQDK-LLKHY----EE-N---PDFIGPAERRNEVI-SFV------------------------------------------------------------KSGLKDLSVSR---TTFDW-GIKVP--------------------------------------NDPSHVMYVWVDALTNYVTATGC---LT---D-------PTGPRAKFW----------------------------PAN-IHVI-GKDIIRF-HAVYWPAFLMSA---------------G--LPLPKR----VFAHGF-LLN-KGEK-MSKSLG---NVVDPFN-LVEH--------------------F--GLDQIRYFFLR-EVSFGQD-GSYSEEGIATRINSDLANGIGNLASRSL   366
metG_Mlot_13470650               6  YYLTTPIFYPNGKPHIGHAY-TVIATDALARFQRLD---GKDVFFLTGTDEHGLKMQQTAEKEGITPQALADRNSAI-FRSMT-EAVGGSNDEY-IRT-TEPR----HYA----SCQAIWKAM--AANGDIYLDRYSGWYSV-RQEAYFDESE-TTLGED--GVRREP---L--GSPVE----------------------------------WNE-EESYFFRLSA-------------------YQDK-LLALY----ES-Q---PDFVGPAERRNEVM-SFV------------------------------------------------------------KSGLKDLSISR---TTFKW-GVPVP--------------------------------------GDDKHVMYVWVDALTNYITAAG----YP---D-------TKSDQWRYW----------------------------PAT--HII-GKDIVRF-HAVYWPAFLMSA---------------G--IELPKR----VFAHGF-LFN-RGEK-MSKSVG---NVVDPFA-LIEH--------------------Y--GLDQVRYFFLR-EVPFGQD-GSYSHDAIVNRTNADLANGLGNLAQRSL   363
metG_Minf_189218093              1  FFITTAIDYVNGSPHLGHAY-EKILADAIARYYRNR---GYAVFFLTGVDEHGQKVQQSAEKEKMEVKKFCDIQAEK-FLQLW-EKLQIHYDAF-ART-TQPS----HVQ----YVRHALQKL--ADKGLIYFKEHEGYYSL-RQEQFVTEKDLVD------G-KWPE---IY-G-EVI----------------------------------KTK-EPNYFFRLSA-------------------FSEW-LKSYV----RD-N---ENWFIPQSKRKELL-GAL------------------------------------------------------------ERPLADLCISR-PVSRLSW-GIPLP--------------------------------------FDENYVTYVWFDALLNYVSFAR-----------------KEGDNQGWW----------------------------PAQ-LHVI-GKDIFIPAHAIYWPIILKAL-----------------ELEQPRH----FLIHGW-WMN-KGAK-MSKSLG---NFIDPLP-YLEI--------------------F--GADALRYYLLR-EMAFGQD-ADFADDKIWARYNSDLCNDLGNLVQRIT   355
SPAC27E2.06c_Spom_19115319      21  YFLTTPIFYVNAAPHLGHLY-SLVLTDAIARFQNLKP--DVSVISSTGTDEHGLKVQTVAQTEGVSPLQLCDRNSKR-FADLA-VAANTKFTHF-IRT-TNPK----HQA----SVQEFWKTI--QKAGMISFERHEGWYCV-SDETFYPESAIQKVV-DPATKQEKRVS-METGKEVQ----------------------------------WSS-EMNYHFLLSK-------------------FQSR-LIEHY----NK-N---PNFVQPSIFHTQVL-EEL------------------------------------------------------------KTGISDLSISR-PKQRLSW-GIPVP--------------------------------------GNSQQTIYVWLDALINYISVIG----YPW-LN-------EKSSLSAGW----------------------------PAN-MHVI-GKDIIRF-HCIYWPAFLMAA---------------G--LPLPEK----ILVHSH-WTM-NKVK-MSKSLG---NVVDPFW-LIEK--------------------Y--GVDTIRYYLLK-RGRLTSD-SNFDIEELEKDEEHDLRRSLGVLLSRLQ   390
MARS2_Hsap_14318634             46  AYFTTPIFYVNAAPHIGHLY-SALLADALCRHRRLRGPSTAATRFSTGTDEHGLKIQQAAATAGLAPTELCDRVSEQ-FQQLF-QEAGISCTDF-IRT-TEAR----HRV----AVQHFWGVL--KSRGLLYKGVYEGWYCA-SDECFLPEAKVTQQP--GPSGDSFPVS-LESGHPVS----------------------------------WTK-EENYIFRLSQ-------------------FRKP-LQRWL----RG-N---PQAITPEPFHHVVL-QWL------------------------------------------------------------DEELPDLSVSR-RSSHLHW-GIPVP--------------------------------------GDDSQTIYVWLDALVNYLTVIG----Y------------PNAEFKSWW----------------------------PAT-SHII-GKDILKF-HAIYWPAFLLGA---------------G--MSPPQR----ICVHSH-WTV-CGQK-MSKSLG---NVVDPRT-CLNR--------------------Y--TVDGFRYFLLR-QGVPNWD-CDYYDEKVVKLLNSELADALGGLLNRCT   412
DR_1433_Drad_15806450           20  FFITAAIDYANGTPHIGHVY-EKILADAIARYQRLA---GRDVTFVMGTDEHGEKISKAAAKGGVTPQELVDDLSERAFQGLW-KKLGISYDFF-IRT-TSAK----HKK----YVQDVLQRV--YDAGDIYFAEYEGLYSV-GAERYVTEKELVE-G--PDGVRRFPG--DK--DPPE----------------------------------LRR-EANYFFNMQK-------------------YQPW-LLETL----QQ-N---PDLIQPAGYRNEVL-EML------------------------------------------------------------KEDIGPLSISR-PKARVPW-GIELP--------------------------------------WDTDHVTYVWFDALLSYLTPLV------------------SQGQDASM----------------------------SGKAWHVI-GKDILKP-HAVFWPTMLRAA---------------G--LPLYRR----LVVHSHILAE-DGRK-MGKSLG---NAIDPEE-LVAA--------------------W--PVDAIRYALLR-EASLGAD-SPFGEGVLVSRLNSDLANDLGNLLSRTV   380
TTC0931_Tthe_46199235            5  FYVTTPIYYVNAEPHLGHAY-TTVVADFLARWHRLD---GYRTFFLTGTDEHGETVYRAAQAAGEDPKAFVDRVSGR-FKRAW-DLLGIAYDDF-IRT-TEER----HKK----VVQLVLKKV--YEAGDIYYGEYEGLYCV-SCERFYTEKELVE------G--LCP---IH-GRPVE----------------------------------RRK-EGNYFFRMEK-------------------YRPW-LQEYI----QE-N---PDLIRPEGYRNEVL-AML------------------------------------------------------------AEPIGDLSISR-PKSRVPW-GIPLP--------------------------------------WDENHVTYVWFDALLNYVSALD----YP-----------EGEAYRTFW----------------------------P-HAWHLI-GKDILKP-HAVFWPTMLKAA---------------G--IPMYRH----LNVGGFLLGP-DGRK-MSKTLG---NVVDPFA-LLEK--------------------Y--GRDALRYYLLR-EIPYGQD-TPVSEEALRTRYEADLADDLGNLVQRTR   361
metG_Tyel_206889557              7  FYITTPIYYVNDIPHIGHAY-TTIAADILARYMRLK---GRKVFFLTGTDEHGQKVERAALQKGKTPKEHADIMVEN-FKTLW-KGLNISNDAF-IRT-TDEE----HKR----IVQEILQRL--YDKGEIVKRKYSGMYCT-PCERFWTEKDLVE------G--KCP---DC-GRDVE----------------------------------FIE-EENYFFLMSK-------------------YQQA-LIEHI----EK-N---PSYILPETRKNEVL-GFL-----------------------------------------------------------KNRTLGDLCISR-PKHRLEW-GIILP--------------------------------------FDENYTTYVWFDALVNYYSALK----Y------------LAPENVEWW----------------------------PPD-HHLI-GKDILTT-HAVYWSTMLMAL-----------------ELPLPRN----IFAHGW-WTV-KGKK-MSKSLG---NVVNPSE-VIKK--------------------Y--GVDAFRYFLFR-EVSFGLD-GDFSEEALIRRINNDLANDFGNLLNRFL   362
DKAM_0547_Dkam_218883858         1  --MTTPIYYPNAPPHIGHAY-TTVFADVLARFHRLT---GGKVFFLTGNDEHGLKIQRVAEKAGKHPKEYVDEMANL-YREYW-RILNISYDYF-IRT-TDPY----HEK----AVKEAFNYI--YGKGLIYKAKYSGMYCV-DCEKYYSPGEYTE--VE--GKPYCP---IH-NKPLE----------------------------------YLE-EETYYFKLSE-------------------FKDY-LLDVL----E--N---QDIVYPSQYAREVASKIR------------------------------------------------------------NEGLRDVSVAR-PVERVWW-GIPVP--------------------------------------FDDKYVIYVWFDALMNYLSGIG----YP---D-------DKEKNTLYW----------------------------SNV-HHII-GKDILWF-HTAVWFSMLKAL-----------------DMPPPRR----VIVHSY-LIS-KGLK-ISKSIG---NVISIEE-LVER--------------------Y-NGSDGVRYLLSR-IFNTDKD-SEVSFELLDSIYNTELADTFGNLVRRVG   360
Francci3_0491_Fsp._86739206     16  YFVTTAIPYVNGKPHVGHAL-ELIETDAYARHLRQR---GHEVRFQTGTDDNALKNVQAAEAEGITPAEYVERVATG-FVGLR-GPLGLSFDDF-IKTSTDPR----HRP----GVEKLWSAC--ADRGDFYRKTYAGLYCV-GCELFYQPDELVD------G--RCP---EH-GTVPD----------------------------------LVE-ESNWFFRLGR-------------------YQDQ-LHALI----ST-D---RLRIEPAARKREVL-SFI------------------------------------------------------------EAGLEDFSASRSMSRARGW-GIPVP--------------------------------------GDPEQVIYVWFDALGNYITAPG----YG---N-------DDDSFQYWW--------------------------NDADRRVHVI-GKGIIRF-HAVYWPAMLLSA---------------G--VRLPDT----IFVHEY-LTA-DSMK-ISKSAG---NAEDPAD-IVAA--------------------F--NTDALRWWMLR-DVARAGD-TDYTTERLVTRANEDLANNIGNLVNRTV   377
Dbac_3184_Dbac_256830947         4  FFISTPIYYVNARPHLGHGY-TTIVADSVSRFHRLK---GDATFFLTGTDEHGDKIVQAAEAAGQDPKTYSDTISQL-FKDLW-PVLEVSNDQF-IRT-TDLR----HKA----CVRHVLQTV--FDKGDIYFDEYGGHYCF-GCERFYTDKELVD------G--KCP---DH-QTVPT----------------------------------FIK-EKNYFFRMSK-------------------YLEP-LREYI----EA-N---PDFIQPERYRNEVL-SML------------------------------------------------------------KEDLGDLCISR-PKTRLTW-GIELP--------------------------------------FDSDFVTYVWFDALINYISALG----YP-----------DGEDFRKYW----------------------------SGA-HHLV-AKDILKP-HAIFWPTMLMSA-----------------EIPLYKG----LRVHGY-WTV-NETK-MSKSIG---NVVAPLD-MAQK--------------------Y--GLSAFRYFLLS-EMSFGQD-SSFSEDALVGRFNADLANDLGNLFSRSL   359
metG_Pmar_225850511              6  FYVTTPIYYVNDLPHLGHAY-TTVAADVLARYFRQK---GVKTFFLTGTDEHGLKIQKSAEEKGISPKELADITHKK-FKELW-EVLNISYDRF-IRT-TDPD----HIK----AVQYIFQKC--YDNGDIYLSEYESWYCV-GCEEFKTETEIKD--Y----DYKCP---VH-LKPCE----------------------------------KVK-EESYFFRLSK-------------------YQDK-LLKLY----QE-K---PDFIMPEYRKNEVV-SFV------------------------------------------------------------KQGLKDLSVSR-KRERVKW-GIPVP--------------------------------------FDPDHTIYVWFDALTNYLTAVG----Y------------PPSPNE-FW----------------------------PAD-VHIV-GKDILRF-HAVYWPAFLMSA---------------G--IEVPEM----VFAHGW-WTV-EGHK-MSKSLG---NVVDPFE-ASQK--------------------Y--GVDQLRYFLMR-EVPFGLD-GDFSKKAVIGRINSDLANDLGNLFSRSL   361
metG_Aae_15606482                7  FYVTTPIYYVNDVPHLGHAY-TTIAADTIARYYRLR---DYDVFFLTGTDEHGLKIQKKAEELGISPKELVDRNAER-FKKLW-EFLKIEYTKF-IRT-TDPY----HVK----FVQKVFEEC--YKRGDIYLGEYEGWYCV-GCEEFKSEAELAE-------DHTCP---IH-QKKCE----------------------------------YIK-EPSYFFRLSK-------------------YQDK-LLELY----EK-N---PEFIQPDYRRNEII-SFV------------------------------------------------------------KQGLKDLSVTR-PRSRVKW-GIPVP--------------------------------------FDPEHTIYVWFDALFNYISALE-------------------DKVEIYW----------------------------PAD-LHLV-GKDILRF-HTVYWPAFLMSL---------------G--YELPKK----VFAHGW-WTV-EGKK-MSKTLG---NVVDPYE-VVQE--------------------Y--GLDEVRYFLLR-EVPFGQD-GDFSKKAILNRINGELANEIGNLYSRVV   359
metG_Gsul_39997330               5  FYVTTPIYYVNDVPHIGHAY-TTLAADVLARYKKLK---GYEVFFLTGTDEHGQKVEKAANTAGETPLELADRVVKR-FQGLW-EKLDIQYTDF-IRT-TQER----HKK----GVSHIFTKI--MAQGDIYLGEYEDWYCT-PCETFWTETQLID------G--KCP---DC-NRPVD----------------------------------KLK-EESYFFRMSK-------------------YQEQ-LLAHI----EA-N---PDFIQPKSRRNEII-SFV------------------------------------------------------------KEGLRDLSVSR---TSFNW-GIPVP--------------------------------------GNEKHVIYVWFDALTNYITALG----YP--DE--------QSAFKTFW----------------------------PVD-VHLI-GKDILRF-HAVYWPTFLMAA---------------G--LPVPKK----VFAHGW-WTV-EGQK-MSKSLQ---NVVEPNM-LVDR--------------------Y--GIDAVRYFLLR-EVPFGLD-GDFSHAALVHRINSDLANDLGNLLNRST   359
metG_Pcar_77919292               5  FYVTTPIYYVNDVPHIGHAY-TTVACDMLARYKRAQ---GQEVFFLTGTDEHGQKVEKAAQAKGETPLELADRVVKR-YQALW-EKLNISHTDF-IRT-SQER----HKK----GVCDLFEKL--HATGDIYLGEYEDWYCT-PCETFWTETQLMD------G--CCP---DC-GRPTD----------------------------------KLK-EESYFFRMSK-------------------YQDQ-LLKHI----EE-N---PDFIQPRSRRNEIL-NFV------------------------------------------------------------REGLRDLSISR---TSFSW-GIPVP--------------------------------------GNERHVIYVWFDALTNYITALG----YP--DD-------PEGHYKNFW----------------------------PAN-VHII-GKDILRF-HTVYWPTFLLAA---------------G--LPLPAK----VFAHGW-WTV-EGQK-MSKSLQ---NVVEPNM-LVDK--------------------Y--GVDAIRYFLLR-EVPFGLD-GDFSHAALVHRINSDLANDLGNLVSRST   360
metG_Bhyo_225620506              6  FYITTPIYYPSDYLHIGHCY-CTIATDTMARYKKIM---GYDVYFLTGTDEHGEKIARKAEAAGTTPKAYVDNIVNA-TKELW-KRLHIDYSHY-IRT-TDDY----HER----RVQKIFKIL--YDKGYIYKGSYKGLYCV-SDEAFFTESQVVK--KD-DGKFYCP---DC-EKELE----------------------------------YKE-EECYYLKLSE-------------------YGQW-LIDYY----KE-H---PEFLEPKERQNEMLKNFL------------------------------------------------------------LPGLEDLAVSR---KGLQW-GIPCP--------------------------------------VDSEHSIYVWIDALANYITALG----YP--EEG------QDELYKKYW----------------------------PAD-VHFV-GKEIVRF-HAIIWPIMLKML-----------------DIPLPKK----VYGHGW-VLF-DDGKKMSKSSG---NVVDPNT-LIDK--------------------Y--GVDALRYFLMR-EINFGSD-GYYSQELFLKRINSDLANDYGNLWHRIT   369
metG_Cper_18311506               6  YYITTPIYYPSTNLHIGNTY-TTVAADAIARFKRLT---GHEVMFLTGTDEHGQKIERIANEKGITPKEHVDEIVAG-IKDLW-KMMNISYDKF-IRT-TDDY----HVK----AVQEIFKKL--YDQGDIYKDSYEGLYCT-PCESFWTETQLVN------G--NCP---DC-GRPVE----------------------------------KAK-EEAYFFKMSK-------------------YADR-LIQYI----EE-H---PDFIQPESRKNEMLNNFL------------------------------------------------------------RPGLQDLCVSR---TSFTW-GIPVS--------------------------------------FDEKHVIYVWIDALSNYITALG----YG--QE-------NQELYKKFW----------------------------PAD-VHLI-GKDILRF-HTIYWPIMLMAL---------------G--LELPKQ----VFGHGW-LLV-DGGK-MSKSKG---NVVDPVV-LVNM--------------------F--GADAVRYYLLR-EIPFGSD-GLFNNEIFIKKVNTDLANDLGNLLSRTI   362
metS_Cper_18311505               3  VIIGNAWPYANGSLHIGRVS-SWMAGDILARYHRAK---GDEVIFVSGSDCHGSPILNKAKEINKSPEELINKYHRE-FVRCF-NKLGFSFNIF-TRT-DSEY----HEK----QVKNIIKSL--YNKGFIYEKETEEFYCP-KCCDTLDEFGIKE------G--LCN---EC-NSKVE----------------------------------VRS-SNNLFFKLSY-------------------FQDY-IQNIL----DE-E----ESWRENAIKITK--RYL------------------------------------------------------------EGGLRDKILTR----EINW-GIEVPI------------------------------------EGFEDKRVYVWIDALLAYVTASK---KVI--EE-------KGQSLEEYW--------------------------NNEDSRIYLVHGKENIPF-HTTMFPAILSGI---------------G--LEKSEIR---IFSSQY-LTL-EG-KTFSTNRN--WAIWVPY--IMER--------------------Y--NIDSIRYYLIS-RGAEEKN-SDFTWRDFINANNNELVGEIGNFTNRVL   363
Acid_3353_CSol_116622455         3  YYLTTPLYYVNAAPHIGHTY-TTMAAETIARFKRMQ---GYDAVMFTGTDEHGQKVERSAEAAGKTPQEFTDTIARE-FQQQW-EKLNIRVDRT-IRT-TDPR----HHK----VVQRLFQMC--MDNGYIYKGSYSGQYCV-YDELYANDAK--------PG-DPCP---IC-GRITE----------------------------------TVT-EENYFFKLSA-------------------FTEK-LLELY----ES-Q---PDFIQPEARRNEVI-SFV------------------------------------------------------------KQGLNDLSISR---TTIKW-GIPLP--------------------------------------VEGNHVFYVWFDALIGYMSAVD---------------------GEDLW----------------------------PAD-LHLM-GKEITRF-HAVFWPAFLMAG---------------G--VPLPKR----IVAHGW-LLF-ENDK-MSKSRG---NIVRSEP-IRQV--------------------M--GADALRYFLLR-EIVFGQD-GSFSYDALIGRYNSDLANGLGNLASRTL   349
TM1085_Tmar_15643843             3  FYITTPIYYVNSEPHIGSAY-TTIVADIIARYKRFM---GYDVFFLTGTDEHGQKVLQAAQQAGKDPQEFCDELAEK-FKRLW-KELKITNDYF-IRT-TDEM----HMK----TVQEFVAKM--KENGDVYKGIYKGWYCV-PCETFWNEDEVIK---E-GEERFCP---EC-KRPVK----------------------------------WVE-EENYFFRLSK-------------------YRDS-LLKYY----EE-H---PDFVEPDFRRNEML-KIL------------------------------------------------------------EGGLKDLSITR---TTFKW-GVPMK--------------------------------------DDPEHVIYVWVDALINYISAIG----YGW----------NDEMFNKWW----------------------------PAD-LHLI-GKEINRF-HSIIWPAMLMSV---------------G--LPLPKK----VFAHGW-LTV-NGQK-ISKSLG---NAIDPRF-FVKR--------------------Y--GNDVVRYYLIR-DIMFGKD-GDFSEERLVHRLNSDLANDYGNLLHRIT   361
metG_Dthe_206900100              5  FYITTPIYYSNSEPHIGTVY-TTIVADTFARFYRLK---DYDVFFLTGLDEHGQKLARTAQEKGYMPQEYVDMMAQK-FLETW-KKIGITNDDF-IRT-TQKR----HEE----VVQKVFQKL--YDEGYLYKGSYAGWYCT-PCETFWQKEDLHD--------GNCP---SC-GRPVE----------------------------------WLE-EETYYFKLSA-------------------FAEP-LLKYI----EE-H---PEFVYPETRRNEVI-SFI------------------------------------------------------------KSGLKDISATR---TTVKW-GVPVP--------------------------------------FDPKHTVYVWFDALVNYISALG----YL--TN-------DDTKFKRYW----------------------------PAD-IHLI-GKDILRF-HAIIWPAILMAL-----------------NIPLPKT----VLAHGF-WTI-KGGK-ISKSKG---NRVDPHE-LINL--------------------Y--GVDALRYFLLR-EVPLGLD-GEYSDEAFHRRYHSDLANDLGNLVNRVL   360
metS_Abut_157737467              8  VYITTPIYYVNDVAHIGHAY-TTIIADMLARYSRLT---GSNTFLLTGTDEHGQKIAQSAEIRNKTPKEYADEISGK-FRTLW-DDFDITYDKF-IRT-TDEE----HKI----GVQKAFEIM--YKKGDIYKGEYEGFYCV-PCETFFPESQLVD-------EQFCP---DC-GRATT----------------------------------LVK-EESYFFELSK-------------------YEEK-LLKWY----EE-N---EDCILPRSKKNEIV-NFV------------------------------------------------------------KGGLKDLSISR---TSFSW-GVKLPESM-----------------------------------NEPKHVMYVWLDALLNYITALG----Y----G-------TDDKNMNFW----------------------------PAN-IHLV-GKDILRF-HSIYWPAFLMSL-----------------DLPLPKH----IAAHGW-WTR-DGEK-MSKSKG---NIVNPKE-VADA--------------------Y--GLDAFRYFMLR-EVPFGQD-GDFSQKALIDRINSDLGNDLGNLLNRIS   365
metG_Wsuc_34556660               5  SFITTPIYYVNDIPHIGHAY-TTMIGDSLAHYYRLI---GHETFFLTGTDEHGQKIEQSAQKKGKTPQAYADEISGA-FKSLW-DRFEISYDHF-IRT-TDAK----HKQ----GVQKAFLKM--FEKGDIYKGEYEGHYCV-SCETFFTKRQLVD-------DLYCP---EC-GKETS----------------------------------LVK-EESYFFALSR-------------------YQEK-LLAWY----KE-N---PDCILPKPKKNEVM-RFV------------------------------------------------------------ESGLDDLSITR---TSFDW-GVKLPQEM-----------------------------------NAPKHVMYVWLDALLNYVTALG----Y----G-------DDEKRMDFW----------------------------PAR-VHLV-GKDILRF-HAIYWPAFLMSL-----------------DLPLPKH----VAAHGW-WTR-NGAK-MSKSVG---NVINPQE-VADA--------------------Y--GVEPFRYFMLR-EVPFGQD-GDFSQKALIDRLNSDLSNDLGNLLNRLG   362
Pmob_1239_Pmob_160902697         4  FYVTTPIYYVNSEPHIGSAY-TTIVADIVARFKRMM---GYDVFFLTGTDEHGQKVLQAAKEKNIPPQEYVDSLSSK-FKNLW-DEMGISYDHF-VRT-TDAY----HVK----TVQMFVDKM--MENGDVYKGKYAGWYCI-HDESFWDESEIVT--QD--GVKLCP---EC-NRELK----------------------------------WVE-EENYFFKLSK-------------------YTEP-LLKHY----EN-N---PDFVEPSFRRNEML-QIL------------------------------------------------------------NDGLKDLSITR---TTFDW-GIPLK--------------------------------------SDPKHVVYVWVDALINYVSAIG----Y---SD-------DQQKFNRYW----------------------------PAD-LHLI-GKEINRF-HSLIWPAMLMSV---------------G--LPLPKK----VFAHGW-LTV-NGQK-ISKSLG---NAVDPRI-LMDA--------------------Y--GRDVIRYYLLR-DIAFGRD-GDFSEENLITRYNADLVNDLSNLVHRTL   362
metG_Hpyl_15612032               4  SLITTPIYYVNDIPHIGHAY-TTLIADTLKKYYTLQ---GEEVFFLTGTDEHGQKIEQSARLRNQSPKAYADSISAI-FKNQW-DFFNLDYDGF-IRT-TDSE----HQK----CVQNAFEIM--FEKGDIYKGTYSGYYCV-SCESYCAVSKVDN--TD--SKVLCP---DC-LRETT----------------------------------LLE-EESYFFKLSA-------------------YEKP-LLEFY----AK-N---PEAILPIYRKNEVT-SFI------------------------------------------------------------EQGLLDLSITR---TSFEW-GIPLPKK-----------------------------------MNDPKHVVYVWLDALLNYASALG----Y---LN-------GLDNKMAHF----------------------------E-RARHIV-GKDILRF-HAIYWPAFLMSL---------------N--LPLFKQ----LCVHGW-WTI-EGVK-MSKSLG---NVLDAQK-LAME--------------------Y--GIEELRYFLLR-EVPFGQD-GDFSKKALVERINANLNNDLGNLLNRLL   365
FN1268_Fnuc_19704603             5  FFVSTPIYYVNGDPHVGSAY-TTIAADVINRYNKAM---GMDTHFVTGLDEHGQKVEQAAKQNGFTPQAWTDKMTPN-FKNMW-AALDIKYDDF-IRT-TEDR----HKK----AVKRILDIV--NAKGDIYKGEYEGKYCV-SCETFFPENQLN-------GSNKCP---DC-GKDLT----------------------------------VLK-EESYFFKMSK-------------------YADA-LLKHI----DE-H---PDFILPHSRRNEVI-SFI------------------------------------------------------------KQGLQDLSISR---NTFSW-GIPID--------------------------------------FAPGHITYVWFDALTNYITSAG----F---EN-------DENKFDKFW---------------------------NNSRVVHLI-GKDIIRF-HAIIWPCMLLSA---------------G--IKLPDS----IVAHGW-WTS-EGEK-MSKSRG---NVVNPYD-EIKK--------------------Y--GVDAFRYYLLR-EANFGTD-GDYSTKGIIGRLNSDLANDLGNLLNRTL   362
metG1_Scel_162448397             5  FYVTTPIYYINDVPHLGTAY-TTIAADVLCRYHRLR---GHESRMLTGTDEHGLKIQRAAEARGVAPGAHADEIAAV-FRATW-PKLGCAPDDF-IRT-SEPR----HKK----AVQELWSRI--KARGDIYLGHYEGLYCV-GCEAYYTEKDLEQ------PGNVCP---LH-KKPAE----------------------------------SVK-EESYFFRLSR-------------------YGDA-LLDYY----KR-N---PTFVQPASRLNEVV-SFV------------------------------------------------------------REGLQDLSVSR---TTFEW-GIPVP--------------------------------------DDPKHVMYVWFDALANYMTALR-----------------EPEDNTRFW----------------------------PAD-VHLV-GKDILRF-HAVYWPAFLLSAG------------Y-G-EAELPRQ----VFAHGF-LTY-SGQK-MSKTLR---NTISPVE-VATALSEAAAAALPGGPGAAAGAPL-VGVDVVRYCLMR-AISFGQD-GDFSLQDVLSRYGSELGNALGNLLNRVL   382
SCO3792_Scoe_21222203           14  YYVSTPIYYVNDAPHLGHAY-TTVAGDVLTRWHRQR---GEKVWYLTGTDEHGQKIMRTAEANGVTPQAWADKLVTESWKPLW-EHLDIANDDF-IRT-TQKR----HTD----RVQEFVQDL--YDKGEIYKGGYEGPYCV-GCEEYKLPGELLDGEGEYAGQKLCP---IH-KKPVE----------------------------------ILS-EENYFFKLSE-------------------YSEK-LLAHY----EA-N---PGFVQPESARNEVV-NFV------------------------------------------------------------RQGLQDLSISR---STFDW-GVPVP--------------------------------------WDDKHVIYVWVDALLNYATAVG----Y---NE-------NPEKFESTF----------------------------PAD-VHLV-GKDILRF-HAIIWPAMLMAQ---------------G--LPLPGK----IAANGW-LMV-GGEK-MSKSNL---TGIKPQD-LTTH--------------------F--GVDAYRWYFLR-AIAFGQD-GSFSWEDFSARYTSELANDYGNLASRVA   377
metS_Maer_166367026             10  FALTTPLYYVNDIPHIGSAY-TTIVADVMARWQRLQ---GNSVLLITGTDEHGQKIERTAAAKGLNPQEHCDRIATS-FANLW-AKLHIQYDRF-SRT-TAPR----HQA----IVNEFFERV--WEKGDIYLDRQQGWYCV-ACEEFKEKRELLD-------NGCCP---IHTNLAAE----------------------------------WRD-EENYFFRLSK-------------------YQTQ-LEQFY----QE-Q---PDFIQPESRRNEVL-NFV------------------------------------------------------------NQGLQDFSISR---VNVAW-GFPIP--------------------------------------HDGQHTIYVWFDALLGYVTALL---DPEDEPT-------LENALAKWW----------------------------PID-LHLI-GKDILRF-HAIYWPAMLMSA---------------G--LPLPKR----VFGHGF-LTK-DGRK-MGKSLG---NTLDPFD-LVDR--------------------Y--GADAVRYYFVK-EIELGQD-GDFQETRFVNILNADLANDLGNLLNRTL   370
metG_Telo_22298292               5  FSLTTPLYYVNALPHIGSAY-TTIAADVLARFYRLQ---GYQVRFITGTDEHGQKIERTAQQRGLSPQAHCDEIAAG-FQALW-QQLNIHYDRF-SRT-TSPR----HHA----IVNEFFQRV--WDNGDIYLGQQQGWYCV-ECEEFKEERELLE-------GRRCP---IHVNRTVE----------------------------------WRD-ERNYFFRLSK-------------------YQQA-LLDHY----AE-H---PDFVQPPSRRNEVL-SFI------------------------------------------------------------ERGLQDFSISR---VNLAW-GFPVP--------------------------------------TDPEQTLYVWFDALLGYVTALL---EPEDEPT-------LANALKTWW----------------------------PIN-LHII-GKDILRF-HGISWPAMLMSA---------------G--LPLPEQ----IFVHGF-LTK-DGQK-MGKSLG---NTLDPFA-LVAQ--------------------Y--GADAVRYYFMK-EVEFGRD-GDFSETRFVTILNADLANDLGNLLNRTL   365
metG_Avar_75908935              11  FALTTPLYYVNDVPHIGSAY-TTMAADAVARFQKLL---GRDVLLITGTDEHGQKIQRSAESLGKAPQEFCDEIVPS-FMSLW-QLLNIQYDRF-SRT-TAVR----HKA----IVDEFFARV--WQAGDIYQGQQKGWYCV-SCEEFKEERELLE-------GNRCP---IHVNKEVE----------------------------------WRD-EQNYFFRLSK-------------------YQTQ-LEEFY----KS-H---PDFIQPESRRNEVL-NFV------------------------------------------------------------SQGLQDFSISR---VNLDW-GFPVP--------------------------------------TDSKHTLYVWFDALLAYVTALL---DPQDEPT-------LENALAKWW----------------------------PIN-LHLI-GKDILRF-HAVYWPAMLLSA---------------G--LPLPDR----VFGHGF-LTK-DGQK-MGKSLG---NTIDPVG-LVQQ--------------------Y--GSDAVRYYFLK-EIEFGKD-GDFNEVRFINVLNADLANDLGNLLNRTL   371
metG_Gvio_37522592               5  FALTTPLFYVNALPHIGSAY-PTLAADAVARYHRLR---GRSVRFVTGTDEYGLKIERQAAERHLTPREHCNEIAAG-FEKLW-RALSIDYDRF-IRT-TDPR----HAA----IVREFFERC--WQAGDIYKGRYTGLYCI-DCEEFKKKPDEDYF-IE-NGEPRCK---IHLKPLRE-----------------------------------QD-EENYIFALSR-------------------YQQK-LEQYF----DE-H---PEFVQPDFRAHEVR-NFI------------------------------------------------------------AGGLEDFSISR---ANVSW-GLPIP--------------------------------------VDSSQTIYVWFNALLGYVTALL---EPEDEPT-------LENALARWW----------------------------PID-LHIV-GKDILRF-HAVYWPAMLMSA---------------G--LPLPGM----IFGHGF-LTR-DGRK-MGKALG---NVIDPGV-LVEQ--------------------Y--GSDAVRYYFLK-AIEFGRD-NDFNETRFREILNADLANDLGNLLNRTG   369
OVA1_Atha_15233258              71  FVLTTPLYYVNAPPHMGSAY-TTIAADSIARFQRLL---GKKVIFITGTDEHGEKIATSAAANGRNPPEHCDLISQS-YRTLW-KDLDIAYDKF-IRT-TDPK----HEA----IVKEFYARV--FANGDIYRADYEGLYCV-NCEEYKDEKELLE-------NNCCP---VHQMP-CV----------------------------------ARK-EDNYFFALSK-------------------YQKP-LEDIL----AQ-N---PRFVQPSYRLNEVQ-SWI------------------------------------------------------------KSGLRDFSISR---ALVDW-GIPVP--------------------------------------DDDKQTIYVWFDALLGYISALT---EDNKQQN-------LETAVSFGW----------------------------PAS-LHLI-GKDILRF-HAVYWPAMLMSA---------------G--LELPKM----VFGHGF-LTK-DGMK-MGKSLG---NTLEPFE-LVQK--------------------F--GPDAVRYFFLR-EVEFGND-GDYSEDRFIKIVNAHLANTIGNLLNRTL   430
metG_Pmar_33861424               3  FVITTPLYYVNDKPHLGSIY-TTLICDSIARYKRLT---GADVIFITGVDEHGLKIQRTAKNKGVEPQFHCDEISDI-FKQNW-KNWDITHNKF-VRT-SSQK----HEY----IVKEFYNRV--KKSDDIYMGVQKGWYCV-GCEEFKDNPDNSP-------THKCP---IH-QKTLE----------------------------------WKN-EENLFFKLSK-------------------YQSQ-IEELI----K--E---PAFIQPKERRNEII-NFV------------------------------------------------------------SKGLKDFSISR---TNVSW-GISVP--------------------------------------DIDNHTFYVWFDALLGYVSAIS----L-DMKEPS-----LDESIDNGW----------------------------PAD-IHLI-GKDILRF-HAVYWPAMLISA---------------G--MKVPKK----VYGHGF-LTR-EGQK-MGKSLG---NVLDPDI-LLSK--------------------Y--GKEAVRWYLLK-DITLGQD-GDFQNKRFVDIINNDLANTIGNLLNRTS   361
metG_Gaur_226226140              4  FYLTTAIDYANGDPHLGHAL-EKIGADVIARYRRLC---GDDVHLLIGMDEHGQKVQQTAAKEGVAPQAFTDTIAAR-FQGMW-SRLGISYDQF-IRT-TEAH----HKL----GVQTLIRRIAERNPDDFYERSYTGMYCV-GCESFKQDADIVD--------GKCV---LHPTRTLE----------------------------------EVE-ERNWFFRLSK-------------------YQGF-LQDLL----AS-N---PSFIEPSSRRNEIL-GLL------------------------------------------------------------AQGLEDISASR---ARLDW-AVPFPLVL----------------------------------SNGETQGTYVWFDALPNYLTATG----F------------PDEGYDQRW----------------------------PAD-LHII-GKDITRF-HVVIWPAMLQAA---------------G--LPLPKQ----VWAHGF-VQL-GGER-FSKSAG---VKLDLGE-AIDR--------------------F--GVDAFRYVLLR-EVPFDGD-GNFSWERFEERYTADLANAFGNLASRAM   363
Amir_4208_Amir_256378249       129  ALVFCPPPTPNGGLHLGHLAGPYVRADVLVRALRST---GRDARHVTGTDDHQSHVAVSARLGGATPEQVAASHGEA-ILATL-RAAGVDCDRLTRPA-TAPG----HAD----RIRELITRL--AESPSVTEQERDTAYCA-ACDLSLHQAFAR---------GACA---HC-AADSD-GEICEA-CGRPNEAKDLVDPRCR--LCGGPAT-TRP-ERALWLDMSA-------------------HAEQ-LARYL-----------KDSHTSPDLLALVE-RLL------------------------------------------------------------ADGLPPYRLAR----RADW-GVELA----------------------------------------DGQRVDAWVDLALTFLDAAR----A-------------ET--EQDG----------------------------PAKITLFL-GYDNSFF-YAVLLPAVAFAA---------------GLAEHLPAA----FVTNQF-LHL-DDAK-FSTSRG---HAIWADP-ALAE--------------------A--GPDAVRLALLR-NAPEGRVTRITQERAALLAQDPLHLAAKEWLAGFAG   506
Sare_0570_Sare_159036236       132  VLLLPSFPTPNGELHLGHLAGPYLGADIARRALAAT---GTPVHLLLGTVGHQSQVAAAATAAQVSFHTLAEQNTDA-IMAGL-AAAGAEWDVF-VRP-TSPE----YPA----LARRVFERL--RDDGVVLTRTTPTHHCP-HCDRFLFEAFLA---------GACP---HCGSYDTA-GIECES-CALPFADTDLIDVTCA--TCGSPAE-RRP-LTRYFLPLEP-------------------LRDR-LTAYL-----------RTVRMGSRLAGYVD-RVL------------------------------------------------------------ANRLPDMLVSI----VADD-GIEVP--------------------------------------GTDGQRLYSAFELAARYLTAVD---RLA-RDQG-------ESGWESYL-------------------------TGRRPRTVLFF-GFDNAYL-RTIVFPAVLGAF---------------TDLAQLPDT----LVSNEF-YEL-DGAK-FSTGRR---HAIWARD-AFDD--------------------K--SRDRLRLYLAA-TRPEYRR-RSFSTAEYDLFVQHELVEGLDGWLGGIN   521
RSp1438_Rsol_17549657            4  YIVTITPPTPNGDLHLGHLSGPFLAADICRRLLRQA---GEDTILLSYSDDYQSYMPRKARQLRKETFGLARYNARQ-IELAM-QAAEIDIDCFLQAA-DSDT----FAR----FAGERFDDI--GRLGLLELKATPVFRCD-ACAVYGYEGLGR---------GHCN---WC-GASSD-ASQCEA-CARVPDVAHMQGMHCI--LCGGDMH-RVP-VTRYVWKIGA-------------------QYPA-IAEAL-----------KALPKRAALETYLA-DVL------------------------------------------------------------RNTSDAWPVTR----PGDA-GLELD--------------------------------------GYPDQPVNTWFMGLAGYQAALA---DYLAAHPE-------RGAFDDWWT--------------------------PDTQLVHFL-GYDCSYS-HAVGYTAQLLAR---------------PDGP-RPGV----YLTNQF-LKL-DG-QDFSTSRG---HAVWIRE-ITAQ--------------------H--PVDAIRLYTAL-CAPENET-RDFDRAAFEAWRKAIFDGIVAAYTRDLS   392
metG_Aful_11499048               2  KLVTCGLPYANGKAHVGHLR-TYVPADVYVRYLRMS---GEEVVFVCGSDCHGTPIVVNAEQQGLSPKELVDIYHEH-FIKIF-DALNIKFDFY-GRT-DSDY----HHH----RTTEIVKRL--IEKGYVYPKEIQLAYCP-KCQRFLPDRYVE---------GICP---YC-GALAR-GDECDQGCGRHLEPGEIKEPRCK--ICGSKAE-FRS-QRHYFFKLTE-------------------FQDF-LEDYL-----------SKLKGTENALNYAR-NWV------------------------------------------------------------KN-LRDWCITR----NLEW-GVRFP--------------------------------------GEPNLVVYVWVDAPIGYISFTE---KAC-EEK--------GCDWRKIWI-------------------------DGDAEIIHFI-GLDIVYH-HCIFWPAMLKGA-----------------DYALPSA----VVASGM-VKV-EG-KTFSKSRG---YVVWVEEDYLKSG-------------------L--SPDYLRYYIVN-YTSHQKD-LNFSWEVFREKVNNEVIATLGNFLYRVL   388
metG_Mjan_15669449               3  YLITTALAYTNGPLHLGHARSTYIPADIIYKYLKLR---GEDVIHVGGTDNHGVPITLTAEKEGKSPEEIVEKYHNE-IKEDL-DLLGVEFDAF-GKT-HSQI----HIE----TAQEFYLKL--KENGYIYEKEIEQFYCP-NCKKFLPDRYVE---------GICP---YC-GGEAR-GDHCEV-CGRHLEPFELKDPYCV--ICKGKPE-IRK-TKHHFFKLSA-------------------LKKE-LEEYI----KN-A---KEM--PEHVKNMAL-NWI------------------------------------------------------------KE-LHDWDISR----DISW-GVPIP---------------------------------------GTNQVMYVWLEAPIGYISFTK----ML------------GEIWKKYWL-------------------------EKDTKIYHFI-GKDITVH-HAVFWPGMLIAH---------------G-SFNLPTA----VVSGGY-LTL-EGRK-MSTSKR---WVVWVKD-FVKN--------------------F--DADYLRYYLIM-SAPLFKD-CDFSFDDFKNKINNELINIIGNFTHRVL   384
metS_Ssol_15897480               3  VLVTAAWPYVNSVPHLGNLIGSILSADVFARYARLRY-GKENVLFVSGSDEHGTPIEIEAIKRKVNPKELTDQAHEY-DRHLFLNVWKISFDNY-TRT-ESEI----HKK----FVREFLLKL--TK--YIKVSEDEIPYCE-NDKLYLPDRFVK---------GTCP---YC-GFEDARGDQCDN-CGKLLTPSLLVNPKCS--ICGKTPV-FKK-TKHWFFDLSE-------------------FNDK-IRGWI----SS-S---NEM--PDNVKSVAL-SWV------------------------------------------------------------GEGLKPRSITR----DNKW-GIPAPFE------------------------------------GAQDKSIYVWFEALLGYISAVI---EYF-ERKGD------QEKWKEYWF-------------------------GPNIKSYYFI-GKDNIPF-HAVILPAMLMAS---------------EEEYHLPDV----IAATEY-LLY-EGQK-FSKSRK---IGVWIDE-APEL--------------------M--DVEYWRFVLIR-LRPEEKD-TNFTWRETVRIVNTELNDDIGNYVNRVL   397
metG_Pfur_18977402               4  YMVTAALPYANGPIHAGHLAGAYLPADIFVRYLRLK---GEDVVFICGTDEHGTPISFRALNEKRSPREIVDEFHEH-IKTAF-QRVKISFDYF-GRT-ELPV----HYR----LSQEFFLKA--LENGYLVKKVTKQAYCE-HDKRFLPDRFVI---------GTCP---YC-GAENQRGDQCEV-CGRPLTPEILIEPRCA--FCKNPIT-FRE-STHYYIKMQE-------------------FEEK-LKEWI----K--E---KDW--KPNVKNMVL-GWI------------------------------------------------------------EEGLEERAITR----DLDW-GIPVPLD----------------------------------EEDMKNKVLYVWFEAPIGYISLTI---EYF-KRIGK------PNEWKKYWL-----------------------NLDGQTRVIHFI-GKDNIPF-HAIFWPAFLMAYGKYKDEE------V-EAEWNLPYD----IPANEY-LTL-EGKK-FSTSRN---WAIWIHE-FLDV--------------------F--PADYLRYYLTS-IMPETRD-SDFSFAEFKTKINEELVNVLGNFVYRAL   408
Nmar_0963_Nmar_161528471         5  AIITSALPYANGEIHLGHVASTYLPADVTTRFLKQN---GVEAYYVCASDDFGTPILIQSEKEGKTPAEYVAHWNKR-DYEDF-SAFDIDFDYF-YKT-SSDE----NIQ----FVQDVFKKL--NDAGHIYEQEIIQFYCN-NDKKFLPDRYVK---------GTCP---YC-KAEDQYSDLCES-CGR--VPEEITNPKCS--LCGQPPT-KEK-TTHYFFKLKN-------------------FGEP-LSKWL----D--E---NDHL-QKDVKKYVQ-NWI------------------------------------------------------------KSGLIDWDITR----DITW-GVPVPLD------------------------------------DAKDKVFYGWFDNHLAYISTAL---KFL---NDK------GIDGKEFWN---------------------------SADIYHFI-GKDIVYH-HYLFLPAMRLGI---------------ESEYKLPDY----IPTRGH-LTL-QAKK-ISKSRN---WYIGLKE-FLEY--------------------Y--PADYLRFYLVS-INPYSQDDLNFDWDDFTTRINSELIGNLGNFINRAL   393
Hbut_1110_Hbut_124027978         4  WIVASAWPYVNSIPHLGNLIGSILSADVFARYLRLK---GEDVVFVSGSDEHGTPIEVEAIRRGVHPKQLTDQAHEY-VKKLF-EEFRISFDNY-TRT-ENPV----HKE----FVREFMMKL--YQNGYIFEQDDVLPYCP-RDKMFLPDRFVV---------GTCP---YC-GFEKAYGDQCDN-CGRLLHPTELKNPRCS--ICGGPVE-FRK-SKHWFFNLPK-------------------LQEK-VEKWL----R--E---SNL--PPNVKNYSL-NML------------------------------------------------------------KEGLKPRSVTR----DNKW-GIPAPFP------------------------------------GAEGKTIYVWFDALLGYISATK---EYGL-KKGD------PELWKKYWF-------------------------NPETKTVYFI-GKDNIPF-HAIILPAMLIGS---------------GDPYVLPSY----ISATEY-LMY-EGEQ-FSKSRR---WGIWIDE-ALEI--------------------L--PADYWRFALIR-MRPEAKD-TNFTWSEFLRIVNTEMNDDIGNYVHRVL   396
metG_Aper_14601199               4  YVVTSAWPYVNHVPHLGTLIGSVLSADIYARYLRLR---GRQVVFVSGSDEHGTPIELEARKKGVHPKELTDQVHEY-DVKMW-REYRISFDNY-SRT-ESPV----HKE----FVMEFMKKL--EENGYIFSQEEVLPYCE-RDKIFLPDRFVE---------GTCP---YC-GYEKARGDQCDE-CGRLLHPTELKNPRCA--LCGSKPV-YKS-TRHWFIDLRR-------------------VQDR-LLKWL----ES-H---GEL--QDSVKKYSI-NWV------------------------------------------------------------AQGLKPRSVTR----DLSW-GVPAPFK------------------------------------GAEGKTIYVWFDALLGYVSATK---ELFIMRRGD------PEEWKSWWW-------------------------DSGTRTVYFI-GKDNIPF-HAIILPALFLAS---------------HDPYVLPWR----ISATEY-LMY-EGQQ-FSKSRR---IGVWIDE-ALEI--------------------A--PADYWRWALAR-MRPEARD-TNFTWKEFYRIVNTELNDDIGNFVNRVL   398
Kcr_0066_CKor_170289693          3  WIVASAWPYINAVPHLGTLV-QVLSSDVFARFLRKM---GEEVVFVSGSDEHGTPIEIEAIRKGIAPRDLTDKMHAY-ITWLF-ESFGISYDNY-TRT-ESDV----HKE----FVRDFYLKV--YNEGHIFERETEQLYCP-KDEMFLPDRFVT---------GTCP---YC-GYERAHGDQCDR-CGRLLNPTDLIDPKCS--ICGSVPE-MRR-TKHWFFDLPK-------------------FSER-LRKYI----EE-N---ENL--PENAKTLSL-SMI------------------------------------------------------------EEGLRPRSLTR----DNKW-GIPAPFP------------------------------------GSEGKTIYVWMEAVLGYVSAVK---EYFL-KRGE------AERFEEFWK-------------------------SGDTRSVYFI-GKDNIPF-HTIIFPALLMAS---------------GEGYALPFS----VASTEF-LLY-EGEK-FSKSER---RGIWMDE-ALQL--------------------L--PADYWRFYMIY-MRPELKD-ASFSWEDFESKVNDELNDTIGNLVHRIL   395
Adeh_1314_Adeh_86157740          5  LLVTSALPYANGPIHLGHLV-EYVQTDVYVRFRRAC---GDDVAYVCAADSHGTPIEVNAAKVGMTPKAFVEKYRAE-QHADF-RAFGVEFSTY-YTT-DSDE----NRR----WAYRVYDAL--KAKGLVYKKSVEQLYCE-TDRRFLPDRFVK---------GTCP---KC-GTPDQYGDVCEH-CGTTYDPRELKDPYCA--ICRSAPV-VRA-SDHAYVNLRK-----------------PDVNAV-IHEWV----NA-E---GHL--EPAVREQVK-GWL-------------------------------------------------------------ADLQDWCITR----DAPYFGFPVTDP------------------------------------EFPGKFLYVWVDAPIGYLSSAE---HFFAAEAPEGARLAPAEFERRYLA-----------------------VDSPARLEHFI-GKDILRF-HAVFWPAMLWAA---------------G--LKRPDR----MPVHGH-LTV-NGEK-MSKSRG---TFVTGRT-YLDSG-------------------L--DPELLRYFYAANLGSGVSD-LDLSLDEFRNRINADLANNVANLASRVA   407
Igni_1155_Ihos_156937943         4  WIVGSAWPYVNTVPHLGNLIGSVLSADVFARFLRLM---GEDVVFVSGSDEHGTPIEVEARKRGVEPKELTDKVHEY-VKKLF-EKYLISFDNY-TRT-HNPV----HME----FVRETFMKI--YENGYIFTQEMVMPYCP-KDKMFLPDRFTV---------GTCP---YC-GAPDARGDQCER-CGKLLDPPDLVNPRCA--FCGSRPV-WRK-TLHWFFDLPK-------------------AAEG-LVEWL----E--R---SEL--PNNVKKFTL-NWV------------------------------------------------------------KEGLTPRSVTR----DNKW-GIPAPFP------------------------------------GAEGKTIYVWFEAVLGYLSAVK---EL--DVK-N-----GTNLFEEFWK-------------------------DINSRPVYFI-GKDNIPF-HSIILPALLKAT---------------GEEYPLPYN----ISATEY-LMY-EGQK-FSKRRR---VGVWIDE-ALEV--------------------V-PNPDYWRFALIR-MRPEERD-TNFTWREFYRIVNSELNDDIGNFAHRVL   396
Ta1162_Taci_16082174             6  ILVNCALPYANGPLHIGHIAGAYLGADVFVRYNRLM---GNQVLYVSGSDEYGTPITVRAEKEGRSPKEIADIYYEE-HLRTF-ENLGISFDIF-MRT-TWPE----HSE----NAQDFFIKL--LNEGYIEKGTMIAPFCR-KIGRFMPDRYIE---------GTCP---YC-HYPKARGDQCDN-CGRTLDPQDLIDPKCI--LSGETPE-FRE-TEHFFLRLDL-------------------LEDR-LKSWI----SS-K----NFW-KPNVLAYTQ-NFI------------------------------------------------------------SGGLKKRPITR----DIDW-GVKIPLD------------------------------------GYDSKRIYVWFEALIGYITGAK---EY--SKRTG-----NPDLWKEYYM-------------------------DPEVRNYYFI-GKDNIPF-HAIIWPAMLMGY---------------G-GFNLPYD----IPANEY-LTF-KGQQ-FSKSRG---IGYSVDD-LLKA--------------------V--PADYLRYYVAS-ILPETGD-SDFSLEELVNTVNSDLIDKYGNLVYRIL   398
PTO0542_Ptor_48477614            5  ILVNCALPYANGPLHLGHIAGAYLAADIFVRFNRLN---GNEVLFVSGSDEYGTPITITAEKNKTSPQNVADIYHRE-HEQTF-KNLDIVFDIF-TRT-TDPE----HVK----DVDEFFINL--LNKNYLEKRYMVSPYCK-STGKFMPDRYIH---------GTCP---YC-GFNDARGDQCDE-CGRTLDPIELINPRCT--SSNEEPL-FIA-TEHFFLRLDL-------------------LSDE-LLNYL----NT-R---ENW--KPNVINFTR-SII------------------------------------------------------------NEGLRPRPITR----DIDW-GVPIPLN------------------------------------GFEGKRIYVWFEALIGYITGAR---VY--SKNIK-----DDDYWKKFWL-------------------------DKNVKSYYFI-GKDNIPF-HTIIWPAMLIAH---------------G-DYNLPYN----VPANEY-LRF-EGAQ-FSKSRG---IGYTVNE-ALSL--------------------V--NKNYLRYYMAS-IMPETGD-SSFSLNELVSRVNSELIDKYGNFIYRVL   397
MARS_Hsap_20178332             266  VLITSALPYVNNVPHLGNIIGCVLSADVFARYSRLR---QWNTLYLCGTDEYGTATETKALEEGLTPQEICDKYHII-HADIY-RWFNISFDIF-GRT-TTPQ----QTK----ITQDIFQQL--LKRGFVLQDTVEQLRCE-HCARFLADRFVE---------GVCP---FC-GYEEARGDQCDK-CGKLINAVELKKPQCK--VCRSCPV-VQS-SQHLFLDLPK-------------------LEKR-LEEWL----GRTL-PGSDW--TPNAQFITR-SWL------------------------------------------------------------RDGLKPRCITR----DLKW-GTPVPLE------------------------------------GFEDKVFYVWFDATIGYLSITA---NY-------------TDQWERWWK------------------------NPEQVDLYQFM-AKDNVPF-HSLVFPCSALGA---------------EDNYTLVSH----LIATEY-LNY-EDGK-FSKSRG---VGVFGDMAQDTG--------------------I--PADIWRFYLLY-IRPEGQD-SAFSWTDLLLKNNSELLNNLGNFINRAG   658
SPBC17A3.04c_Spom_19112378     224  ILITSALPYVNNVPHLGNIVGSTLSADVFARYHRAR---NHNTLYICGTDEYGTATETKALEEGVSPKELCDKYHAL-HKEVY-DWFEIDFDHF-GRT-TTPK----QTG----IAQHIFTKL--YNNDYMAIDTMTQLYCE-VHQGYLADRYVE---------GTCP---KC-GYDDARGDQCDG-CGGLLNAFELIDPKCK--LDRATPV-KRE-TKHVFLSLDK-------------------LQPA-VESWA----MQSA-VEGKW--SNNGRSITE-SWL------------------------------------------------------------KEGLRPRCITR----DLKW-GTPVPLE------------------------------------EFKGKVLYVWFDATIGYISITA---NY-------------TDEWEKWWR------------------------NPEQVKLYQFM-GKDNVPF-HTVIFPSSLLGT---------------GEKWTMLHH----INTTDY-LNY-ETGK-FSKSRG---VGVFGNTAQDIG--------------------L--SPSVWRYYLLS-SRPETSD-TMFTWKEFITRHNSELLANLGNFVNRTL   616
metS_Ddis_66815711              18  ILITSALPYVNNVPHLGNIIGCVLSADVYARYCRLK---NYNCIYICGTDEYGTATETKALSEGCTPKEICDKYHEI-HKEIY-EWFNISFDKF-GRT-STNS----QTE----IAQDIFNKI--KDNGYTLTQEIEQLYCEQTCKMFLADRFVE---------GTCP---HC-KFEDARGDQCDG-CSKLLNPTELINPRCK--VCSKPPV-IKS-TKHIFIDLPQ-------------------LQQQ-VDQFV----ETNS-KGGNW--SENSIAITN-TWV------------------------------------------------------------KGELKPRCITR----DLKW-GTPVPME------------------------------------EFKDKVFYVWFDAPIGYISITA---EY-------------TNEWEKWWK------------------------NPENVKLVQFM-GKDNVPF-HTVIFPASLIGS---------------KDNYTLLNN----LSTTEF-LNY-ETGK-FSKSRN---TGVFGDGAKATG--------------------I--PSEVWRFYLLN-NRPESSD-SIFSWDDFNFK-NNELLNNFGNLVNRVL   410
AT4G13780_Atha_15236350         19  ILITSALPYVNNVPHLGNIIGCVLSADVYARYCRLR---GYNAIYICGTDEYGTATETKALEENCTPKEICDKYHAI-HKEVY-DWFGISFDKF-GRT-STPE----QTE----VCQAIFNKL--WDNKWLSENTMQQLYCD-TCKKFLADRLVE---------GSCPFE-GC-NYDSARGDQCEK-CGKLLNPTELKDPKCK--VCQNTPR-IRD-TDHLFIELPL-------------------LKDR-LEAYI----KKTS-VTGSW--SQNAIQTTN-AWL------------------------------------------------------------RDGLRQRCITR----DLKW-GVPVPHE------------------------------------KYKDKVFYVWFDAPIGYVSITS---CY-------------TSEWEKWWK------------------------NPENVELYQFM-GKDNVPF-HTVMFPSTQLGT---------------EENWTLMKT----ISVTEY-LNY-EDGK-FSKSKG---VGVFGNDVKDTN--------------------I--PVEVWRYYLLT-NRPEVSD-TSFSWTDLQAKLNGELLSNLGNFVNRVL   413
metG_Tpal_15639785               4  KLITAALPYVNNVPHLGNLI-QGLSADVFARFCRMR---GYHTCFVCGTDEYGTASETRAAEQGLSPAQLCAHYHAL-HRDIY-QWFDLSFDYF-GRT-TSDA----HTE----LTQALFRHL--DARGFISEHESAQAYCL-HCARFLADRYLR---------GTCP---HC-RNAEARADQCEH-CGVLLEPETLLNARCV--SCGTAPE-FRP-TRHLYLNLPA-------------------LEKA-YRSWF----CTT----NHLW-TKNAVRMTE-GWL------------------------------------------------------------RTGLQERAITR----DLRW-GVPVP------------------------------------KAGFEQKVFYVWFDAPVGYISITKCGTEAASSQEGGGTDDGVKEKWQSWWL------------------------DQQDVELVQFV-GKDNIPF-HTLFFPCMLIGS---------------GQRWTMLTR----LSATEY-LNY-EGGK-FSKSLG---VGVFGSD-AKESG-------------------I--PSDLWRFYLLY-HRPEKSD-AHFTWHEFQERVNSELIGNLCNLVNRTL   409
metG_Bbur_15594932               5  NLVTAALPYVNNIPHLGNLV-QVLSADAFARYSKMS---GIETLYVCGTDEYGTATETKALIENTTPLELCNKYYEI-HKSIY-KWFNIEFDIF-GRT-TNKN----HQD----IVQNFFLQL--EKNGYIKERETEQFYCN-KDSMFLADRYVI---------GECP---EC-QSM-AKGDQCDN-CSKLLNPTDLINPKCI--ICKNKPI-LKK-TNHLYLDLPK-------------------IKTK-LEKWI----KNPD-TSKNW--NTNALKMTK-AFL------------------------------------------------------------RDGLKERAITR----DLKW-GIPVP------------------------------------KKGFENKVFYVWFDAPIGYISITK----------------NIIKNWESWWK------------------------NNDQVNLVQFI-GKDNILF-HTIIFPCIEIGS---------------EENWTILNQ----LSSSEY-LNY-ENLK-FSKSEG---TGIFGND-AITTG-------------------I--PSDIWRFYIYY-NRPEKSD-FQFMWQDLMERVNTELIDNFSNLVNRVL   395
MXAN_2804_Mxan_108762836         5  TLVTSALPYANGPLHIGHAV-EYVQTDIYVRFLRSC---GRDVVYFCADDTHGTPIELNAAKQGLKPEEFIARFHEE-HQRDF-HDLDVRFDYF-HST-NSPE----NRQ----YAELIYGRL--KEKGDIERRNIEQTYCE-NDRRFLPDRFIK---------GTCP---NC-KASDQYGDACEK-CGKAYDPTDLIDARCA--LCGTPPV-RKH-SEHLFFKLSR-------------------HEDF-LQDVL----R--K---PGFI-HPGLATQLQ-GFF------------------------------------------------------------EKGLSDWDISR----DGPYFGFAIP--------------------------------------GETDKYFYVWLDAPIGYIATTE---KWA--KETG-----KAKSALDYWS------------------------ADADTRIIHFI-GKDIVYF-HALFWPAVLNVA---------------G--FHIPSE----IKVHGH-LML-NGEK-MSKTRG---TMVPVRD-YLDQ--------------------L--DPSYLRYFYAANLGPGVED-LDLNLKDFRQRVNGELVNNVGNLANRAL   396
metG_Lint_45658951              10  ILVTSALPYANGPIHLGHVL-EGIQTDIWVRFQKAI---GNECYFFCADDTHGTPVMLAARKEGITPEQLIERVGQE-HYRDL-TSFGIEYDHY-DST-HSKA----NQE----ISKDIYLKL--KSKGHISRRSIEQSYCE-TDKMFLPDRFIK---------GTCP---NC-KSKDQYGDNCEV-CGATYSPKDLIDSHCS--LCGTSPV-VKN-SDHIFFKLGDFHKKDEKSTSLETINPSHLKTDFDLQSWI----E-TS---GVVSESEGVKKKLK-EWF------------------------------------------------------------DAGLQDWDISR----DGPYFGFEIP--------------------------------------DETNKYFYVWLDAPIGYMASSK---NFF--EKNFP---NEPNKFDSFWK-------------------------NKNSEIVHFI-GKDILYF-HTLFWPAMLEGS-----------------DYRAPSK----VHVHGF-IGV-NGEK-MSKSRG---TFIKAET-FVKY--------------------L--DPEHLRFYLASKLGPGMDD-IDLSFEDFINKVNSDLVGNLINSVSRVS   424
SCO6436_Scoe_21224742            8  FLVTATPPTTNGDVHVGHLSGPYLGADVFSRAQRML---GHTVLYASGGDDHQTYVVTTAERLGLDPVELAARCNRE-IVGTL-ELAGIDIDAF--TS-PDDA----YRA----EVREFFTGL--HRAGRLKTRTWTFPYCG-RTGRYLLEAFAT---------GYCP---EC-LVGTC-GAICEN-CGHPNDVDSLLFPASTGAGPAATTQ-PRE-TEILVLPLEE-------------------YREQ-FTEFY----R--A---RRATMRPHVLRFVD-EML------------------------------------------------------------SRPLPDFPVSY----PADW-GIPVGI------------------------------------DGFDGQVFNVWAEMLPGLRHMAE----AARARR-------GPATPPGVWA------------------------ADSGFELVQFL-GYDNTFY-FAFAHLGLTFAHG--------------G--LTEPAA----IVTNEF-YHL-DGAK-FSTSRR---HLVWARD-LVGK--------------------Y--GADNVRFHLAL-DNPEHQP-ANFTEADFLDTVRTRLHQPLQSIAAALA   401
metG_Acit_120612475              6  IFVTTALPYANGNFHIGHIM-EYIQADIWVRFQRMQ---GAEVNFVGADDTHGAPIMIAAEKAGKTPQQFVADIAAG-RKPYL-EGFHIRFDNW-HST-DAPE----NHE----LARQIYRDL--QAAGLIETRTIEQFFDP-EKNMFLPDRFIK---------GECP---RC-HARDQYGDNCEN-CGAVYAPTDLIEPYSA--LSGAKPV-LKS-SDHFFFQLSD-----------------PRCVAF-LQEWT----Q--D---GRL--QPEVANKVK-EWFSVRTNPDGTT--------------------------------------------------SEGLGDWDISR----DAPYFGIEIP--------------------------------------DAPGKYFYVWLDAPVGYLASLK---NLL--EK-------RGQSYDDYVA-------------------------DPQLEQVHFI-GKDIVTF-HTLFWPAMLKFS---------------G--RKTPDA----VFVHGF-LTVNNGEK-MSKSRG---TGLDPLK-YLGLG-------------------M--NAEWLRYYLAAKLNGRNED-IDFNAEDFMARVNSDLIGKFVNIASRAA   407
metG_Bxen_91782348              19  ILVTSALPYANGQIHIGHLV-EYIQTDIWVRTLRMH---GHEVYYVGADDTHGTPVMLRAEKEGLTPKQLIDRVWQE-HKRDF-DSFGISFDNY-YST-DSEE----NRV----LSENVYLAL--KEAGLIDARDIEQAYDP-VKEMFLPDRFIK---------GECP---KC-GAKDQYGDSCEV-CGSTYLPTELINPYSV--VSGATPV-RKT-STHYFFRLSD-----------------PRCENF-LRAWV-----------GGLA-QPEATNKMR-EWLGDAG--------------------------------------------------------EAKLADWDISR----DAPYFGFEIP--------------------------------------GAPGKYFYVWLDAPVGYYASFK---NLA--EK-------RGLDFDAWVRK------------------------GSKAEQYHFI-GKDILYF-HTLFWPAMLEFS---------------G--HRTPTN----VFAHGF-LTV-DGAK-MSKSRG---TFITAQS-VIETG-------------------L--NPEWLRYYFAAKLNSTMED-LDLNLDDFQARVNSDLVGKYVNIASRAA   413
metG_Rsol_17547100               6  ILVTSALPYANGPIHIGHMV-EYIQTDIWVRFQRMR---GHETYYVGADDTHGTPVMLRAEKEGITPRQLIERVWTE-HKRDF-DNFLVSFDNY-YST-DSDE----NRE----LCERVYLKL--KEAGLIDVREVEQFYDP-VKEMFLPDRFIK---------GECP---KC-GAKDQYGDSCEV-CGATYQPTDLKNPYSV--VSGATPV-RKS-SEHYFFKLSD-----------------PRCETF-LRDWV-----------GDLA-QPEATNKMR-EWLGDEG--------------------------------------------------------ESTLSDWDISR----DAPYFGFEIP--------------------------------------GAPGKYFYVWLDAPVGYYASFK---NLC--GK-------LGLDFDAWVS------------------------THSTAEQYHFI-GKDILYF-HTLFWPAMLQFS---------------G--HRTPTN----VFAHGF-LTV-DGAK-MSKSRG---TFITAQS-YIDTG-------------------L--NPEWLRYYFAAKLNATMED-LDLNLDDFIARVNSDLVGKFVNIASRSA   400
metG_Pdis_150009383              8  TLITTALPYANGPVHIGHLAGVYVPADIYARYLRLK---GEEVLMIGGSDEHGVPITLRAKKEGITPQDVVDRYHGI-IKKSF-EEFGITFDIY-SRT-TSAT----HHQ----MASDFFRTL--YDKGEFIEKTSEQYYDE-EAKQFLADRYIM---------GTCP---HC-GNEKAYGDQCEA-CGTSLSPTDLIDPKSA--ISGSKPV-MRE-TKHWYLPLDK-------------------WEPF-LRKWI----LEDH---KEW--KPNVYGQCK-SWL------------------------------------------------------------DMGLQPRAVSR----DLDW-GIPVPV------------------------------------EGAEGKVLYVWFDAPIGYISNTK---EL------------LPDSWETWWKD-------------------------PETKMVHFI-GKDNIVF-HCIVFPSMLKAE---------------G-SYNLPEN----VPANEF-LNL-EGDK-ISTSRN---WAVWLNE-YLVD--------------------MPGKQDVLRYVLTA-NAPETKD-NDFTWKDFQARNNNELVAILGNFVNRAL   398
metG_Hwal_110667531             29  AVVTCGLPYANGDLHIGHLR-TYVGGDIFSRALKRL---GQETAFVSGSDMHGTPVAVNAAEEGVTPESFALRHHEQ-YETTF-PQFGIEFDNY-GHT-HDET----NVE----MTREIVEAL--IQAGYVYEREIPVAYDP-AADQWLPDRFVN---------GTCP---YC-GAHAR-GDECDEGCGRHLEPGEIETPVST--ITGNDAE-YRR-REHQFFAVSE-------------------LQSY-LSSFL-----------DRLEGTTNAQNQPR-EWV------------------------------------------------------------EGELQDWCITR----DMDW-GVDYP--------------------------------------DDNDLVLYVWVDAPIEYISSTK---QYA--ERVGT----DSFDWKNAWRDIPGVRSHSSSSAKDSSEGNSPSNINNGGEIIHII-GRDIIQH-HTVFWPAMLHAA---------------G--YTEPRA----VMASGF-ITL-EG-KGFSTSRN---RAVWADE-YLAEG-------------------F--HPDLLRYYLAT-NGGFQQD-VDFSWSRFRERVNNELVGTVGNFIYRSL   443
metG_Pent_104780428              7  ILVTSALPYANGSIHLGHML-EYIQTDMWVRFQKLR---GNQCVYVCADDAHGSAIMLRAEKEGITPEQLIANVQAE-HSSDF-ADFLVDFDNF-HST-HSEE----NRE----LSGLIYTRL--RDAGHIATRSVTQYFDP-EKGMFLADRFIK---------GTCP---KC-AAEDQYGDNCEK-CGATYAPTELKNPKSA--ISGATPV-LRD-SQHFFFKLPD-------------------FQAM-LQQWT----R--S---GTL--QDAVANKLA-EWL------------------------------------------------------------DSGLQEWDISR----DAPYFGFEIP--------------------------------------GEPGKYFYVWLDAPIGYMASFK---NLCA-RR-------PELDFDAFWSE------------------------GSKAELYHFI-GKDIVNF-HALFWPAMLEGS---------------G--FRKPTA----VNVHGY-LTV-NGAK-MSKSRG---TFIKART-YLDH--------------------L--QPEYLRYYYAAKLGRGVDD-LDLNLEDFVQKVNSDLVGKVVNIASRCA   396
PSEEN0982_Pent_104780203       113  TLIFATPPTPNGDLHLGHLSGPYIAGDVLKRVLAGA---GAQVFYGSGRDDNQTYVVTCAAREGFSPSECADHYAEA-IRETW-QGYGIDMDFFITPD-NQGE----YAD----FVHHYLQRL--YDQGLIYAKDTPVFVDN-Q-GNALHEAFIH---------GGCP---HC-GESSD-GNACEA-CGQPNQCVDLTQPRVK--QDGRQPQ-LKI-ETRLFFRLSA-------------------LADE-LASYV-----------QTANMPAHVYQLCH-TML------------------------------------------------------------SQGLPDICISH----RSDW-GIRHRL------------------------------------PGMQDQVVYVWFEMAFGYLWAAS---ELPG-AE-------GDRVARAAQRY------------------------AGAMDIVHCY-GFDNAYY-HTLLFPAVYLAL---------------G--LTPPRH----HVVNEL-LDL-NGSK-FSTSRR---HLIWGKD-FLTE--------------------A--GADYARMALML-TRPEGVR-TNFTVDQVCERINDLFADKLTHWVQRLQ   502
Dd703_3089_Ddad_242240498      122  QFVFSTPPTPNGDLHLGHLSGPYLGADVYVRYQRMI---GNPAWHMTGSDDYQSYVVALAERENTTPAAVAQHFSRE-ILQTL-KMMDIEPDHYTVTN-DAAG----YIQ----GAQRFFSRT--LGEDKVVFAESAALRDA-QNGQYLYEVDVS---------GQCP---GC-GGGTN-GNICEE-CGEPNVVTDMAQPLSK--RSQLPPE-RTQ-VARYLLPLHR-------------------FEQQ-IAEHH----K--V---GRV--PARMRELAH-RVF------------------------------------------------------------SRPEFTVPVSH----PSEW-GIAPA------------------------------------ETEGEGQVIWVWPEMSYGFLYDIE---Q-------------LGKKINRPWSA---------------------DEPGKDWKIVHFF-GYDNSFY-HSILYPALYHLAY--------------P-EW-QPDI--D-YHYNEF-YLL-DGKK-FSTSRR---HAVWGKE-ILTPE----------------------SVDAVRFYLCL-TRPEQER-TNFRLSQWEDEVRDTLLGEWQTWLHDLG   512
Sare_1044_Sare_159036691       123  VFVFSTPPTPNGDLHLGHLSGPYLGADAFVRFQRMN---GTEAWHLTGSDDYQSYVVNTARREGRAPAETAARYSAE-IAQTL-AMMDINPDQYTVTD-TEPG----YRQ----GLRNFFSQV--IASGRATVTERDALFDG-ESGRYLYEADVR---------GGCP---GC-GESTS-GNICEE-CGEPNTVVDLRQPRSN--ESDAEPR-RAP-LARWSLPLHQ-------------------FRDE-VSTHH----S--L---GRV--PARLRELGD-RLF------------------------------------------------------------ARPVLDIPLSH----PADW-GVPPAEK------------------------------------DVDDQVIWVWPEMSYGFLHGIE---A-------------LGARLGRGWQAAV---------------------PEQDWKIVHFF-GYDNSFY-HAVLYPVLYRLAHP-------------G--W-QPDI--D-YHVNEF-YLL-EGEK-FSTSRR---HAIWGKE-ILDED----------------------TVDAVRYFLSR-TRPEAER-TNFRRADFRSVLHDTLIGTWQRWLNDLG   513
Francci3_3273_Fsp._86741959    122  VFVFSTPPTPNGDLHLGHLSGPYLGADAYVRFQRMN---GANIWHLTGSDDFQSYVVDCARREGRSNAETAAHYSAE-IAETL-RLMDIPLDQYTVTN-ADPS----YVP----ALKDFFTRL--VDSGAIAPSDGPALFDP-KTGQYLYEVDVR---------GGCP---GC-GEITG-GNICEE-CGEPNTVVDLADPVAK--PSGEAPA-AGT-VRRYVLPLHE-------------------HAGT-VLDHH----R--R---GRV--PARLRELAA-RLF------------------------------------------------------------RRERVDIPVSH----PSDW-GITPH------------------------------------EANGAGQVIWVWPEMAFGFLHGIQ---K-------------LGERIGRDWTAA----------------------EPTDDWKIVHFF-GYDNSFY-HAILYPVLYQLALP-------------G--W-TPDI--D-YHVNEF-FLY-DGLK-FSTGRR---HAIWGKQ-ILDAG----------------------SVDAVRYFLAR-SRPEAQR-TNFELDAYEHTLATTLIGTWQAWLNDLG   512
metG_Ddad_242238640              8  ILVTCALPYANGPIHLGHML-EHIQADIWVRYQRMR---GNQVHFICADDAHGTPIMLKAQQMGLAPEQMIEQVSQE-HQRDF-TGFDISFDNY-HST-HSEE----NRE----LSGLIYRRL--KENGFIKSRTISQLFDP-EKSMFLPDRFVK---------GTCP---KC-KAPDQYGDNCEV-CGATYSPTELIDPKSA--VSGATPV-MRD-SEHFFFDLPA-------------------FSDM-LQSWT----R--S---GAL--QEQVANKMQ-EWF------------------------------------------------------------DAGLQQWDISR----DAPYFGFEVP--------------------------------------DAPGKYFYVWLDAPIGYMGSFK---NLC--DKR------GDIHFDEFW------------------------SKDSTADLYHFI-GKDIVYF-HSLFWPAMLEGS---------------G--FRKPTN----LFVHGY-VTV-NGAK-MSKSRG---TFIKAET-YLKH--------------------L--DADCLRYYYAAKLSSRIDD-IDLNLEDFIQRVNADIVNKVVNLASRNA   397
metG_Hinf_16273191               8  ILVTCALPYANGAIHLGHML-EHIQADIWVRFQRMR---GNKIHFVCADDAHGTPIMLNADKLGITPEELIAKAKAD-HIRDF-AGFNISFDNY-HST-HSEE----NKQ----LTAEIYNKL--KANGFIKSKVISQLFDP-EKNMFLPDRFVK---------GTCP---KC-KAEDQYGDNCEV-CASTYSPMDLINPRSA--VSGTTPI-VKE-SEHFFFDLPA-------------------FEGM-LKEWT----R--S---GSL--QSEIANKMQ-EWF------------------------------------------------------------ESDLQQWDISR----DAPYFGFEIP--------------------------------------GAKDKFFYVWLDAPIGYMASFK---NLC--NR-------EGIDFNEFWA------------------------EGSDAELYHFI-GKDIVYF-HSLFWPAMLEGS---------------G--YRKPTN----VFAHGY-VTV-DGAK-MSKSRG---TFIQAST-YLNH--------------------I--DPECLRYYYAAKLNDRIED-LDFNLEDFVQRVNTDIVNKLVNLASRNA   396
metG_Srub_83814986              10  LLVTAALPYANGPIHIGHLAGAYLPADLFVRYQRLK---GEDVAFICGSDEMGVAILMRAIREDRTPEDIIDTYHPQ-IRDNF-ERFGMSFDYY-GRT-SSET----HTE----TTQDFFRVL--DENGGFDLKTDEQLYDP-EAEMFLADRFVI---------GTCP---VC-GFEEAYGDQCEQ-CGSSLSPTELENPQST--LTDATPE-FKE-TTHWYLPLGE-------------------LQPQ-LEEWI----G-SH---PEW--KNNVVGQIQ-SWF------------------------------------------------------------DEGLKGRAITR----DVLW-GVPVP-----------------------------DDVAERHGLEAEGKVIYVWFDAPIGYISATK---EWAAEQG-------EPDAWTDYW-------------------------QDEDTRLVHFI-GKDNIVF-HCLMFPSMLMEH---------------G-DYVLPDN----VPANEF-LNL-EGEK-LSTSRG---WAVWLHE-YLDDFADER---------------H--APDLLRYALAT-TLPETKD-ADFSWEGFQQRVNGELANVFGNFVHRTL   414
Caci_6318_Caci_256395449       123  QFVFSTPPTPNGDLHLGHLSGPYLGADVYVRYQRLL---GNRAWHLTGSDDYQSYVTAVAAKDGVTPAEAAAHYSAE-IAATL-KAMDIDVDEYYVTS-VAEG----YPE----GLQGYFSRL--VDSGHVSPASTPALFDA-ETHEYLYEVNVG---------GSCP---AC-FSPAG-GNICEE-CGEPNLVADLVDPVSG--ISGTAPK-QAD-AERYTLPLHE-------------------FADI-VADHH----R--R---GRV--PIRLRELAQ-RVF------------------------------------------------------------AHERLDLPVSH----PSGW-GVQPAED------------------------------------DLDGQVIWVWPEMSYGFLHGIE---R-------------LGAELGELW---------------------SSREPSRDWKIVHFF-GYDNSFY-HSILYPVLYRLAH--------------P-EW-NPDI--D-YHVNEF-YLL-EGQK-FSTSRR---HAIWGKE-ILNPG----------------------TVDAVRYYLSS-TRPEGER-TDFRRAEFDRACAETLVGGWEQWLHDLG   513
metG_Ecol_16130052               8  ILVTCALPYANGSIHLGHML-EHIQADVWVRYQRMR---GHEVNFICADDAHGTPIMLKAQQLGITPEQMIGEMSQE-HQTDF-AGFNISYDNY-HST-HSEE----NRQ----LSELIYSRL--KENGFIKNRTISQLYDP-EKGMFLPDRFVK---------GTCP---KC-KSPDQYGDNCEV-CGATYSPTELIEPKSV--VSGATPV-MRD-SEHFFFDLPS-------------------FSEM-LQAWT----R--S---GAL--QEQVANKMQ-EWF------------------------------------------------------------ESGLQQWDISR----DAPYFGFEIP--------------------------------------NAPGKYFYVWLDAPIGYMGSFK---NLC--DKRG-----DSVSFDEYW------------------------KKDSTAELYHFI-GKDIVYF-HSLFWPAMLEGS-----------------NFRKPSN----LFVHGY-VTV-NGAK-MSKSRG---TFIKAST-WLNH--------------------F--DADSLRYYYTAKLSSRIDD-IDLNLEDFVQRVNADIVNKVVNLASRNA   398
metG_Scel_162456166              4  LLLTSALPYANGHIHIGHLV-EYTQTDIFARYWRMT---GRRCISLCADDTHGTAIMIRARQEGRSEADVIADMSAA-HQRDF-AAFQIRFDHY-GST-NSPA----NRA----LCEEIWASL--RERGMVTTKEVTQLFDP-QEGMFLADRFVK---------GTCP---KC-AAPDQYGDSCDR-CGSTYAATDLVEPRSA--LTGARPE-VRS-AQHLMVAIEP-------------------ERPF-LSTWT----QG-E---GRM--PKEIANYLAGHFL------------------------------------------------------------SEPLRDWDVSR----PAPYFGFEIP--------------------------------------DAPGNYWYVWFDAPIGYMAASK---EWC--DR-------EGEAFDDWW-------------------------RSEETEIVHVI-GKDIVYF-HTLFWPAMLKSA-----------------RFSLPSR----VQVHGF-LTV-NGEK-MSKSKG---TFVLAST-YLKH--------------------L--DPAYLRYYYASKLSSKVDD-IDLNLEELVNKVNAELVNKVVNLASRSS   393
metS_Scel_162456513             24  MVVTAGMPYANGPLHLGHLAGAQLPADIYARWCRMLI-GAENVLYVCGTDEHGSTSEIAAVQAGLPIREYIDRIQQA-QKKTL-ERYCISLDAF-TGT-SQPETFPLQRE----LAHEFLTRF--SRNGLLEKRATRQWFDP-KMGRFLPDRYVR---------GRCPNP-QC-DNEDAYSDECDR-CGQQHAPAALLNPRST--LSDAVPE-LRE-TVHWWLDMWS-------------------VSET-LREWI----SGKE---KTW--RRAMLSEVL-ETVMPSVRLDRAHEATYKEIKASLPKHKSKLARGKELVVQFGNKADLESGRAILEQHGVRVELVDEWAHRSITR----DIGW-GIPITED-----------------------------------PDLAGKTLYVWPDSLIAPISFSK---VALR-DKGV-----DPARYADFW-------------------------RDPEARVVQFL-GQDNVYF-YVLMQGAMWLGTQ-EDPRR------L-PAAGELQLT--D-VFGCFH-LLV-GGEK-MSKSRG---NFFTGDQ-LLDEKG------------------Y--SVDQIRYYLAL-LSLAEKP-SDFDFEKLDER-NKFLAGPMNAAFERPI   497
metG_Ctep_21673797               8  TLVTTALPYANGPVHLGHLAGVYLPADIYVRYKRLC---GHDVIHIGGSDEHGVPITITADKEGISPQEVVDRYHTM-NAEAF-AKCGISFDYY-GRT-SGPV----HHQ----TAREFFLEI--EKKGIFVKKTEKQFFDP-KAGRFLSDRYIT---------GTCP---VC-KTPGANGDQCEQ-CGTHLSPTELIDPKSK--LSDATPE-LRE-TLHWYFPLGR-------------------YQKQ-LEAFV----ERHT---GDW--RSNVVNYSR-TWL------------------------------------------------------------NQGLADRAITR----DLAW-GISLPLDS----------------------------------EEAKGKVLYVWFDAVLGYISFTK---EWA--EKQG-----DAELWRRYW-------------------------QDPETRIINFI-GKDNVVF-HTLMFPAILMAWN-EGRSE--------G-RYELADN----VPASEF-MNF-EGRK-FSKSRN---YAVYLGE-FLER--------------------F--PADTLRYSIAM-NYPENKD-TDFSWSDFQNRTNGELADTLGNFIKRSI   409
metG_Paer_15598678               7  ILVTSALPYANGSIHLGHML-EYIQTDMWVRFQKMR---GNQAVYVCADDAHGSAIMLRAEREGITSEQLIDAVRAE-HMGDF-ADFLVDFDNY-HST-HSEE----NRE----LSSAIYLKL--RDAGHIDTRPVTQYFDP-EKQMFLADRFIK---------GTCP---KC-GTADQYGDNCEA-CGATYAPTELKDPKSA--ISGATPV-LKE-SLHYFFKLPD-------------------FEAM-LKQWT----R------SGAL-QESVANKLA-EWL------------------------------------------------------------DSGLQQWDISR----DAPYFGFEIP--------------------------------------DAPGKYFYVWLDAPIGYMASFK---NLCA-RR-------PELDFDAFW------------------------GKDSGAELYHFI-GKDIVNF-HALFWPAMLEGA---------------G--YRKPTA----LNVHGY-LTV-NGQK-MSKSRG---TFVKART-YLDH--------------------L--DPEYLRYYYASKLGRGVED-LDLNLEDFVQKVNSDLVGKVVNIASRCA   396
NEQ457_Nequ_41615241             3  ILVTAALPYSNGPIHLGHIAGAYLPADIFYRFVKLK---GYNALYICGSDQYGSPIELNAIKLNIDPKDYASFYRKI-QEEIF-KKFNIKFDIY-SGTAESNI----HPI----IVKEFFLSL--FSAGLLIEKEQELPYDP-KIKRFLPDRFVV---------GQCP---YC-GYEKAYGDQCEK-CGRLLEPKELINPKSA--ITGEKVI-FKK-TRHLFFNIPK-------------------LKDK-LKQYI----ES-K---KDVW-NDFTYSWSL-ALL------------------------------------------------------------DN-FKERAITR----DNKW-GVKVPAKEMLEILKKALKEGKTPKDFGLLIDSTNEKDLENHIKEYENKVLYVWFDAPIGYISFTF---E-------------TSPEYRYYW------------------------DEKEKPYIVHFI-GKDNIPF-HTIFWPALIIGRNLGYKNINHILD-F---DIALPYQ----VFGNPY-LNY-YGKK-FSKSKR---WGVFLDN-IDKID-------------------I--DIDYFRFYLAY-IHTVSKD-MSFEWDQFKEVINKELVDNIGNFIHRVL   440
metG_Cpin_256426015              7  YLITAALPYANGPVHIGHLAGCYIPADIYVRYLRAK---KADVKFIGGTDEHGVPITIKAMKENVTPQDIVDKYHKI-IYDSF-TDMGISFDIF-SRT-TKQV----HHE----TAADFFKTM--YDKGLFEEKESEQFFDE-TANVFLADRYIV---------GTCP---KC-GNPNAYGDQCER-CGTSLSPDELIEPRST--LSNAVPV-KKK-TKHWYMPLQN-------------------YEPF-LKEWL----LEGH---KEW--KNNVYGQCK-SWI------------------------------------------------------------DGGLQSRAMTR----DSSW-GIQVPLP------------------------------------DAEGKVLYVWFDAPIGYISATK---E-------------LTANWADYW-------------------------CKDDTKLVHFI-GKDNIVF-HCVIFPAMLKGH---------------G-GYVLPEN----VPANEF-LNI-ESEK-VSTSRN---WAVWVHD-YVKD--------------------FPDQQDVLRYVLTS-IAPETKD-SDFTWKDFQQRNNSELTDIFSNFVHRTM   396
metG_Bthe_29348342               8  TTVTSALPYANGPVHIGHLAGVYVPADIYVRYLRLK---KEDVLFIGGSDEHGVPITIRAKKEGVTPQDVVDRYHYL-IKKSF-EEFGVSFDVY-SRT-TSKT----HHE----LASDFFKTL--YNKGEFIEKTSEQYYDE-EAKTFLADRYIT---------GECP---HC-HSEGAYGDQCEK-CGTSLSPTDLINPKSA--ISGSKPV-MKE-TKHWYLPLDK-------------------HEGW-LRQWI----LEDH---KEW--RPNVYGQCK-SWL------------------------------------------------------------DMGLQPRAVSR----DLDW-GIPVPV------------------------------------EGAEGKVLYVWFDAPIGYISNTK---EL------------LPDSWETWW-------------------------KDPETRLLHFI-GKDNIVF-HCIVFPAMLKAE---------------G-SYILPDN----VPSNEF-LNL-EGDK-ISTSRN---WAVWLHE-YLED--------------------FPGKQDVLRYVLTA-NAPETKD-NDFTWKDFQARNNNELVAVYGNFVNRAM   398
mlr5926_Mlot_13474936           37  IFVFSTPPTPNGDLHLGHLSGPYLGADVYTRFLRMK---GVEAYHLTGSDDYQSYVATRADAEQSTPGKVARHYADE-IRATL-ALLDCEVHSFLPTL-GDSA----YAE----FQAACFRSL--LSSTAVDLRQSPALFDA-VTGDYLYEPDVS---------GLCP---DC-GGSAG-GNICEE-CGAPNLCHDLDAVRSR--HSAEAPV-VGF-MRRPELALER-------------------CYVN-IDRHL----RA-----SGA--PVRIMDLFA-RLR------------------------------------------------------------QRGDFSVPIIH----PSDW-GLPAE--------------------------------------GFPGQVTWVWPEMAFGFLYNIQ---T-------------LATSLGRHWNAAL---------------------PSKDWQIVHFF-GFDNSFY-HALLYPALYAEV---------------FSHWTPRIR----YHVNEF-YLL-DGQK-FSTSRS---HAVWGKE-VLGQ--------------------K--TVDVVRLHLGL-TRPEGER-TNFTLDALRRTENEVFQGIWLNWLDALH   425
metG_Cpha_145219579              7  TLVTTALPYANGPVHLGHLAGVYLPADLFVRYKRLQ---GEDVIHIGGSDEHGVPITITAEKEGITPRDVVDRYHSM-NLEAF-KRCGISFDYY-GRT-SSEL----HHK----TAQEFFLEI--EGKGIFERKTEKLFYDA-SACRFLSDRYVT---------GTCP---IC-GNTEANGDQCEQ-CGTHLSPLELINPKSK--LSDATPE-LRE-TLHWYFPLGR-------------------FQKQ-LEAFV----GSHD---DDW--RANVLNYTR-TWL------------------------------------------------------------NQGLNDRAITR----DLSW-GIKVPL----------------------------------QDPEAEGKVLYVWFDAVLGYVSFAR---EWAVLQG-------SPDRWKEYW-------------------------QNPDSRVVNFI-GKDNVVF-HTLMLPAILMAWN-EGRSD--------S-IYNLADN----VPASEF-MNF-EGRK-FSKSRN---YAVYLGE-FLDK--------------------F--PAETLRYSIAM-NYPENKD-SDFSWTDFQNRTNGELADTLGNFIKRSV   408
metG_Cvio_34496661               9  ILVTSALPYANAGLHLGHML-EQIQTDIWVRFQKMR---GHECYYVCADDTHGAPIMLAAEKRGITPEQLVNEVREL-HVADS-QGFLIGHDNY-YST-NSPE----NKA----LAEQVYLAL--KADDKIACRTIEQLFDP-EKQMFLPDRFVK---------GECP---KC-SAKDQYGDNCEV-CGATYAPTELKNPYSA--VSGAKPV-LKT-SEHFFFRLGE-------------------CADF-LKAWTSGASRRADGAVQPHL-QPESLNKMN-EWI------------------------------------------------------------GGGLQDWDISR----DAPYFGFEIP--------------------------------------GAPGKYFYVWLDAPIGYMASFK---NLC--ER-------LNLNFDEWFA------------------------KDSQTEMYHFI-GKDILYF-HALFWPAMLNYS---------------G--LRAPTG----VFAHGF-LTV-DGQK-MSKSRG---TFIQAKS-YLDCG-------------------L--NPEWMRYYIAAKLNGRIED-IDLNLNDFVARVNSDLVGKFVNIASRSA   408
PPA1728_Pacn_161621793           5  VLAAVAWPYANGPRHIGHVSGFGVPSDVFARYMRMS---GHRVLMVSGSDCHGTAISVKADQEGVTAQECAEKYHRI-IAADL-QGLGLSYDLY-TST-LTDN----HAH----VTQEIFTRL--HENGYVVKRSEMGAFEP-STGRTLPDRYIE---------GTCP---VC-GYDDARGDQCDN-CGRQLDPADLIGPRSK--TTGAAPE-FRE-TEHFFLDLPA-------------------LAES-LASWI----DT-R---TDW--RPNVLKFSH-NLL------------------------------------------------------------EE-LRPRAITR----DLDW-GIRVPVEGW---------------------------------QDNPMKSIYVWFDAVIGYLSASI---EWA--RRIG-----RPDAWREFWN-------------------------DEDARSYYFM-GKDNIVF-HSVIWPGILLGTNGRGDKGGEPSEEL-G-TLNLPTE----IVSSEF-LTM-SGSK-VSNSRG---ATIFVGD-FLHE--------------------F--GPDALRYFIAV-AGPENQD-TDFTWEEFVRRVNFELANEWGNLVNRSI   413
metG_Rbal_32473761               5  LLVTAALPYANGPIHIGHLV-EYLQTDIWVRFQKLR---GNRCLYICADDTHGTAIMIRARGEGRSEIELIEETSEA-HQRDF-AGFGIEFDHY-GST-NSEE----NRT----LCHQIWKSL--RDADLVVERSVEQLYDP-EAETFLADRFVR---------GTCP---KC-GTPNQAGDNCN--CGHTYSPTELIDPVST--LSGATPI-IKE-AEHLFVELEK-------------------LHDF-LSEWV----SN-S---GAL--QPETANYLKGHFLA-----------------------------------------------------------DE-LRDWDISR----PAPYFGFEIP--------------------------------------DAPGNYWYVWFDAPIGYIASTQ---QWC--D--A-----NGEDLADWWK-------------------------SDDCEVHHFI-GKDITYF-HTLFWPGMLKTA---------------G--FSLPTK----VHIHGF-LNV-NGKK-MSKSDG---TFVKAET-FLKH--------------------I--DPSALRYFYATKLSSRVED-LDLGVDEFVEKVNSDLVGKVVNLASRVG   393
Caur_3464_Caur_163848994         5  ILVAVAWPYANGPRHIGHVAGFGVPADIFARYHRLR---GNHVLMISGTDEHGTPITLVADKEGTTPQAIADRYNKI-IGDDL-YNLGLSYDIF-TRT-TTAN----HYA----VTQDIFRTL--YERGYIIRQETLGAFSA-TTGRTLPDRYIE---------GTCP---IC-GYDEARGDQCDN-CGSQLDPTDLINPRSK--VDGQPPV-FKP-TEHFFLDLPA-------------------FAEQ-LHAWI----DR-Q---THW--RPNVRNFSL-NFL------------------------------------------------------------KE-LKPRAITR----DLEW-GVPIPLPEY---------------------------------VHRDDKKIYVWFDAVIGYLSASI---EWA--RNSG-----QPDAWRTWW-------------------------QNPAARHFYFM-GKDNIVF-HTVIWPAMLLGYGAGGDFGTDPSGTYQGVPLQLPYN----VVSSEF-LTM-EGKK-FSSSRG---IVIYVND-FLSR--------------------Y--DADALRYFLTI-AGPENQD-TDFTWAEFVRRNNDELVATWGNLVNRTL   415
metG_Ctra_166154253              6  ILITSALPYANGPLHFGHITGAYLPADVYARFQRLQ---GKEVLYICGSDEYGIAITLNAELAGMGYQEYVDMYHKL-HKDTF-KKLGISVDFF-SRT-TNAY----HPA----IVQDFYRNL--QERGLVENQVTEQLYSE-EEGKFLADRYVV---------GTCP---KC-GFDRARGDECQQ-CGADYEARDLKEPRSK--LTGAALS-LRD-TEHAYLHLER-------------------MKED-LLAFV-------Q---GIYL-RPHMRNFVT-DYI------------------------------------------------------------EH-LRPRAVTR----DLSW-GIPVP--------------------------------------DLENKVFYVWFDAPIGYISGTM---DWA--ASIG-----DPEAWKKFWL-------------------------DDTVTYAQFI-GKDNTSF-HAVIFPAMEIG-------------------QSLPYKKVDALVTSEF-LLL-EGFQ-FSKSDG---NFIDMDA-FLET--------------------Y--SLDKLRYVLAA-IAPETSD-SEFSFQEFKTRCNSELVGKYGNFVNRVL   395
Haur_2785_Haur_159899304         5  ILVAVAWPYASGARHLGHVAGFGVPSDVFARYQRLV---GNNVLMVSGTDDHGTPITVRADREGKTPREVTDFYNAE-IRNNL-RDLGLSYDLF-TRT-STEN----HYQ----ITQAFFTRL--QEKGYIFAQEMIGTYSE-ADKRFLPDRYVE---------GTCP---HC-GYTKARGDQCDN-CGKQLDPVDLIEPRST--LSGATPV-FKP-TTHFFLNLPA-------------------FVER-LREWI----ES-Q---NHW--RPNVKRFSL-GLL------------------------------------------------------------DD-VPARAITR----DLTW-GVPIPVEG----------------------------------DEFDSKRIYVWFDAVIGYLSAAV---EWS--IKVG-----RPEAWRDWWL-------------------------NSDARHYYFM-GKDNIVF-HSVIWPAMLIGH---------------G-ELELPYE----VVSSEF-LTLAGGEK-ISSSRAEGNSIPFVGE-FLAQ--------------------Y--DPDPLRYFLVI-AGPETSD-TEWSLGEFIRRNNEELVATWGNLVNRVL   402
Sare_0722_Sare_159036379         4  VLAAVAWPYANGPRHIGHVSGFGVPSDVFARYMRMT---GHDVLMVSGTDEHGTPIQVQADAEGVTPRELADRYNRV-IVADL-HGLGLSYDLF-TRT-TTRN----HYA----VVQELFEGM--YRNGYIVPKTTMGAISP-STGRTLPDRYIE---------GTCP---IC-GYESARGDQCDS-CGNQLDPIDLRNPKSK--INGETPE-FIE-TEHFFLDLPA-------------------LAGV-LRQWL----DT-R---EGW--RPNVLRFSK-NLL------------------------------------------------------------DD-LQPRAITR----DLEW-GVPIPLEGW---------------------------------RDRGDKRIYVWFDAVIGYLSASI---EWA--RRSG-----DPEAWRRWWSA---------------------DGQGKDAPGYYFM-GKDNIVF-HSVIWPALLAGYSGEGSRDGQPGE-L-G-RMNLPTE----VVSSEF-LTM-EGRK-FSSSRR---VVIYVRD-FLER--------------------Y--DADALRYFIAV-AGPESND-TDFTWAEFLRRNNDELVAGWGNLVNRSI   415
Amir_0642_Amir_256374791         5  VLTAVAWPYANGPRHIGHVSGFGVPSDVFSRYMRMS---GHRVLMVSGTDEHGTPISVQAEKEGLSVRALADKYNRV-IAEDL-QGLGLSYDLF-TRT-TTGN----HYN----VTQELFLAM--WRNGYVVAKTGKGAISP-STGRTLPDRYIE---------GTCP---IC-GYDGARGDQCDS-CGNQLDPVDLKNPKSR--INGETPK-FVE-TEHLYLDLPQ-------------------FIDS-LGTWL----QT-R---SEW--RPNVLKFSQ-NLI------------------------------------------------------------GD-LRPRAITR----DLDW-GIPIPLDGW---------------------------------RDQSMKRFYVWFDAVIGYFSASV---EWA--RRSG-----NPDAWREFW--------------------------TGDAQAYYFM-GKDNIVF-HSLIWPSLLLGNNGEGAGGGEPGG-F-G-KLNLPTE----VVSSEF-LTM-SGSK-FSTSRG---NVIYVTD-FLRD--------------------F--GPDALRYFIAA-AGPENQD-TDFTWEEFVRRTNFELANEWGNLVNRSI   411
metG2_Rsol_17546537              7  YVLIPVMPTPNGPLHLGHIAGPFLKMDMLARHLRRN---GNTVALVSATDPYETHVLPRADEQNKPVEQICAENHRA-IHRCL-QALDIRYDAF-IDP-LASP----YRARLNGITREVLDDL--HAQGRLHARNEPVHISR-RTGRMLVGSRIV---------GTCP---CC-GVEMG-GYHCEG-CGMEVSPRDLIAPRAE--PADDTVE-VEA-RASVFVDADL---------------------PA-LQRRM----LE-----ARV--PADVRRIAE-RFM-----------------------------------------------------------HAAG-SAVRLSN----PGEW-GEIWPNTL-----------------------------------ATAPSVVFSYTALFMLSVLCG----EAA--R--------EILALDHNPF-----------------------DRRSDALIVTSF-GFDNTVP-FCVGVETLAQHSR----------------RYRGFDR----CLTNFF-YTL-DGRK-FSTSRQ---HCIWADQ-AVRE--------------------LGVASDVLRYFLAK-TSPESGP-SDFSRDGFDAFRRAIEPRLAQMKAAVES   400
Rcas_3011_Rcas_156742957         5  ILVAVAWPYANGPFHVGHIAGAYLPADVFARYHRLR---GHRTLMVSGSDCHGTPITIAAEREGITPQDVIRRYHPT-FLKTF-QALGISFDLF-TQT-YTDN----HYR----VTTDMFLRL--LENGYLYKETMVGSYSE-TLGRFLPDRFVE---------GTCP---NC-GYPRARGDQCDS-CGHLHDPQDLIAPRSV--LDGAPVT-FRE-TEHFFLDLAK-------------------LEPQ-LRAWI----ESVD---RSYW-RANTLLFTQ-NWL------------------------------------------------------------REGLRGRAITR----DLEW-GVPVPV----------------------------------DDPAFKDKRIYVWFDAVIGYYSASV---EWA--ERTG-----APDAWKDWWVCL--------------------PDGSAPARSYYFI-GKDNIPF-HTIIWPAMLIGY---------------G-NLALPYD----VPANEF-LNL-EGDK-MSTSRN---WALWAPE-IEDR--------------------Y--QPDAIRYYLIA-NGPETRD-SNWSWADFVQRVNSELVATWGNLANRVL   406
Caci_0586_Caci_256389800         8  ILTAVAWPYANGPRHIGHVSGFGVPSDVFSRYQRMA---GNRVLMVSGTDEHGTPILVQADKEGVTARELADRYNRV-IAEDL-QSLGLAYDLF-TRT-TTRN----HYA----VVQEIFKGL--YDNGYIFPKTTMGAISP-STGRTLPDRYIE---------GTCP---IC-GYDGARGDQCDN-CGNQLDPDRLIDPRSR--INGETPK-FIE-TEQFFLDLPA-------------------FASV-LGGWL----QQ-Q---KTW--RPNVLKFSL-NLL------------------------------------------------------------DD-LQPRAISR----DLDW-GVPVPLEGW---------------------------------VDRPDKKLYVWFDAVVGYLSASV---EWA--RRTG-----DPDAWRAFWQTG--------------------PNGEAP-DAYYFM-GKDNIVF-HSEIWPAMLLGYDGRGEKNGKPGS-L-G-ALNLPHE----VVSSEF-LTM-EGRK-FSSSRA---VVIYVRD-FLSR--------------------Y--DADALRYYITA-AGPETQD-TDFTWSEFLRRNNDELVAGWGNLVNRAI   419
metG_CPro_46446299              14  ILITSALPYANGPLHFGHIAGAYLPADCYARFQRLM---KKDVLYICGSDEYGIAITLSADLAGRTPQEHVDLFHHI-NQSFF-EQLQISFDHY-SRT-TWKG----HVE----PTHQFFNDL--LQNGYIEERTTDQLYSE-KDQKFLADRYVV---------GTCP---RC-GFENARGDECPC-CGASYEATELKNPRSK--LTDASLI-LRP-TKHWFLLLEK-------------------FKKP-LMEWL----ET-----KNW--KPNVINFIR-GYI------------------------------------------------------------DH-LHARAITR----DMKW-GISVPL------------------------------------PDSEGKVLYVWFDAPIGYISATK---EWA--LLRG-----EEKLWEKYWL-------------------------DPETKLVNFI-GKDNIPF-HASIFPAMIMG-------------------QNQPYKLVDELPANEF-YNL-EGKQ-FSKSDG---WYIDLED-FFKH--------------------Y--TSDQIRYAIAS-NAPETSD-SEFTWKDFQLRCNSDLLGKYGNLVNRVL   405
metG_Blon_23464786               4  VLVNVAWPYANGPRHIGHVAGFGVPSDVYARYERMK---GNDVLMVSGTDEHGTPILVEAEKEGLTAQELANRYNRV-IAKDL-CDLGLSYDLF-TRT-TTGN----HEH----VVQEMFKQC--LENGYIYKGTQQVAISP-STGRTLPDRYIE---------GECP---IC-HAEGARGDQCDA-CGNELDPDELINPVSK--INGETPR-FEQ-TEHYFLDLPA-------------------LAEA-NKAWL----ET-R---KGW--RTNVINFSL-GLF------------------------------------------------------------KE-VKPRAITR----DIDW-GIPVPVKGWI---------------------------------DNPNKKLYVWFDAVIGYLSASI---EWA--RRQG-----DPEKWREWWN-------------------------DPACPAYYFM-GKDNITF-HSQIWPSEMLAYNGKGSKGGETGP-M-G-PLNLPEQ----VVASEF-MTM-EGKK-FSSSRG---IVIYVKD-ILAR--------------------Y--PVDAVRYYISV-AGPESSD-SDFTWAEFVRHNNEELASSWGNLVNRVA   411
metG_Ccav_29840409               5  VLITSALPYANGPLHFGHIAGAYLPADVYARFRRLL---GDDVLYICGSDEYGIAITLNAERVGLGYQEYVSMYHKV-HKDTF-DKLGISLDFF-SRT-TNPF----HKA----IVEDFYLEL--KSKGLVENQISFQLYSE-DEKRFLADRYVE---------GTCP---KC-GFDGARGDECQK-CGADYEATDLINPRSK--LSGSQLV-LKE-TEHAYFHLER-------------------MVEP-LLAFI----E------KAYL-PEHIRKFVV-DYI------------------------------------------------------------KN-LRPRAITR----DLSW-GIPVP--------------------------------------DFPNKVFYVWFDAPIGYISATM---DWA--ASVN-----TPDLWKDFWL-------------------------EESTEYVQFI-GKDNIPF-HAAIFPAMELG-------------------QSIPYKKMNALISSEF-YLL-EGAQ-FSKSEG---NFVDMDA-FLDT--------------------Y--SLDKLRYVLAA-TAPETSD-SEFTFLDFKTRCNSELVGKFGNFINRVL   394
CC_1480_Ccre_16125727            4  ILITSALPYINGIKHLGNLAGSMLPADVYARFKRAQ---GHETLYICATDEHGTPAELAAAAAGQDVATYCAEQHVL-QHEVG-RAFGLSWDHF-GRS-SSPQ----NHR----LTQHFCQAL--EDHGLIEERVDQMVYSV-DDKRFLPDRYVE---------GTCP---HC-KFEKARGDQCDN-CGNLLDPTDLIDPYSV--ISGSRNIEVRD-TKHLYLLQTK-------------------MQDK-IRAWV----D-AH---ADW--PPLARSIAY-KHL------------------------------------------------------------DEGLIDRGITR----DLAW-GIPVAQDGV-------------------------------PRPGFEEKVFYVWFDAPIEYIAATQ---EWA--E-GS-----PDRDWKRWWRT---------------------DAGADDVRYVQFM-GKDNVAF-HTVSFPATILGS---------------EEPWKSVDM----LKAFNW-LNW-YGGK-FSTSNK---RGVFMDA-ALEI--------------------L--PPDLWRWYLTA-NSPEGSD-TAFTWEQFASAVNRDLADVLGNFVNRIL   406
Amir_2662_Amir_256376783         7  AVVIAATPTSNGDLHVGHLAGPYLSADVYARYLKAT---GRPVVYTTCTDDSQSYVVSTAHRRGLAPEELVRTSTEQ-ISRSL-AAAGTLVPGL--PP-IDER----YRR----TVLDYLLEL--HAAGRFQPRTVRLPYAK-NAGVFLYDGLVS---------GTCP---VC-LSGSC-GGACEN-CGHPNNFDELIDPKYT--IDPSDPVVYRE-QRILVLPLEE-------------------YRER-LSSYY----ASRT---PRW--RPHAKQLIG-ELL------------------------------------------------------------ARPLPDVPVTF----PGSW-GIPAPF------------------------------------PETPGQIVYPWIEAVPAAMYSTW----WAATEQGN-----PPGAADELWR------------------------AESGAELVYFH-GFDNVYH-WGLVDLVMLMAH---------------GDRYTTPDA----NVCNEF-YDL-EGEK-FSTSRG---HLIWGAD-LFAE--------------------V--PRDLVRFYLAL-TAPEFQR-TNFSREQLHSVTTRRLVDPWNSLADTTS   402
Sare_2149_Sare_159037752         9  AVIIAATPTPNGDLHLGHLAGPYLAADVYARHLRMS---GRPVVYTTCTDDSQSYVLTTARRQGVPPRRLAATAATA-IARSL-DAVGISTAGL--PP-TGDT----YRG----TVLDFVGQL--HAAGRFRQRRVRLPYAR-HAGMYLYDGLLS---------GTCP---TC-LSDSS-GGVCEA-CGHPNTFDDLLDPRYS--LDPDDPVEPRV-ADVLVLPAED-------------------YRGR-LAEYY----ARHT---PRW--RPHARRLVN-ELL------------------------------------------------------------ARPLPDIPVTV----PGSW-GIPAPFA------------------------------------QTPGQVLYPWIEAMPASIYSTW----WS--RSPRGA---TGGNIDAPWR------------------------AETDTELVYFH-GYDNVYH-WGLVDLVLLLAH---------------GDRYVLPAA----NVCNEF-YEL-AGAK-FSTSRD---HLVHAPE-VLAE--------------------V--PRDLLRFYLAL-TAPEYQR-STFDRAALPSVTQTRLVEPW-NRLSRAL   403
```

---


6. Alignment of members of the novel papain-like family
  

  

```
FINAL                                 -----------------------------------------------HHHHHHHHHHHHHH-------------------EEEEEE-------EEEEEEEE-----EEEEE-------EE-----------------------EEEHHHHHHHHHHHHHHHHHH--------HH---HHHHH--H-----------------
ALIGN                                 -----------------------------------------------HHHHHHHHHHHHHH-------------------EEEEEE-------EEEEEEEE----HHHHHH-------HE-----------------------HEHHHHHHHHHHHHHHHHHHHH-------HH---HHHHH--H-----------------
HMM                                   -----------------------------------------------HHHHHHHHHHHHH-------------------EEEEEEEE------EEEEEHHHHHH--EEEEE-------EE----------------------EEEEE-HHHHHHHHHHHHHHHHE-------HH---HHHHH--HH----------------
FREQ                                  -----------------------------------------------HHHHHHHHHHHHH--------------------EEEEEE-------EEEEEEEEE-----EEEE-------EE----------------------HHHHHHHHHHHHHHHHHHHHHH--------HH---HHHHH--HH----------------
PSSM                                  ------------------------------------------------HHHHHHHHHHHH--------------------EEEEEE--------EEEEEEE----EEEEEE-------EE-----------------------------HHHHHHHHHHHHHHH--------HH---HHHHH--------------------
FB2170_07045_Fbac_88711853            --MEKFKKRAVKYLDI-IE----FGDWKFKLYSM----KYNELRVTPEIEKTIKTILPDWIKKN---SQRND-FPN-YKIGTVIIHE-AKD-SILVVVNWWVYENVIQNHV-------YCSEYKHPYKF---VDISS---KGLRFCVWEMNILWHERNLWVKHVLKKSDNPDWNS--YLNHY--YVLDYAA-----------
Bsuib55_010100007757_Bsui_254700738   --TSCYKPRAAWFDGL-TE----CGPAAIKLSII----EADPANPVAEATVCIARRQIASAAAK---LADTP-HMG---AGFAILHQ-GEE-SLWPLLHWWLEGGIATRML-------WQCELGDEVEF----MPAQ---PLLMACVWELGIIDFERRAWMETAMAGK--PVAD---YLART--LPRGTV------------
RCCS2_11869_Rsp._126734569            DHLAPFRSRPTLHLPQ-FN----QSGWQLKRYAI----LSQGHTYNDDVTTAATAKAMRLLPKAGSLVDADG-NHG---IGFQIIHF-AQV-AVVSPVFYWQWGSVLAHAG-------QVRASWDEPTL---FHDGA---KEVLGCVWEMDIVNFEVNVWKKHMLSDN--KTPSQ--NLATY--LDQSFD--N---------
Caci_2489_Caci_256391684              GRLMSYRTPIAWHAGT-DV----IDGRAVKRYHI----ARDATPIRPDWEASANALIADMLAPPDETPPAAFTVLFR---SGAGLH-LNV--YSWYWDNVIYGKFATGGVPF------LGSPDEDPGNL----APVQ---PPVLGCVYELGVVVHERSAWIRHVLMTEK-PDMDA--YLADV--LPTGPVG-LPDVPSGLPL
Jann_3632_Jsp._89056123               GPVERYSPRRVTYLGL-WE----AGPLRMKAYGL----EARGHNVSEDVIARARTLLDHDVTSA---VRAMG-DSN-N-VGFVIIHP-GTL-GVSISSYWWVQGSVLCHHM-------VRQLYSAPQPM----ETAS---RPVIGCVWELEIVAAEQAAFKRHMMGPD--HDSDG--YVASR--IAES--------------
SPO1854_Rpom_56696730                 GTGPRYQPRQVSDLGI-RS----VGPVRFKIWGL----CAAGRDVAEEDLACAQHFLSDAVLPA---VAAQG-DSD-D-LGFVIVHP-GSA-GLSIAAQWWVQGSVLCQRL-------FRREYGAAQPV----DTTA---RPVVACVWELSIINAEQEAWRHTMMVPQ--PDPEA--YLAARGGLTAV--------------
Nhal_1255_Nhal_292491364              IIAEAYKTRPIRFLGL-WE----ISGWKLKAYGI----SYANQIPGQQLIDAAHRVTGERLSAS---AAKTH-HYG---VGFVGIHE-GKT-GNFVFVDWWADENELHHHV-------YLSPSEQPAAL---EYMTP---TGLTACAWDLFLISHERDAWVNHVLKQA--AAP----DLEG---------------------
Cflav_PD5319_BEll_223935357           MIIQPYKTRPIRFLEL-WQ----EGEWRIKVYSI----AYERLTARAELVEAAKTVAREKLATV---PSTLQ-HYS---VGFLGVHD-GRT-SNFIFVDWWAEENELHHHV-------YVSPSNDPARL---TYMTP---TGLAACVWDLRVMAFERKAWVDCVLRNY--KSP----DLEAY--LQQRLNEDV---------
Dd1591_3665_Dzea_251791231            MNPFNYQKRSVEFLGV-LS----VGRDKLKVYYL-------KSQKQPHQ-VPSREMQQAWLEQG---IGVDD-FPSDHHVGFAIFHH-ADD-GVYLLISTWCDANMMRHRV-------FSIDDAGKLHS---LEQ-----TKIIACVWELEVMFYERNAWITQVMVSE--MLEPD--NVQRY--LAEGYNGWV---------
SalbJ_010100017556_Salb_239981247     MNPLTYVPRLVTALPP-VE----AGGRTLKAYAMF---ADPERNRELPEPGWLRRHAASVLDEP---LQEED-HP----AGFLILHR-GAQ-ADYLLVSQWYDADMLRHRV-------RGAVTGADGET-VFAPLAQ---RDLVACVWELEIIKFERDAWVNTVLAHG--TLDQA--TLDAY--LGTTFSGWV---------
HMPREF0204_2543_Cgle_227368226        MKITSYPSKYAQPKGI-FT----VGKTKFKWYDL----AGDPAEIPQQDIENAQKCIDN-ADEN---FKNID-D-----LGFVIMHR-CGK-NYLLLVCTWRSENELWESV-------YYDGSGKFEVW----DRNK-T-HLPTYCVWEMGIVYHESRSWKKYLGSER--ADKDK----EAY--LRDFFEGEV---------
api65_Ypse_49658912                   MNPLNYQERAVSFLGT-QA----IGQAQLKMYYL-------TSEKQAETPVPEPELQRQWLVQG---LDEAN-FPGEHHIGFAIIHA-ADD-GDYLLISTWCDANMLRHRV-------FTIDNENRLQS---LEI-----TKIIACVWELAVMFYERTCWIEQVMTTE--KLEDG--NVQRY--LQKGYSGWV---------
Amir_4217_Amir_256378258              SNPLAYSPRLVKALPP-LE----VRGRVLKPYAMF---ADPARAAEVPDPAWLGERAAAVLGEG---LDAGD-HP----LGFLILHY-GCD-GDYLLVSQWYDANMLKHWV-------RGLTPAADGST-TTAPLAQ---RDLVACVWELEVMRFERDAWVNTVLARG--TLDDD--ARAAY--LATTFSGWV---------
yfred0001_25660_Yfre_238787777        WQPQPYLPRAITPAGS-KN----LSRWQLKCYVI----RYSQAQGAAPDYAPAYQLVRQWLPDE---AETVN-RPG---VGLVIEHQ-GKT-LNYLIVGWWDNENELRVKV-------WVQEQGIWRAA-----------RDESFCVWDLQVIAFERDAFVDTLLQPA--PDIPA--YMNRY--LTVTVD------------
yberc0001_2950_Yber_238783899         WQPQPYLPRAISPAGI-KI----LNQWRLKRYVI----RYSQAQGATPDYSPAYQLVGQWLPRD---AETVN-RPG---VGVVIEHQ-GKT-LNYLIVGWWDNENELRVKV-------WVQEQGVWRAA-----------RDESFCVWDLQVLAFERDAFVATLLQHT--PDIPA--YMNRY--LTVTVD------------
yinte0001_38410_Yint_238793201        WQPQPYLPRDITSAGV-KY----LKQWQLKRYVI----RYSQAQGDTPDYAPAYQLVGQWLPDE---AETVN-RPG---IGFVIEHQ-GKT-LNYLIVGWWDNENELRVKV-------WVQEQGVWRAA-----------RDESFCVWDLQVMAFERDAFVATLLQHT--PDIPA--YLNRY--LTVTVD------------
YE4124_Yent_123444294                 WQPQPYFPRAITPAGI-KN----LNRWQLKRYVI----RYAQAQGTAPDYVPAYQLVSQWLPLA---AETVD-RPG---VGFVIEHQ-GKT-LNYLIVGWWDNENELRVKV-------WVQEQGIWRAA-----------RDESFCVWDLQVMAFERDAFVDTLLQHT--PDIPA--YMNRY--LTITVD------------
yrohd0001_11790_Yroh_238750245        WQPQPYLPRAITPAGI-KN----LSHWQLKRYVI----RYSQAQGATPDYTSAYQLVSQWLPRD---AETAE-RPG---VGFVIEHQ-GKT-LNYLIVGWWDNENELRVNV-------WVQEQDIWRAA-----------QGESFCVWDLQVMAFERDAFVDTLLQHT--PDIPA--YMNRF--LTVTVD------------
Oant_0811_Oant_153008147              --ISAYKPRAAWFDGL-TT----CGPATIKLNII----EADPTNPVAEAAVGLARRQIETAAEK---LTALP-HLG---VGFAVLHQ-GEE-GLWLLLHWWLEGGIATEIL-------WQSELGDEVDF----MPAQ---PLLMACVWELGIIDFERRAWMETAMAGK--PVAD---YLART--LPRGTV------------
ShygA5_010100010641_Shyg_256775373    --SREHHARTARAWGT-RE----VGGHLVKVYSV----HAPGRAVTDQNVATALRLAGGHLELG----ATRG-SLG---LAVLIVHA-GGD-GDYVLIHSWIEGYMSDLAV-------FSGPADAPDEL----RPGR---VGLAPCVWEAAVLAHEREAFSRHLLDGD--GALAD--RLAAW--QADTLDGGV--------R
Bru83_010100001051_Bsp._254718132     --TSCYKPRAAWFDGL-TE----CGPAAIKLSII----EADPANPVAEAAVCIARRQIASVAAK---LADTP-HMG---AGFAILHR-GEE-SLWLLLHWWLEGGIVTRML-------WQCELGDEVEF----MPAQ---PLLMACVWELGIIDFERRAWMETAMAGK--PVAD---YLART--LPRGTV------------
MicauDRAFT_0696_Maur_270498759        ---MAFHDKELTVPGT-VE----VAGRHVKRYHM----DQPDRRLEPRVVDAAYAYLPSLLPGP------DGSTPP---ASWVVLHR-GADTGAYLLAYSWFFDNVVECRIAIAGQPALDCPDDDPAHF----VDLT---RPGVGCVWELGVLEHERTAWIRHVLAPDR-PDLAG--YLADA--RAEGPVG-R---------
Sare_2143_Sare_159037746              --PPDYRHRPKRADPG-PP--LLLPDGCLKWYDV----HDIGDGVPGSVRDAARNFVRRQAGSD-A-LDISG-----A-LGFAILHRCDGD-LYYLDVCTWRHANELWESV-------YTADPDAGGAF-RRHRSTG---HVEIGCVWELGVVRHEVQAWTAFLDSAR--DEPAK----IAY--LADQLTGQV---------
SSEG_09743_Ssvi_254405405             --ADTHHARPARPLGL-QE----AAGHLVKVYAL----EAPGRTVSAQEAEAGLRIAARHLELG----PLRG-SLG---LAVLIVHA-GGD-GDYVLVHSWIEGDMADLAI-------FAGPVGEPDAL----RPGR---AGLSPCVWEAAVLAHERDAYSRHVLDGT--GSLTD--RLTAW--GADTVTGDV--------R
Sros_3297_Sros_271964783              GPAMVFHRKQLRVPAP-AV----AAGRHVKRYHV----NLDGSEIDQARQEAAYAFLPGLLPRP------DG-TPA---ATFTVLHE-TAN-GIFLNAYSWYWDNVLYCRTAAAGIPFLGCPDEDYTHW----VELA---EPLIGCVWELPPIEHERSAWIRHVLQPAE-PDLGA--YLADL--LPEGPIG-QVC-------
BruAb1_2084_Babo_62290951             --TSCYKPRAAWFDGL-TE----CGPAAIKLSII----EADPANPVAEATVCIARRQIASAAAK---LADTP-HMG---AGFAILHQ-GEE-SLWLLLHWWLEGGIATRML-------WQCELGDEVEF----MPAQ---PLLMACVWELGIIDFERRAWMETAMAGK--PVAD---YLART--LPRGTV------------
BcetM6_010100009074_Bcet_254713489    --TSCYKPRAAWFDGL-TE----CGPAAIKLSII----EADPANPVAEATVCIARRQIASAAAK---LADTP-HMG---AGFAILHQ-GEE-SLWLLLHWWLEGGIATRML-------WQCELGDEVEF----MPAQ---PLLMACVWELGIVDFERRAWMETAMAGK--PVAD---YLART--LPRGTV------------
Adeh_3424_Adeh_86159843               --VRPPTPPVTRYRGL-VE----HLGFRLKRYAS----VDAGSAFAEERFRSGRGLALSALPEP---AVQHD-RPG---VGLVLEHQ-LPE-LDRVTVAWWDRERELPLRV-------IVGDDAGWRPA---------R-EGESLCVWDLAIVAAERDAYVETVLTPGG-GGIDA--YLARS--WDGALRG-----------
FuraDRAFT_3257_Lnit_224826653         FRPTPYQPRPLAARGVLAA----PGGWRLKLYDI----AYDALPLDEARFAIGVDGALAGLPQP---ALTAQ-RPG---VGALIRHA-GRG-MDYLVLVYWDNDNECLVRV-------WVRDENGWRAA-----------RAESFCVWDLQVIWHEREVCVRHLLVDT--PDMDG--YLADT--L-----------------
AnaeK_3505_Asp._197123896             --ARPPTPPVTRYRGL-VE----HLGFRLKRYAS----VEAGEPFAEERFRSGRGLALSALPEP---AVQHD-RPG---VGLVLEHQ-LPE-LDRVTIAWWDRERELPLRV-------IVGDDAGWRPA---------R-EGESLCVWDLAIVAAERDAYLETVLTPGG-GGIDA--YLARA--WDGALRG-----------
Snas_3175_Snas_291300661              ---MTYTQRYMTHLPP-VI----IAGRTIKRYHL----TPDTTPIPAHIQTAAYRHLPRLLPDH---SGPTP-----P-ATVAILHQ-DKDEAIHLNTYTWTSEHHLA----------HARATKNPRGF----TTTP---PPATGCLWELPALVHERSAWARHMVNTTY-PSFDD--YLADI--LAGVPIA---A-------
A2cp1_3573_Adeh_220918665             --ARPPTPPVTRYRGL-VE----HLGFRLKRYAS----VEAGEPFAEERFRSGRGLALSALPEP---AVQHD-RPG---VGLVLEHQ-LPE-LDRVTIAWWDRERELPLRV-------IVGDDAGWRPA---------R-EGESLCVWDLAIVAAERDAYLETVLTPGG-GGIDA--YLARA--WDGALRA-----------
Amir_2656_Amir_256376777              --APEYRHYPKEVVPL-PDHDLVLPDAHLKWYEV----RKSDATVPDALRAEAHEFLLARVADR-D-LDLSG-----D-LGFVVHHLCGEA-FHFLIACTWRNNNEMWLSV-------YARDAARQDSF-ERVEQGR---HLQVVCVWEMGAVLHEQRAWIEYLKSPR--DEAAT----RAY--LDDRFAGAV---------
VF_A0788_Vfis_59713971                ----MYQPRTIESDVH-----W-IDIDGIKIYTI----SAENKPINISMFNKRLATVKSQSEVN-W-------RET---AAFAIYHD-GEN-YKYLVLAWWGNDNELFTSV-------SVKIKHKWLID---------P-KQYSFCLYDMEVMWRERNIYIETMDCEV--PSLIG--YRVSR--------------------
VFMJ11_A0915_Vfis_197337819           ----MYQPRTIESDVH-----W-IDIDGIKIYTI----SAINKPINISMFNKRLATVKSQSEVN-W-------RET---AAFAIYHD-GEN-YKYLVLAWWGNDNELFTSV-------SVKIKHKWVID---------P-KQYSFCLYDMEVMWRERNIYIETMDCEV--PSLIG--YRVSR--------------------
Caci_1492_Caci_256390692              LGGVPYTPRKVRPLGL-LQ----IDDWRVKLHGL-S--ATPSGDLDDAAHEGARKAAVEALPRP---GIGGG-RYG---VGFVIAHR-STN-AYSYVVGWWAYGCLLSTAA-------YSARFSEPAEI----ARCP---ARQAGCVWELAVIDHERRAWTRTMLG----TKADG--DVEGY--LGAVLSGRV---------
JNB_09059_Jsp._84494936               --MVDVESRPVEWRGV-AQ----LGPCEMKVYAM----SVADSECPDELDRAAQALTVEFAEST-P-------GSG---VGYLLLHR-ARP-AHFALAHVWDGVDLIQAY--------WTSPLDLPAEL----RPHR---AGAVGCVWELEVLAFESAQCAASFEAA---DRRER--YLSTH--L-----------------
RSp1437_Rsol_17549656                 --MQAYESREVRTRGP-FT----VNDVALKAYEIFAPGRGDDVRPEPSAFASHLRDAGDLFRSG---SEQFG-H-G---AGFVIAHY-ARD-GNYLLASRWCGLNMLRHRV-------FTFA---WKDSPAAIELAPLSMPDIIACVWELEVIKFERDQWVRTAMRDA--SGAPGAEALERY--LGAAFAGAV---------
VSAL_II0560_Asal_209809348            ----MYKLRKLEGEQD-----W-LDVDGVKIYTI----SANEEPINMVAFHQRLTEVKSEITLE-W-------DQT---AAFVIFHN-GAS-CKYLVLACWGNDNELFTSV-------SVQVNGVWVVD---------P-TKYSFCLYDIEVMWKERNIYIETIDCES--PSLVK--YRTSR--------------------
Kfla_2247_Kfla_284030191              -------------------MPWRFAHRLVKPYVI----TARGRSWDDAMVATARSAATRQLEFD----DAMG-GLG---LGVVVLHL-ADD-GTRMVVQSWAKNFQSRLSM----F--SGLDVHDLRPA---------P-IGAAPSVWELEVLSHERSSYVHHILNAE--VDVDG--WLEDA--LDTRPAPKL-D-GIPSGT
BR2109_Bsui_23502957                  ---------------------------------------------MAEATVCIARRQIASAAAK---LADTP-HMG---AGFAILHQ-GEE-SLWLLLHWWLEGGIATRML-------WQCELGDEVEF----MPAQ---PLLMACVWELGIIDFERRAWMETAMAGK--PVAD---YLART--LPRGTV------------
SCH4B_0058_Sisp_259414992             --------------------------------------------MTSEMIETAEVFLREEVIRD---AENMG-DNN-G-LGFVIIHP-GDL-GVTIAAHWWAQGSVLCQRI-------YRRQYNDAGPL----DTIS---RPAVGCVWELEIIAAKHRIGRDTMMKLQ--HDKSA--YLRT---LAT---------------
consensus/100%                        ................................................................................s....H..............h............................................sha-h..h..c.......h......................................
consensus/95%                         ............................hK..............................h..................huhh..H.........h....W............................................Cla-h.hh..Epp.h...h......................................
consensus/90%                         ............................hK..............................h..................huhhl.H..s......h.h..W.....h......................................ClW-h.lh..Eppha.pph......................................
consensus/85%                         ..............s.............hK...h..........................h..................huhhl.H..s.....bl.h..W.....h...h.......b..........................CVW-h.lh..Eppha.pphb.....s.............b.................
consensus/80%                         .....a..+.....s........hs...lK.a.h.................s........h..............s...huhhlbH..u.p...blhh..W.p.s.h...l.......h.........h................CVW-l.lh..ERpha.cphh.....ss.s....h.....h.................
consensus/75%                         .....ap.+.h...s........hs...lK.Y.l...........s.....s....h...h..........s.p.s...hGhhlhH..u.p...hlhl..W.p.sbh...l.......h.........h.............b..CVW-L.ll.aERpuahcphh.....ss.s....h.....hs................
consensus/70%                         ....sapsR.hp..s........hs...lK.Y.l......ss...s....ssh.pbh.p.ls.s.......s.p.s...hGahIhH..upp...alhl..W.p.sbl...l.......hs...s....h.............b..CVW-L.ll.aERpAahcphhs....ss.s...hlsp...Ls.s..............
```


---


7. Alignment of members of the YqcI/YcgG family
  

  

```
FINAL                                                   ---HHHH--H----------------H-H------------------------------------------------------------HHHHHHHHHHH---------------HHHHHHHH---EEEEE-----------HHHHHHHHHHHHHHHHH-H----------------EEEEEE------------HHHHHHHHHHHHHHHHHHH----------------------EEEE----EEEEEE---------------------EEEEE--HHHHH--------HHH-HHHHHHHHHHHHHH----------------------EEEEEE-------------------------
ALIGN                                                   --------------------------H-H------------------------------------------------------------HHHHHHHHHHH-----------------HHHHHH---EEEEE------------HH-HHHHHHHHHHHHH----------EE------EEEEEE------------HHHHHHHHHHHHHHHHHH-----------------------EEEE----EEEEE-----H--HH------------EEEEE--HHHHH--H---------HHHHHHHHHHHHH---------------------HHHHHHH--------------------------
HMM                                                     ---HHHH--HHHH----------H--H-H------------------------------------------------------------HHHHHHHHHH--------------EEEEHHHHH---EEEEEE--------HHHHHHHHHHHHHHHHHHHH-H---------E------EEEEEE-----------HHHHHHHHHHHHHHHHHHHHH----------------------EEE----EEEEEEE-HHH--HHHH----------EEEEEHHHHHHH--HH----HHH-HHHHHHHHHHHHHH------HHHH-----------EEEEEEE-------------------------
FREQ                                                    ---EE---------------------H-H------------------------------------------------------------HHHHHHHHHH----------------HHHHHHH----EEEE-------------HHHHHHHHHHHHHHHH---------EEE------EEEEEE-------------HHHHHHHHHHHHHHHH------------------------EEEE-----EEEE-----------------------EEEE----EE-------------HHHHHHHHHHHHHH--------------------H---HEEE-------------------------
PSSM                                                    -----HH--HH-----------------H------------------------------------------------------------HHHHHHHHHHH---------------H-HHHHHH---EEEEE-----------HHHHHHHHHHHHHHHH-------------------EEEEEE-----------HHHHHHHHHHHHHHHHHHHH-----------------------EEE-----EEEEE---------------------EEEEE--HHHHH--------HHH-HHHHHHHHHHHHHH-----------------------EEEE--------------------------
csA_Slav_288779623                                      A-ELISQ--EQIGDD-------LN--G-W------------------------------------------------------------HRDAFEDIASRL-TDP-----GFPCVFSRNAFRKKLVKFVFVE-----GSGKEDIRHLGAGLKDYVELSR-D-WDGALDTAY------PLVVVFSADAVT-----ADSVEQYHAFGWWVLQELHAIDP---TPWPEG-VDKGPQSEAWSMCFHGMPLFINMSSPAH--QVRRSRN---LGRHFALVINPRERFD--VFAGDTPSG-RKVRSNIRGRIARYDGTPHAQQLGS-YG-TGA--LEWMQYGLVE-ENR-E-RADVCPFT---F-RGA-----------
StAA4_010100013175_Ssp._256668100                       T-TLITQ--QEAETS-------GN--D-W------------------------------------------------------------RARAFRDVESKL-TDR-----DFPCVFSRNAFRKRLLLFAFAE-----DAGPDAIAKLGAELADYVELSR-N-WNGHLDTAY------PLLIVFSPAAVS-----AGSVAGYQAFGWKVIQKLHHVDP---APWPEN-VGTDPGESSWSMCFNGMPLFFNMSSPAH--EVRRSRN---LGEHFVLVVNPRERFD--AVAGDTPSG-RKVRSNIRARIDRYDGAPRARQLGS-YG-VAG--LEWWQYGLPE-EDV-D-RTDTCPFS---F-RAAEIA------Q-
Csal_1088_Csal_92113215                                 N-RLIEQ--SDIENM-------PN--T-W------------------------------------------------------------KKQAYRQIAKKLGPKS-----NFPCIFSKNAYKKGLLQFVFME-----SISHDQMEELKVCLTEFIEANK-E-WDHRISTAK------PLLIVFSTQAIK-----SERLEDYHSFGWNVLRSIHALDP---SPWPHG-VDKDPSSPNWSMCFNGMQLFINMSCPAH--KVRRSRN---LGDHFIMVVNPRERFD--VVAGDNAKG-HRVRDEIRGRIEIYDNCKPCKQLGY-FG-SDT--MEWQQYGIIE-ENK-T-RTSKCPYK---I-D-------------
ROS217_14866_Rsp._85704290                              N-HI--S--VADVEDHY-P---EQ--S-W------------------------------------------------------------QRLVFADLKSTL-TSTSR---PFPCVFGASAIKSRQLRVAFV------DPLTPD--HLGPILRDYITHAR-D-YG-R---MT------SLVVF-ARPGPV-RD-----MKSYEDQLWTLLDGMERTDT---TPRPPA-IPAEIDHPNWEFCYAGEPIFVSCATPAH--VLRASRR---S-ATFMAVFQPRWIFK--GIMDSDEPAVQRSLHNIRDRIMAYDAVPVFPYLGS-YGNPEA--REHVQYFIYD-TNE----RPACPFH-----QL---G------Q-
bcere0025_14610_Bcer_229069256                          K-SYLLD--NEGMRTRT-D---IP--D-W------------------------------------------------------------VAKEFEIFSNVV-LEP-----TFPCYFGLTALKKNELRYSFLS------HNDWS--HLPHTMLSFLELMK-E----RPIVRR------GFFLFVEPECEE----QS--LEYYRDSFWKVLQYLHENDN---QAWPKQ-IPEDPDHYLWEFSFGGEPIFAFGNAPAY--KQRKTRH---LGNSLVIGFQPRTIFD--GLEGDRPKG-AYSRQMVRERVEKWDQLPKHPNISH-YGDPEH--REWKQYFIGD-DVE-P-IKGKCPFL---H-RI---E------K-
bthur0006_14950_Bthu_228952084                          K-SYLLD--NEEMRTRT-D---IP--D-W------------------------------------------------------------VAKEFEIFSSVV-LEP-----TFPCYFGLTALKKNELRYSFLS------HNDWS--HLPHTMLSFLELMK-E----RPIVRR------GFFLFVEPECEE----QS--LEYYRDSFWKVLQYLHENDN---QAWPKQ-IPEDPDHYLWEFSFGGEPIFAFGNAPAY--KQRKTRH---LGNSLVIGFQPRTIFD--GLEGDRPKG-AYSRQMVRERVEKWDQLPKHPNISH-YGDPEH--REWKQYFIGD-DVE-L-IKGKCPFL---H-RI---E------K-
bthur0013_15060_Bthu_228907339                          K-SYLLD--NEGMRTRT-D---IP--N-W------------------------------------------------------------VAKEFEIFSSVV-LEP-----TFPCYFGLTALKKNELRYSFLS------HNDWS--HLPHTMLSFLELMK-E----RPIVRR------GFFLFVEPQCEE----QS--LEYYRDSFWKVLQYLHENDN---QAWPKQ-IPEDPDHYLWEFSFGGEPIFAFGNAPAY--KQRKTRH---LGNSLVIGFQPRTIFD--GLEGDRPKG-AYSRQMVRERVEKWDQLPKHPNISH-YGDPEH--REWKQYFIGD-DVE-P-IKGKCPFL---H-RI---E------K-
BCE_G9241_1639_Bcer_47566081                            E-SYLLD--NEGMRTRT-D---IP--N-W------------------------------------------------------------VAKEFQNFSSVV-LEP-----TFPCYFGLTALKKNELRYSFLS------HTDWS--HLPHTMLSFLELMK-E----RPIVRR------GFFLFVEPECEE----QS--LEYYRDYFWKVLQYLHENDD---QTWPKQ-IPEDPDHYLWEFSFGGEPIFAFGNAPAY--KQRKTRH---LGNSLVIGFQPRTIFD--GLEGDRPKG-AYSRQTVRERVEKWDQLPKHPNISH-YGDPNH--REWKQYFIGD-DIE-P-IKGKCPFL---H-KI---E------K-
bcere0020_14360_Bcer_229096188                          E-SYLLD--NEGMRMRT-D---IP--N-W------------------------------------------------------------VAKEFQNFSSVV-LDA-----TFPCYFGLTALKKNELRYSFLS------HNDWS--HLPHTMLSFLELMK-E----RPIVRR------GFFLFVEPECEE----QS--LEYYRDYFWKVLQYLHEHDN---QTWPQQ-VPEDPNHYLWEFSFGGEPIFAFGNAPAY--KQRKTRH---LGNSLVIGFQPRTIFD--GLEGDRPKG-AYSRQMVRERVEKWDQLPKHPNISH-YGDPDH--REWKQYFIGD-DIE-P-IKGKCPFH---H-KV---E------K-
bcere0019_14600_Bcer_229102299                          K-SYLLD--NEGMRTRT-D---IP--N-W------------------------------------------------------------VAKEFQNFSSIV-LEA-----TFPCYFGLTALKKNELRYSFLS------HNDWS--HLPHTMLSFLELMK-E----RPIVRR------GFFLFVEPECEE----QS--LEYYRDYFWKVLQYLHEHDN---QTWPQQ-VPEDPNHYLWEFSFGGEPIFAFGNAPAY--KQRKTRH---LGNSLVIGFQPRTIFD--GLEGDRPKG-AYSRQMVRERVEKWDQLPKHPNISH-YGDPDH--REWKQYFIGD-DIE-P-IKGKCPFH---H-KV---E------K-
bthur0009_14560_Bthu_228932985                          K-SYLLD--NEGMRMRT-D---IP--N-W------------------------------------------------------------VAKEFKNFSNVV-LEP-----TFPCYFGLTALKKNELRYSFLS------HNDWS--HLPHTMLSFLELMK-E----RPIVRR------GFFLFVEPECEE----QS--IEYYRDYFWKVLQYLHENDD---QTWPKQ-IPEDPDHYLWEFSFGGEPIFAFGNAPAY--KQRKTRH---LGNSLVIGFQPRTIFD--GLEGDRPKG-AYSRQTVRDRVEKWDQFPKHPNISH-YGDPEH--REWKQYFIGD-DVE-P-IKGKCPFL---H-KI---E------K-
bcere0002_14610_Bcer_229189785                          K-SYLLD--NEGMRTRT-D---IP--D-W------------------------------------------------------------VAKEFEIFSNVV-LEP-----TFPCYFGLTALKKNELRYSFLS------HNDWS--HLPHTMLSFLELMK-E----RPIIRR------GFFLFVEPECEE----RS--LEYYRDSFWKVLQYLHENDN---QAWPKQ-IPEDPDHYLWEFSFGGEPIFAFGNAPAY--KQRKTRH---LGNSLVIGFQPRTIFD--GLEGDRPKG-AYSRQMVRERVEKWDQLPKHPNISH-YGDPEH--REWKQYFIGD-DVK-P-IKEKCPFL---H-RI---E------K-
BCAH1134_1659_Bcer_206970670                            K-SYLLD--NEGMRTRT-D---IP--D-W------------------------------------------------------------VAKEFEIFSNVV-LEP-----TFPCYFGLTALKKNELRYSFLS------HNDWS--HLPHTMLSFLDLMK-E----RPIVRR------GFFLFVEPECEE----RS--LEYYRDSFWKVLQYLHENDN---QAWPKQ-IPEDPDHYLWEFSFGGEPIFAFGNAPAY--KQRKTRH---LGNSLVIGFQPRTIFD--GLEGDRPKG-TYSRQMVRERVEKWDQLPKHPNISH-YGDPEH--REWKQYFIGD-DVE-P-IKGKCPFI---H-RI---E------K-
BC059799_1634_Bcer_196039132                            K-SYLLD--NEGMRMRT-D---IP--N-W------------------------------------------------------------VAKEFKNFSNVV-LEP-----TFPCYFGLTALKKNELRYSFLS------HNDWS--HLPNTMLSFLELMK-E----RPVVRR------GFFLFVEPECEE----QS--IEYYRDYFWKVLQYLHENDD---QTWPKQ-IPEDPDHYLWEFSFGGEPIFAFGNAPAY--KQRKTRH---LGNSLVIGFQPRTIFD--ELEGDRPKG-AYSRQTVRDRVEKWDQLPKHPNISH-YGDPEH--REWKQYFIGD-DVE-P-IKGKCPFF---H-KI---E------K-
BCE_1721_Bcer_42780795                                  E-SYLLD--NEAMRTRN-D---IP--N-W------------------------------------------------------------VAKEFQNFSSVV-LEP-----TFPCYFGLTALKKNELRYSFLS------HNDWS--HLPHTMLSFLELMK-E----RPIVRR------GFFLFVEPECEE----QS--LEYYRDYFWKVLQYLHENDK---ETWPKQ-IPEDPDHYLWEFSFGGEPIFAFGNAPAY--KQRKTRH---LGNSLVIGFQPRTIFD--GLEGDRPKG-AYSRQTVRERVEKWDQLPKHPNISH-YGDPDH--REWKQYFIGD-DVE-P-LKGKCPFL---H-KI---E------K-
BCH308197_1640_Bcer_206974748                           E-SYLLD--NEGMRMRT-D---IP--N-W------------------------------------------------------------VAKEFQNFSSVV-LEP-----TFPCYFGLTALKKNELRYSFLS------HNDWS--HLPHTMLSFLELMK-E----RPIVRR------GFFLFVEPECEE----QS--LEYYRDYFWKVLQYLHENDK---ETWPKQ-IAKDPDHYLWEFSFGGEPIFAFGNAPAY--KQRKTRH---LGNSLVIGFQPRTIFD--GLEGDRPKG-AYSRQTVRERVEKWDQLPKHPNISH-YGDPNH--REWKQYFIGD-DIE-P-IKGKCPFL---H-KI---E------K-
BCB4264_A1667_Bcer_218235713                            K-SYLLD--NEGMRTRT-D---IP--D-W------------------------------------------------------------VAKEFEIFSNVV-LEP-----TFPCYFGLTALKKNELRYSFLS------HNDWS--HLPHTMLSFLELMK-E----RPIIRR------GFFLFVEPECEE----QP--LEYYRDSFWKVLQYLHENDN---QAWPKQ-IPEDPDHYLWEFSFGGEPIFAFGNAPAY--KQRKTRH---LGNSLVIGFQPRTIFD--GLEGDRPKG-AYSRQMVRERVEKWDQLPKHPNISH-YGDPEH--REWKQYFIGD-DVE-P-IKGKCPFL---H-RI---E------K-
bcere0027_15040_Bcer_229043456                          K-SYLLD--NEGMRTRT-D---IP--D-W------------------------------------------------------------VAKEFEIFSNVV-LEP-----TFPCYFGLTALKKNELRYSFLS------HNDWS--HLPHTMLSFLELMK-E----RPIVRR------GFFLFVEPECEE----QP--LEYYRDSFWKVLQYLHENDN---QAWPKQ-IPEDPDHYLWEFSFGGEPIFAFGNAPAY--KQRKTRH---LGNSLVIGFQPRTIFD--GLEGDRPKG-AYSRQMVRERVEKWDQLPKHPNISH-YGDPEH--REWKQYFIGD-DVE-P-IKGKCPFL---H-RI---E------K-
BCQ_1662_Bcer_222095322                                 E-SYLLD--NEGMRMRT-D---IP--N-W------------------------------------------------------------VAKEFQNFSSVV-LEP-----TFPCYFGLTALKKNELRYSFLS------HNDWS--HLPYTMLSFLELMK-E----RPIVRR------GFFLFVEPECEE----QS--LEYYRDYFWKVLQYLHENDK---ETWPKQ-IPKDPDHYLWEFSFGGEPIFAFGNAPAY--KQRKTRH---LGNSLVIGFQPRTIFD--GLEGDRPKG-AYSRQTVRERVEKWDQLPKHPNISH-YGDPNH--REWKQYFIGD-DIE-P-IKGKCPFL---Y-KI---E------K-
bcere0017_14380_Bcer_229115143                          E-SYLLD--NEGMRMRT-D---IP--N-W------------------------------------------------------------VAKEFQNFSSVV-LDA-----TFPCYFGLTALKKNELRYSFLS------HNDWS--HLPHTMLSFLELMK-E----RPIVRR------GFFLFVEPECEE----QS--LEYYRDYFWKVLQYLHEHDN---QTWPQQ-VPEDPNHYLWEFSFGGEPIFAFGNAPAY--KQRKTRH---LGNSLVIGFQPRTIFD--GLEGDRPKG-AYSRQMVRERVEKWDQLPKHPNISH-YGDPDH--REWKQYFIGD-DIE-P-IKEKCPFH---H-KV---E------K-
bthur0008_14760_Bthu_228938815                          K-SYLLD--NEGIRTRT-D---IP--D-W------------------------------------------------------------VAKEFEIFSSVV-LEP-----TFPCYFGLTALKKNELRYSFLS------HNDWS--HLPHTMLSFLELMK-E----RPIVRR------GFFLFIEPECEE----QS--LEYYRDYFWKVLQYLHENDD---QAWPKQ-IPEEPDHYLWEFSFGGEPIFSFGNAPAY--KQRKTRH---LGNALVIGFQPRTIFD--GLEGDRPKG-AYSRQMVRERVEKWDQLPKHPNISH-YGDPEH--REWKQYFIGD-DVE-P-IKGKCPFL---H-KI---K------K-
BC1604_Bcer_30019751                                    K-SYLLD--NEGMRTRT-D---IP--D-W------------------------------------------------------------VAKEFEIFSNVV-LEP-----TFPCYFGLTALKKNELRYSFLS------HNDWS--HLPHTMLSFLELMK-E----RPIVRR------GFFLFVEPECEE----QP--LEYYRDSFWKVLQYLHENDN---QAWPKQ-IPEDPDHYLWEFSFGGEPIFAFGNAPAY--KQRKTRH---LGNSLVIGFQPRTIFD--GLEGDRPKG-AYSRQMVRERVEKWDQLPKHPNISH-YGDPEH--REWKQYFIGD-DVE-P-IKGKCPFI---H-RI---E------K-
bcere0001_14730_Bcer_229195911                          E-FYLLD--NEGMRMRN-D---IP--N-W------------------------------------------------------------VAKEFENFSNVV-LEP-----TFPCYFGLTALKKNELRYSFLS------HNDWS--HLPHTMLSFLELMK-E----RPIVRR------GFFLFVEPECEE----QS--LEYYRDYFWKVLQYLHENDK---ETWPKQ-IPKDPDHYLWEFSFGGEPIFAFGNAPAY--KQRKTRH---LGNSLVIGFQPRTIFD--GLEGDRPKG-AYSRQTVRERVEKWDQLPKHPNISH-YGDPDH--REWKQYFIGD-DVE-P-LKGKCPFL---H-KI---E------K-
bpmyx0001_14760_Bpse_228990714                          K-SYLLD--NQEIKTRS-D---IP--S-W------------------------------------------------------------VSKEFENFSNVV-LEP-----TFPCYFGLTALKKNELRYSFLS------QDDWS--HLPKTLFSFLQLMN-E----RPVVRR------GFFLFVEPECEE----RS--LEYYRMYFWRVLQYLHEKDE---QPWPEQ-IPKNPDHYLWEFSFGGEPMFAFGNAPAY--KQRKTRH---LGNSLIIGFQPRIIFD--GLEGDRPKG-AYSRQTVRERVEKWDQLPKHPNISH-YGDPNH--QEWKQYFIGD-DIE-P-IEGKCPFH---H-KV---L------K-
RBTH_06262_Bthu_75759682                                R-SYLLD--NEGMRTRT-D---IP--D-W------------------------------------------------------------VAKEFEIFSSVV-LEP-----TFPCYFGLTALKKNELRYSFLS------HNDWS--HLPHTMLSFLELMK-E----RPIVRR------GFFLFVEPECEE----RS--LEYYRDSFWKVLQYLHENDN---QAWPKQ-IPEDPDHYLWEFSFCGEPIFAFGNAPAY--KQRKTRH---LGNSLVIGFQPRTIFD--GLEGDRPKG-AYSRQMVRERVEKWDQLPKHPNISH-YGDPEH--REWKQYFIGD-DVE-P-IKGKCPFL---H-RI---E------K-
bthur0012_15110_Bthu_228914279                          K-SYLLD--NEGMRTRT-D---IP--N-W------------------------------------------------------------VAKEFENFSNVI-LEP-----TFPCYFGVTALKKNELRYSFLS------HNDWS--HLPNTMLSFLELMK-E----RPVVRR------GFFLFVEPECEE----QS--TEYYRDYFWKVLQYLHENDD---QTWPKQ-IPEDPDHYLWEFSFGGEPIFAFGNAPAY--KQRKTRH---LGNSLVIGFQPRTIFG--GLEGDRPKG-AYSRQTVRDRVEKWDQLPKHHNISH-YGDPEH--REWKQYFIGD-DVE-P-IKGKCPFL---H-KI---E------K-
bcere0022_13370_Bcer_229084703                          Q-SYLLD--NEGMKNRI-D---IP--R-W------------------------------------------------------------VSQEFANFSNVV-LDP-----TFPCYFGLTALKKNELRYSFLS------HDDWS--HLPQTMQSFLELMK-E----RPVVRR------GFFLFVEPEREE----CS--IEHYRAYFWRVLQYLHEKDE---QLWPKQ-IPKNPDHHLWEFSFGREPMFAFGNAPAY--KQRKTRH---LGSSLIIGFQPRIIFD--GLEGDRPKG-AYSRQMVRERVERWDQLPKHPNISH-YGDPNH--REWKQYFIGD-DIE-P-IEGKCPFH---H-KI---E------K-
B14911_19640_Bsp._89099424                              Q-KSLLT--KEDMTNPD-I---VP--A-W------------------------------------------------------------VIEEYKTFHQTV-TDK-----TFPCYFGMTAENRGELRYAYIS------QEDWS--QLPKALEQFIDLFD-A----PKLIRH------GLFVFVEPEKEE----KD--IPYYREYFWNILKYLHDQDE---KPWPKD-DPEDPDHHLWAFSFAEEPFFVFGNAPAY--KQRKTRD---LGNSLVLGFQPRRIFE--GLEGTSKGG-IMSREKVRERVEKWDGLPKHPNISH-YGDPEH--REWKQYFIGD-DIK-P-IEGKCPFH---S-K-------------
BSG1_13901_Bsp._149181618                               E-NRLLT--KEDMTNPE-K---VP--Q-W------------------------------------------------------------VIEEYKTFRDTV-TDK-----TFPCYFGMSGEKKGELRYSYIT------HDDWS--SLPQTLEQFIGLFD-S----PKLIRH------GLFLFVEPEKEE----KD--IPYYREYFWNILQFLHEKDT---KPWPKD-YPEDPDHHLWAFSFAEEPYFVFGNAPAY--KQRKTRD---LGNSLVLGFQPRRIFE--GLEGTSKGG-VMSREKVRERVEKWDNLPKHPNISH-YGDPEH--REWKQYFIGD-DVE-P-ITGKCPFH---H-K-------------
Bcoam_010100003239_Bcoa_205372496                       K-SYLLT--KEDMTKEG-R---VP--E-W------------------------------------------------------------VKKEYLNFREVV-TNK-----TFPCYFGMAAEKKGELRYSYVS------KDDWS--TLPGAIKEFNQLFDEN----KRLIRH------GLFVFIEPETEE----KD--IPYYREYFWNILQYLHDQDE---ESWPAD-YPTDPEHHLWAFSFAGEPYFVFGNAPAY--KQRKTRD---LGNSLVLGFQPRRIFE--GLEGTSPGG-VMSREKVRERVEKWDNLPKHPNISH-YGDPDH--REWKQYFIGD-DIK-P-IEGKCPFH---H-RT---S--------
bglu_2g09820_Bglu_238024389                             ---------AGRALGAAAN---PP--A-W------------------------------------------------------------LDASYQTLRKQV-MHPD-----YPCFFGTMAEKRGEMFYAYV------NGKDIS--QLPATMQTFAELAL-R-PEYR---KN------NIAVFFEPDPEP-LSHEA-----YQQHFWQILQYLHDVDP---DPQADA-QP-DPSDGDWEFSFAGVETFVVCACPSF--RARHSRN---LGPGMVLLFQPRSVFV--DTITNKVIG-REARNQVRKRLETWDEVAAHPDLGF-YGDPGN--LEWKQYFLDD-ANQ-P-ADDRCPFL---K-RQ---R------Q-
Bcoam_010100021494_Bcoa_205375555                       N-KTLLT--KEDMINQA-V---VP--D-W------------------------------------------------------------VIREYTTFHQIV-TDK-----TFPCYFGMKAEMKGELRYAYIT------QDDWS--NLPKAVEEFLTLFK-E----PTYIRH------GLFVFVEPEEEK----DN--LEHYRKRFWDILQNLHENDK---QKWPED-KPKDPDHYLWDFHFGGEPIFVFGNAPAY--KQRKTRD---LGNSLILGFQPRKIFE--GLEGTQKGG-IMSREKVRERVEKWDNLPTHPDISH-FGDPTH--NEWKQFFIGD-DIE-P-ILGKCPFL---H-KA---L--------
Bant_01002245_Bant_65318969                             -----------------------------------------------------------------------------------------MAKEFENFSXXI-LEP-----TFPCYFGVTALKKNELRYSFLS------HNDWS--HLPNTMLSFLELMK-E----RPVVRR------GFFLFVEPECEE----QS--IEYYRDYFWKVLQYLHENDD---XTWPKQ-IPEDPDHYLWEFSFGGEPIFAFGNAPAY--KQRKTRH---LGNSLVIGFQPRTIFG--GLEGDRPKG-AYSRQTVRDRVEKWDQLPKHPNISH-YGDPEH--REWKQYFIGD-DVE-P-IKGKCPFL---H-KI---E------K-
BpOF4_14250_Bpse_288555856                              S-NYLLT--SEHMQNEK-I---VP--K-W------------------------------------------------------------VSEEYHKYREVV-MKP-----TFPCFFGRTGEERGELRYSYIS------HSDWS--HLPRTLSTFLQLVK-E----PPLVRR------GLFIFVEPENKE----QP--LSYYRNYFWDVLQYLNRHDP---KKWPEH-TPADPNDFLWSFCFDDESIFAFGNAPAY--KQRITRN---LGESMVIGIQPRRIFE--GLKGTEPNG-INSREAVRKRVEAWDKLPKHPDISH-YGDPAH--HEWKQFFIGD-DCE-P-ITSKCPFH---AVNT---N------K-
bthur0014_14910_Bthu_228900285                          ----------------------------------------------------------------------------------------------------M-LEP-----TFPCYFGLTALKKNELRYSFLS------HNDWS--HLPHTMLSFLELMK-E----RPIVRR------GFFLFVEPECEE----RS--LEYYRDSFWKVLQYLHENDN---QAWPKQ-IPEDPDHYLWEFSFCGEPIFAFGNAPAY--KQRKTRH---LGNSLVIGFQPRTIFD--GLEGDRPKG-AYSRQMVRERVEKWDQLPKHPNISH-YGDPEH--REWKQYFIGD-DVE-P-IKGKCPFL---H-RI---E------K-
BMQ_2540_Bmeg_294499296                                 S-KMLFR--ANEI-LESTH---VP--F-W------------------------------------------------------------GKDAFQYFQSDI-LSEEN---PFPCILGVEGFKKDLLRFSFVT--TPYNYQDIR--NAASALREYIDTFK-T-IG-R---YT------SFVLFFKPEHKE-RSMEE-----YETMFWDTLTFLHQIDQ---KKWPQD-IPKDPKDPLWEFCFHGEPIFVVCNTPAH--SLRKSRK---S-RGFMITFQPRWVFE--GMTGDSKIG-RHVQKVVRDRLKTYDDVSAHPELGW-YGQQGN--REWKQYFLHD-DNN-Y-TTGQCPFKLK---RS---E------Q-
HborDRAFT_2010_Hbor_227881355                           V-QSLVD--QETMAARV-EAGEMP--D-W------------------------------------------------------------AVRHFETFTDAL-LGERNGT-PFPCFFGAQSVRDGEPVYTAVP--SMTDKDALL--DFRDALLEYLDVYR-D-LP-G---RT------SFVTFFKPPERE-FS-----EGDYHEILWHLLQFLHVHDP---EPWPED-IPTDPDDPHWEFCFGGEPMFPTCRAPFY--DERKSRY---CPVGLEITFQPRTLFD--GITHDTEAG-QKARETIQSRMEEYDGVCPHADLGD-WGVESD--REWPQYLFRE-DPD-D-SPDECPIV-----IT---R------E-
Bsph_2060_Lsph_169827609                                D-VGIFD--RKWIEENLDT---LP--I-W------------------------------------------------------------QQSAYMDFANTI-ADEEN---TFPCVPARMGFLSNHLRYSFIG--DPRKEQSIH--ELAHCLKEFTKSSQ-T-FG-K---YT------TLTVFFHTPQDM-LCNYK--VEDYQQLFWTILNQLTNIDD---LDWPEQ-IPTDPAHNEWEFCFNGEPYFISCATPAH--KMRRSRH---F-STLLMAIQPRWIFE--EINDSTAFG-RKLKKLIRQRIAHYDALPGHPDLKW-YGQEDS--YEWKQYFLSD-DHQ---SPSKCPF--------------------
BMD_2530_Bmeg_294802309                                 L-KMLFR--ANEI-LESTH---VP--F-W------------------------------------------------------------GKDAFQYFRSDI-LSEEN---PFPCILGVEGFKKDLLRFSFVT--TPYNYQDIR--NVALALREYIDTFK-T-IG-R---YT------SFVLFFKPEHKE-RSMEE-----YETMFWDTLTFLHQIDQ---KKWPQD-IPKDPKDPLWEFCFHGEPIFVVCNTPAH--SLRKSRK---S-RGFMITFQPRWVFE--GMTGDSKIG-RHVQKLVRDRLKTYDDVSAHPELGW-YGQQEN--REWKQYFLHD-HNN-Y-TAGQCPFKLK---RS---E------Q-
HQ2279A_Hwal_110668212                                  V-GNLQD--QATVEERV-ESGAAP--D-W------------------------------------------------------------VTAHWNTFRDGL-LGERNDA-PFPCFFGAESVANGDPLYTAVS--SMTEKNALL--NLGRTLLSYLEIYQ-D-YS-D---RA------SLVVFFRPPNQE-LS-----EGEYHEALWHILQFLHVHDT---KPWPKD-IPTDPDDPHWEFSFGGEPMFPTCRAPFY--DTRKSRY---CPIGLEITFQPRALFENLRVTADTAMG-ERARETIQDRLEDYDGVCPHADLGD-WGVEED--REWPQYMFSS-DDK-Q-APDECPIN-----IT---R------E-
BT9727_2874_Bthu_49478000                               M-TILYD--KDSLQKNIIT---LP--N-W------------------------------------------------------------QQEAFRSFKLKM-TDKDK---LFPCIPAQHGFTANHLRYGFIG--NPRDTNTSE--DFAALLKEYTECSR-D-TG-Q---YA------SLIVFIHTPIDL-LPETT--VEDFEHMYWSLLNTTSRLDE---MEWPTH-IPNDPMENTWEFCFHNESYFVYCATPAH--VNRQSRH---F-SCMMLALTPRWVLQ--GIMNSEKRS-RKLKNLIRQRLAAYDKAPIHPSLKD-YGGKDN--YEWQQYFLRD-DET---IPSKCPFS-----RI---I------K-
BCAH820_3125_Bcer_218904241                             M-TILYD--KDSLQKNIIT---LP--N-W------------------------------------------------------------QQEAFRSFKLKM-TDKDK---LFPCIPAQHGFTANHLRYGFIG--DPRDTNTSE--DFAALLKEYTECSR-D-TG-Q---YA------SLIVFIHTPIDL-LPETT--VEDFEHMYWSLLNTTSRLDE---MEWPTH-IPNDPMENTWEFCFHNESYFVYCATPAH--VNRQSRH---F-SCMMLALTPRWVLQ--GIMNSEKRS-RKLKNLIRQRLAAYDKAPIHPSLKD-YGGKDN--YEWQQYFLRD-DET---IPSKCPFS-----RI---I------K-
BCW_3027_Bcer_196032578                                 M-TILYD--KDSLQKNIIT---LP--N-W------------------------------------------------------------QQEAFRSFKLKM-TDKDK---LFPCIPAQHGFTANHLRYGFIG--DPRDTNTSE--DFAALLKEYTECSR-D-TG-Q---YA------SLIVFIHTPIDL-LRETT--VEDFEHMYWSLLNTTSRLDE---MEWPTH-IPNDPMENTWEFCFHNESYFVYCATPAH--VNRQSRH---F-SCMMLALTPRWVLQ--GIMNSEKRS-RKLKNLIRQRLAAYDKAPIHPSLKD-YGGKDN--YEWQQYFLRD-DET---IPSKCPFS-----RI---I------K-
bthur0012_29100_Bthu_228915692                          M-TILYD--KDSLQKNIIT---LP--H-W------------------------------------------------------------QQEAFRSFKLKM-TDKDK---LFPCIPAQHGFTANHLRYGFIG--NPRDTNTSE--GFAALLKEYTECSR-D-TG-Q---YA------SLIVFIHTPIDL-LRETT--VEDFEHMYWSLLNTTSCLDE---MEWPTH-IPNDPMENTWEFCFHNESYFVYCATPAH--VNRQSRH---F-SCMMLALTPRWVLQ--GIMNSEKRS-RKLKNLIRQRLAAYDKAPIHPSLKD-YGGKDN--YEWQQYFLRD-DET---IPSKCPFS-----RI---I------K-
bcere0021_29050_Bcer_229092113                          M-TILYD--KDSLQKNIIT---LP--H-W------------------------------------------------------------QQEAFRSFKLKM-TDKDK---LFPCIPAQHGFTANHLRYGFIG--DPKDTNTSE--DFAALLKEYTECSR-D-TG-Q---YA------SLIVFIHTPIDL-LQETT--VEDFEHMYWSLLNTTSRLDE---MEWPTH-IPNEPMENTWEFCFHNESYFVYCATPAH--VNRQSRH---F-SCMMLALTPRWVLQ--GIMNSEKRS-RKLKNLIRQRLAAYDKAPIHPSLKD-YGGKDN--YEWQQYFLRD-DET---IPSKCPFS-----RI---I------K-
bthur0007_14660_Bthu_228945303                          K-SYLLD--NEGMRMRT-D---IP--N-W------------------------------------------------------------VAKEFKNFSNVV-LEP-----TFPCYFGLTALKKNELRYSFLS------HNDWS--HLPHTMLSFLELMK-E----RPIVRR------GFFLFVEPECEE----QS--IEYYRDYFWKVLQYLHENDD---QTWPKQ-IPEDPDHNLWEFSFGGEPIFAFGNAPAY--KQRKTRH---LGNSLVIGFQPRTIFD--GLEGDRPKG-AYSRQTVRDRVEKWDQLPKHPNISH-YGDPEH--REWKQYFIGD-DVE-P-IKGKCPFL---H-KI---E------K-
bthur0004_28750_Bthu_228966057                          M-TMLYN--KDSLQKNIIT---LP--H-W------------------------------------------------------------QREAFRSFKLKM-TDKDK---PFPCIPAQHGFTANHLRYGFIG--DPRDTNTSE--DFAALLKEYTECSR-D-TG-Q---YA------SLIVFIHTPTDL-KQETT--VEDFEHIYWSLLNTTSRLDE---MEWPMH-IPNDPMENTWEFCFHNESYFVYCATPAH--VNRQSRH---F-SCMMLALTPRWVLQ--GIMNSEKRS-RKLKNLIRQRLAAYDKAPIHPSLKD-YGEKDN--YEWQQYFLRD-DET---IPSKCPFS-----RI---I------K-
Hmuk_2127_Hmuk_257388174                                V-GELQD--QATVEERV-ASGATT--D-W------------------------------------------------------------VVDHWTTFRDGL-LGERNGT-PFPCFFGAESVADGDPLYTAVP--SMTDKDALL--DLGRTLLQYLDTYQ-E-HS-D---RA------SLVAFFKPPERE-LT-----EAQYHEALWNILQFLHVHDP---APWPED-IPTEPDDPHWEFSFGGEPIFPTCRAPFY--EGRKSRY---CPVGLEITFQPRALFEDMGVTADTEAG-EHARGIIQDRLEGYDGVCPHADLGD-WGVEGD--REWPQYMLSE-DDR-Q-APSECPIS-----TT---R------E-
BALH_2785_Bthu_118478419                                M-TILYD--KDSLQKNIIP---LP--N-W------------------------------------------------------------QQEAFRSFKLKM-TDKDK---LFPCIPAQHGFTANHLRYGFIG--DPRDTNTSE--DFAALLKEYTECSR-D-TG-Q---YA------SLIVFIHTPIDL-LPETT--VEDFEHMYWSLLNTTSRLDE---MEWPTH-IPNDPMENTWEFCFHNESYFVYCATPAH--VNRQSRH---F-SCMMLALTPRWVLQ--GIMNSEKRS-RKLKNLIRQRLAAYDKAPIHPSLKD-YGGKDN--YEWQQYFLRD-DET---IPSKCPFS-----RI---I------K-
BC059799_3085_Bcer_196037883                            M-TILYD--KDSLQKNIIT---LP--H-W------------------------------------------------------------QREAFRSFKLKM-TDKDK---LFPCIPAQHGFTANHLRYGFID--DPRDTNTSK--DFAALLKEYTECSR-D-TG-Q---YA------SLIVFIHTPIDL-LRETT--VEDFEHMYWSLLNTTSRLDE---MEWPTH-IPNDPMENTWEFCFHNESYFVYCATPAH--ANRQSRH---F-SCMMLALTPRWVLQ--GIMNSEKRS-RKLKNLIRQRLAAYDKAPIHPSLKD-YGEKDN--YEWQQYFLRD-DET---IPSKCPFS-----RI---I------K-
Bsubs1_010100014121_Bsub_221310509                      M-TELYA--KSDLEKIKET---LP--G-W------------------------------------------------------------QLDSFNYLNEKI-GDKEN---KFPCIPGRQAFLSDQLRIAFVG--DPRNPETAK--ELAPLLTRYGTISR-E-TG-K---YA------SLTVIFHTPEEL-LTDYK--IEDYESLFWQLLNSLSMEDP---ADWPDD-IPENPDNFQWEYCFNGEPYFVLCATPAH--SKRKSRS---F-PYFMLTFQPRWVFE--DLNDTTAFG-RNMSKQIRKRLEAYDEVPIHPHLGW-YGKKDN--LEWKQYFLRD-DEN---QVSQCPFM-----KM---K------N-
ORF4_Bsub_1217881                                       M-TELYA--KSDLEKIKET---LP--G-W------------------------------------------------------------QLDSFNYLNEKI-GDKEN---KFPCIPGRQAFLSDQLRIAFVG--DPRNPETAK--ELAPLLTRYGTISR-E-TG-K---YA------SLTVIFHTPEEL-LTDYK--IEDYESLFWQLLNSLSMEDP---ADWPDD-IPENPDNFQWEYCFNGEPYFVLCATPAH--SKRKSRS---F-PYFMLTFQPRWVFE--DLNDTTAFG-RNMSKQIRKRLEAYDEVPIHPHLGW-YGKKDN--LEWKQYFLRD-DEN---QVSQCPFM-----KM---K------N-
bcere0017_56700_Bcer_229119440                          K-SYLYN--QNRILDEK-NNDFLE--S-W------------------------------------------------------------KIHAYNSFHKSI-MD--NSK-PFPCYFAVDSEKHGWSRYLFID--SAYDQKELL--KLRDGIYEYIKTYQ-Q-IA-K---RT------TLVIFFKPSSKQ-LL-----AEEYKKQFWNVLQFLIENDP---EPWNPE-IPTDPYHAKWEFCFAGEPIFVVCRAPIY--AERQSRY---TENGLEITLQPRGTLD--DITGNTKQG-KQVRKVIRKRLLAYDNISMHPDIGD-YGAENS--HEWKQYILPE-TNE-E-SVMRCPIT-----GV---K------K-
bcere0021_28190_Bcer_229092024                          K-MYLYD--QKRIIEEQ-NKGILD--E-W------------------------------------------------------------KTISFNNFHDSI-LD--QSK-PFPCYFAVDSEKHGWSRYIFSE--SAYDENELF--KLRDGIYEYIKTYQ-Q-IA-K---RT------TLVIFFKPSNKQ-LL-----AEEYKKQFWNVLQFLMRNDP---EPWNAE-IPIDPYNAKWEFCFGGEPIFVVCRAPIY--SERKSRY---TGTGLEITLQPRGTLD--DITGDTKQG-KQVRKVIRKRLLAYDDIPMHPDIGD-YGIKET--FEWKQYMLPE-TNE-E-SVMRCPIT-----GL---K------K-
BB14905_01070_Bsp._126651210                            D-VGVFD--RKWIEEHLDT---LP--I-W------------------------------------------------------------QQSAYTDFAATI-ADEDN---TFPCVPARMGFLSNHLRYSFIG--DPRKEPSIH--ELAQCLKEFAKSSQ-T-FG-K---YT------TLTVFFHSPQDM-LSHYK--VEDYQQLFWTILNQLTKIDD---LDWPEE-IPTDPNHNEWEFCFNGEPYFISCATPAH--KLRRSRH---F-STLLMAIQPRWIFE--EINDSTAFG-RKLKKLIRQRIAHYDALPGHPDLKW-YGQENS--YEWKQYFLSD-DNQ---SPSKCPF--------------------
BA_3123_Bant_30263070                                   M-TILYD--KDSLQKNIIT---LP--H-W------------------------------------------------------------QQEAFGSFQLKM-TDKNK---PFPCIPAQHGFTANHLRYGFID--DPRDTNTSE--DFAALLKEYTECSR-D-TG-Q---YA------SLIVFIHTPIDL-LRETT--VEDFEHMYWSLLNTTSRLDE---MEWPTH-IPNDPMENTWEFCFHNESYFVYCATPAH--VNRQSRH---F-SCMMLALTPRWVLQ--EIMNSEKRS-RKLKDLIRQRLAAYDKAPIHPSLKD-YGEKDN--YEWQQYFLRD-DET---IPSKCPFS-----RI---I------K-
bcere0001_27520_Bcer_229197201                          K-MYLYD--QKRIIEEQ-NKGILD--E-W------------------------------------------------------------KTISFNNFHDSI-LD--QSK-PFPCYFAVDSEKHGWSRYIFSE--SAYDENELF--KLRDGIYEYIKTYQ-Q-IA-K---RT------TLVIFFKPSNKQ-LL-----AEKYKKQFWNVLQFLMRNDP---EPWNAE-IPIDPYNAKWEFCFGGEPIFVVCRAPIY--SERKSRY---TGTGLEITLQPRGTLD--DITGDTKQG-KQVRKVIRKRLLAYDDIPMHPDIGD-YGIKET--FEWKQYMLPE-TNE-E-SVMRCPIT-----GL---K------K-
bcere0002_58280_Bcer_229194240                          M-AILYD--KDSLQKNIIN---LP--H-W------------------------------------------------------------QQEAFRSFKLKM-TDKHK---PFPCIPAQHGFTANHLRFGFIG--DPRNMNTSA--DFAVLLKEYTECSR-E-TG-Q---YA------SLIVFIHTPIDL-ERETT--VEDFEHIYWSLLNTTSRLDE---MEWPTH-IPNNPMENTWEFCFHNESYFVYCATPAH--VNRRSRH---F-SCMMLALTPRWVLQ--GIMSSEKRS-KKLKNLIRQRLAAYDKAPIHPSLKD-YGEKDN--YEWQQYFLRD-DET---IPSKCPFS-----RI---I------K-
yqcI_Bpum_157691082                                     M-AQLYA--KSCLDQNLRS---LE--E-W------------------------------------------------------------KQDAFTLFGEMV-GDEAD---TFPCVPGRQGFFLDHLRYGFVG--DPRKEEAVE--ELAQLLREYQSCAK-E-TG-Q---YA------SFICFFETPQDL--KESS--IEEFEQQFWSLLQRLHQKDE---VPWPED-IPLDTHHHEWEYCFHGEAYFILCSTPAH--KLRKSRH---F-PYVLMAFQPRWVFE--KLNGSTKFG-QKMSQLVRKRLKAYDEVGVHPSLKW-YGDPTN--HEWKQYFLPD-EEAEKPASAKCPFT-----AL---K------N-
Htur_3074_Htur_284166333                                I-QSLMD--QETLDRRV-RAGETP--E-W------------------------------------------------------------VTDHWETFQNGL-LGERNGS-PFPCFFGAESVQQGEPLYTAVP--SMNDADALL--TLRDRILEYLEIYR-D-HS-E---RA------SLVTFFEPPAEP-LS-----EREYHDALWHVLQTLHCHDP---EPWPED-IPTDPNDPYWEFCLGGEPMFPTCRAPFY--DDRKSRY---CPIGLEITFQPRSLFEDLGVTADTEAG-QHAREIIQGRLEAYDGVCPHADLGD-WGVDGD--REWPQYMLSA-DED-Q-APAECPIT-----IT---R------E-
bmyco0003_54230_Bmyc_229000944                          K-SYLYD--QKRILKEQ-SIDFLE--D-W------------------------------------------------------------KIQSFNNFHTSI-MD--NSK-PFPCYFAVDSEKHGWSRYLFAD--SAYDERELM--KLRDGIYEYIKTYQ-Q-IA-K---RT------TLVIFFKPSDKQ-LM-----AEEYKKQFWNVLQFLIKHDP---EPWNSE-IPTDPYHAKWEFCFAGEPIFVVCRAPIY--AERKSRY---TDTGLEITLQPRGTLD--DITGDTKQG-KQVRKVIRKRLLAYDDISIHPDIGD-YGTKDS--YEWRQYMLPE-TND-E-SVVRCPIT-----GA---K------K-
HVO_2327_Hvol_292656455                                 H-QVLME--QDVLRHRL-DDGDLP--D-W------------------------------------------------------------ARKHYETFRETM-LGDRDGA-PFPCYFGIESERNGDALYTFVD--SMTDKDALL--ALRDTLLEYLDVYP-D-YS-E---AC------SLVTFFKPPAAD-FT-----EADYHERLWHILQFLHVNDP---EPWPAD-IPTDPDDPTWEFSFGGEPMFPTTRAPFY--DERISRY---CPWGLEITFQPRALFD--GITADTEAG-QQARAVIQNRIEEYDGVCPHADLGD-WGVEGD--REWPQYMFSA-DES-Q-APDECPIR-----IT---R------E-
yqcI_Bcer_52142411                                      M-TILYD--KDSLQKHIIT---LP--H-W------------------------------------------------------------QQEAFCSFKLKM-TDKNK---PFPCIPAQHGFTANHLRYGFIG--DPRDPNTSE--DFATLLKEYTECSR-D-TG-Q---YA------SLIVFIHTPIDL-MRETT--VKDFEHMYWSLLNTTSRLDE---MEWPTH-IPNDPMENTWEFCFHNESYFVYCATPAH--VNRQSRH---F-SCMMLALTPRWVLQ--GIMNSEKRS-RKLKNLIRQRLAAYDKAPIHPSLKN-YGEKDN--YEWQQYFLRD-DET---IPSKCPFS-----RI---I------K-
yqcI_Bamy_154684802                                     A-SELYT--KSCLEEKLLD---LP--N-W------------------------------------------------------------QRVAFELLSETI-GDKAD---TFPCIPGRQGFLTDQLRISFAG--DPREEGTPE--EVGMLLSEYGKISR-N-TG-R---YA------SLLVIFDTPEDL-AEHYS--VEAYEELFWSFLNRLSGCDP---KDWPED-MPEDPEHYKWEFCFDGEPYFILCATPAH--EARQSRS---F-PFFMLAFQPRWVFE--GLNDSTAFG-RNMSRLIRKRLEAYDEAPLHPRLGW-YGGKDN--LEWKQYFLRD-DET---EVSKCPFS-----YL---K------R-
BCH308197_3074_Bcer_206974502                           M-TILYD--KDSLQKHIIN---LP--H-W------------------------------------------------------------QQEAFCSFKLKM-TDKNK---PFPCIPAQHGFTANHLRYGFID--DPRDTNTSE--DFATLLKEYTECSR-D-TG-Q---YA------SLIVFIHTPIDL-MRETT--VKDFEHMYWSLLNTTSRLDE---MEWPTH-IPNDPMENTWEFCFHNESYFVYCATPAH--VNRQSRH---F-SCMMLALTPRWVLQ--GIMNSEKRS-RKLKNLIRQRLAAYDKAPIHPSLKD-YGEKDN--YEWQQYFLRD-DET---IPSKCPFS-----RI---I------K-
bcere0010_28710_Bcer_229156686                          M-TILYD--KDSLQKHIIN---LP--H-W------------------------------------------------------------QQEAFCSFKLKM-TDKNK---PFPCIPAQHGFTANHLRYGFIG--DPRDTNTSE--DFATLLKEYTECSR-Y-TG-Q---YA------SLIVFIHTPIDL-MRETT--VKDFEHMYWSLLNKTSRLDE---MEWPTH-IPNDPMENTWEFCFHNESYFVYCATPAH--VNRQSRH---F-SCMMLALTPRWVLQ--GIMNSEKRS-RKLKNLIRQRLAAYDKAPIHPSLKD-YGEKDN--YEWQQYFLRD-DET---IPSKCPFS-----RI---I------K-
ycgG_Bsub_255767087                                     V-NGIYT--KSFLERIQEE---LP--E-W------------------------------------------------------------QRIAFELLAETL-GDDAD---TFPCIPGRQAFLTDQLRIAFAG--DPRENRTAE--ELAPLLAEYGKISR-D-TG-K---YA------SLVVLFDTPEDL-AEHYS--IEAYEELFWRFLNRLSHQDE---KEWPED-IPADPEHYKWEFCFDGEPYFILCATPGH--EARRSRS---F-PFFMVTFQPRWVFD--DLNGSTAFG-RNMSRLIRSRLEAYDQAPIHPQLGW-YGGKDN--REWKQYFLRD-DEK---QVSKCPFS-----YL---K------N-
BA_1630_Bant_30261700                                   K-SYLLD--NEGMRTRT-D---IP--N-W------------------------------------------------------------VAKEFENFSNVI-LEP-----TFPCYFGVTALKKNELRYSFLS------HNDWS--HLPNTMLSFLELMK-E----RPVVRR------GFFLFVEPECEE----QS--IEYYRDYFWKVLQYLHENDD---QTWPKQ-IPEDPDHYLWEFSFGGEPIFAFGNAPAY--KQRKTRH---LGNSLVIGFQPRTIFG--GLEGDRPKG-AYSRQTVRDRVEKWDQLPKHPNISH-YGDPEH--REWKQYFIGD-DVE-P-IKGKCPFL---H-KI---E------K-
ycgG_Bsub_291482697                                     I-NGEWDLHQKFSGTYSGE---LP--E-W------------------------------------------------------------QRIAFELLAETL-GDDAD---TFPCIPGRQAFLTDQLRIAFAG--DPRENRTAE--ELAPLLAEYGKISR-D-TG-K---YA------SLVVLFDTPEDL-AEHYS--IEAYEELFWRFLNRLSHQDE---KEWPED-IPADPEHYKWEFCFDGEPYFLLCATPAH--EARKSRS---F-PFFMITFQPRWVFE--DLNGSTAFG-RNMSRLIRSRLEAYDQAPIHPQLGW-YGGKDN--REWKQYFLRD-DEK---QVSKCPFS-----YL---K------N-
BAT_3821_Bpum_194017444                                 M-AQLYA--KSCLDQNLRS---LE--E-W------------------------------------------------------------KQDAFTLFGEMV-GDEAD---TFPCVPGRQGFFLDHLRYGFVG--DPRKEEAVE--ELAQLLREYQSCAK-E-TG-Q---YA------SFICFFETPQDL--KESS--LEELEQQFWSLLQRLHQKDE---VPWPED-IPLDAHHHEWEYCFHGEAYFILCSTPAH--KLRKSRH---F-PYVLMAFQPRWVFE--KLNGSTKFG-QKMSQLVRKRLKAYDEVGVHPSLKW-YGDPTN--HEWKQYFLPD-EEAEKPASAKCPFT-----AL---R------N-
BSG1_03505_Bsp._149180050                               --MNLLT--KSWIDANQTE---LE--E-W------------------------------------------------------------KRNAYSEFSSMM-LSTEN---PYPCVPGIQGFQKDQLRFGFTG--NPHDDASRK--EFASMLKEYGKISR-E-TG-N---YA------SIVVFFDSREVT-YEKDA--NNQYQDLFWSILNRVHELDE---APWPED-IPEDPHDTAWEFCFDGEPYFAFCATPSH--FERKSRH---F-PYFLLALQPRWVFD--EINASTTFG-QKLKKVIRKRLKDFDGADAHPNLKW-YGQDDN--HEWKQYFLSD-DGQ---TPSKCPFT-----AM---K------N-
OB3195_Oihe_23100650                                    M-SILFN--NVTIKQNWEL---LS--P-W------------------------------------------------------------KQDAYQYFQSMI-GEKND----YPCVPARQGLKNNMLRFGFLD--NVDDTKV-----LASSLKEYGDTSK-A-IG-Q---YT------SLIIFI--PMDD-DTQAT--VEDYQVLFWDLLSDVTNYDT---SDWPAT-IPDNPEHHEWEFCFDGEPYFAFCATPVH--QLRKSRY---F-PYMMLAFQPRFVFD--ELNASTSYG-RKMKKVIRQRLQAFDSIPAHPDLKW-YGNSDN--HEWKQYFIGD-DDQ---TLSKCPFT-----RF---K------Q-
BCAH820_1698_Bcer_218902815                             K-SYLLD--NEGMRTRM-D---IP--N-W------------------------------------------------------------VAKEFENFSNVV-LEP-----TFPCYFGLNALKKNELRYSFLS------HNDWS--HLPHTMLSFLELMK-E----RPIVRR------GFFLFVEPECEE----QS--IEYYRDYFWKVLQYLHENDD---QTWPKQ-IPEDPDHYLWEFSFGGEPIFAFGNAPAY--KQRKTRH---LGNSLVIGFQPRTIFD--GLEGDRPKG-AYSRQTVRDRVEKWDQLPKHPNISH-YGDPEH--REWKQYFIGD-DVE-P-IKGKCPFL---H-KI---E------K-
Bcer98_1717_Bcyt_152975498                              --MILYD--KEMLQENRAN---VP--K-W------------------------------------------------------------QQDVFHSFVLKM-TNTHQ---PFPCIPAQKGFLFNHLRYGFIG--DPRKEATSK--ELAHLLKAYVKQSK-D-LG-N---YT------SLIVFIETPTKI-LTKAT--VTDYENWYWSLLNQTSKLDE---KEWPEH-VPSDPMKNTWEFCFHEEPYFVYCATPAH--VKRQSRY---F-PCMMLAITPRWVLQ--EIMKSKKLS-EKLKQAIRKRLHSYDTAPIHPSLKW-YGEKEN--YEWQQYFLRD-DET---IPSKCPFS-----RA---M------K-
ABI75127_Acir_114462391                                 F-TPIWN--KSQMESVLVEEC-LP--A-W------------------------------------------------------------VRTNYSTFVETI-RDP-----AFPCFWGTIGEQKGMIRYLIAS--SLTEPSVVE--HTLEGVYNYINEVN-E----NELLQHENADLLTLVIFFPPEPTV-LT-----VEEYSRQAFAFLNALHSLDA---VSCPCH-WSADPQSPNWSYSLGGCALFVSVSTPAN--QKRRSRH---LGSGMTFVITPVEVLL--RKHGGENSS-------IFRRVREYDGISPHPNLLI-MPGSSKVGNELTVQVLPD-DND---SEISFDFQ---Y-KF---K------D-
sxtQ_Lwol_195984499                                     V-TPIWN--KSQMESFLVEEC-LP--A-W------------------------------------------------------------VRTSYSTFVETI-RDP-----AFPCFWGTIGEQKGMIRYVIAS--SLTEPSVVE--HTLEGVYNYIDEVN-E----NELLQHENADLLTLVIFFPPEPTV-LT-----VEEYARQAFNFLNALHSLDK---VPWPTD-WSADPQSPDWSYSLGGCALFVNVSTPAN--QKRRSRH---LGSGMTFVIAPVEVLL--RKHGGENSS-------IFRRVREYDGIPPHPNLLI-MPGSGKVGNELTVQVLPD-NND---SEISFDFQ---Y-KF---K------D-
Hlac_2102_Hlac_222480512                                TEGVLLD--QKTLHDRL-D--DAP--G-W------------------------------------------------------------LQDHYRTFRESM-LGERDDS-PFPCYFGIEVEREGDLLYAACE--STTDPAALL--RLRDVLVEYLDAYA-D-HA-D---RA------PLAVFFRPPEGD-PG-----ESHYHERLWHVLEFLHVHDP---EPWPDD-IPTDPDTPRFEFCFGGEPLFPTSRAPFY--DERKSRY---SPVGLEVTFQPRAVFD--GLTADTEAG-QHARETIRERMGEYDGVCPHADLGD-WGVEGD--REWTQYLFRE-DDD-A-SPDICPLS-----PT---R------N-
BC1003DRAFT_5936_Bsp._289637439                         DWKSRII--AGRATGAAVS---AP--A-W------------------------------------------------------------LDASYATLREQV-LDPA-----YPCFFGTMAERRGEMFYSFV------NGRDLR--DLPATMQTFAELAV-Q-PQYR---RN------NIAVFFEPDPEP-LSHDA-----YRTLFWGILQQLHDIDP---DPAADQ-QP-DPMDEAWEFSYAGVQMFVVCACPSF--RARHSRN---LGPGMVLLFQPRSVFV--DTITNKVIG-REARNQVRKRLETWDDIPAHPDLGF-YGDPGN--LEWKQYFLDD-ANA-P-IEERCPFL---K-RR---A------Q-
CRD_02142_Rbro_282897220                                V-TPIWN--KSQMESSLLEEC-LP--A-W------------------------------------------------------------VRTSYSTFVETI-SDS-----AFPCFWGTIGEQKGMIRYLIVS--SLTDPILVE--HTLEGIYKYIDEVN-E----NELLQHENADLLTLVIFFPPEPTV-LT-----VEEYAGQAFDFLNALHSLDA---VSCPCH-WSADPQSANWSYSLGGCALFVSVSTPAN--QKRRSRH---LGSGMTFVITPVEVLL--NKHGGENSS-------IFRRVREYDGIPPHPNLLI-MPGNGKVGNELTVQVLPD-NND---SEISFDFQ---Y-KF---K------D-
G11MC16DRAFT_3288_Gsp._196250843                        ------------------------------------------------------------------------------------------------MGKVV-SDK-----NFPCHFGTIAEKKGDLRYFYIE------NHDLS--PLPNVLREFLKLSR-E----NEHNKH------ALVVFVQPEIPE----QS--FDYYENYFWNILKYLHENDE---KEWNAN-IPTDPDDPLWEFCFDGEPIFVSANMPAY--KHRITRN---MGKSLILIFQPRRIFA--DIT---PKA-I---DLIRSKVESIENLPIHPDLGR-YGDESN--REWKQYIITD-DNN-P-RKGVCPFH---P-KS------------
BMQ_pBM60088_Bmeg_294505791                             M-RLFKD--IEKVNQ--ID---FL--P-W------------------------------------------------------------QNDALKAFSAKM-KDREY---LFPCIPAIQSFSLGHLRYGFIG--HPESNQTSI--ELASLLKEFTINCN-E-YG-K---YT------TLIIFFETPQNL-ITNRT--VEDFELLFWKQLSNLNKLDE---KGWPIH-IPRNPSEHEWEYCFHGEQYFMYCATPKH--EKRKSRY---F-PYMMMAITPRWVLQ--EFNKNKRYA-KKIKEQVRERIKKYDNISTHSALNS-YGNIDN--HEWKQYFLRD-DNT---ELPKCPFL-----RS---L------V-
Cwoe_2451_Cwoe_284043909                                ---------MRSVTRAA-----ID--A-F-------------TALA-------------------------------------------QAPSVAAFAPIR-------R-ATHCAYAGGSVIWGARPFDT----DASLAANLD--GFAADLTVFVDAAG-E--------LR--LD--AFVIELPAAHGT-----T--LDRLAQTTHALLRGLAARDP---RAEGA--LDGEVDEPAWCYAFGGDPLFVNTFAPCY--PSEHSRYGFGV-AATFLLLQPRHSFA----------------RVVRRGETVLPVAARHRIRSD-YAAHAR-------------GYD-H-AISAAPFE-A-R-RI---V------R-
sxtQ_Asp._195984460                                     V-TPIWN--KSQMESLLVEEY-LP--A-W------------------------------------------------------------VRTNYSTFVETM-RDP-----AFPCFWGTIGEQKGMIRYLIAS--SLTEPSVVE--HTLEGVYNYINEVN-E----NELLQHENADLLTLVIFFPPEPTV-LT-----VEEYSRQAFKFLNALHSLDA---VSCPCH-WSADPQSPNWSYSLGGCALFVSVSTPAN--QKRRSRH---LGSGMTFVITPVEVLL--RKHGGENSS-------IFRRVREYDGIPPHPNLLI-MPGSSKVGNELTVQVLPD-DND---SEISFDFQ---Y-KF---K------D-
sxtQ_Crac_114462371                                     V-TPIWN--KSQMESSLLEEC-LP--A-W------------------------------------------------------------VRTSYSTFVETI-SDS-----AFPCFWGTIGEQKGMIRYLIVS--SLTDPILVE--HTLEGIYKYIDEVN-E----NELLQHENADLLTLVIFFPPEPTV-LT-----VEEYAGQAFDFLNALHSLDA---VSCPCH-WSADPQSANWSYSLGGCALFVSVSTPAN--QKRRSRH---LGSGMTFVITPVEVLL--NKHGGENSS-------IFRRVRQYDGIPPHPNLLI-MPGNGKVGNELTVQVLPD-NND---SEISFDFQ---Y-KF---K------D-
bcere0009_14380_Bcer_229160647                          E-SYLLD--NEGMKTRT-D---IP--N-W------------------------------------------------------------VAKEFQNFSSVV-LEP-----TFPCYFGLTALKKNELRYSFLS------HNDWD--HLPHTMLSFLELMK-E----RPIVRR------GFFLFVEPECEE----QS--LEYYREYFWKVLQYLHKNDN---QTWPRQ-IPQDPDHYLWEFSFGGEPIFAFGNAPAY--KQRKTRH---LGNSLVIGFQPRTIFD--GLEGDRPKG-AYSRQTVRERVEKWDQLPKHPNISH-YGDPDH--REWKQYFIGD-DIE-P-IKGKCPFL---H-KI---E------K-
VNG1099C_Hasp_15790191                                  L-QAVVD--QQTLADRV-AAGDYP--D-W------------------------------------------------------------VRSHWETFSESV-TGDRDGT-PFPCHFGAESVANGEPLYTVVP--SLTDPDALL--GFRDALLEYLTEHG-D-RD-A---RV------SFVTFWKPPEDE-FS-----EADYHEALWHILSVLHVHDP---EPWPAD-VPTDTDSPGWEFCFGGEPLFPTCRAPFYAAHDRYSRY---CPVGLEITFQPAGLFE--GITADTDAG-QRARAAIQNRIEEYDGQCPHADLGD-LGVDGD--REWVQYLFRE-DDA-Q-APTECPAT-----FT---R------E-
bthur0010_14510_Bthu_228926735                          K-SYLLD--NEGMRMRT-D---IP--N-W------------------------------------------------------------VAKEFKNFSNVV-LEP-----TFPCYFGLTALKKNELRYSFLS------HNDWS--HLPHTMLSFLELMK-E----RPIVRR------GFFLFVEPECEE----QS--IEYYRDYFWKVLQYLHENDD---QTWPKQ-IPEDPDHYLWEFSFGGEPIFAFGNAPAY--KQRKTRH---LGNSLVIGFQPRTIFD--GLEGDRPKG-AYSRQTVRNRVEKWDQLPKHPNISH-YGDPEH--REWKQYFIGD-DVE-P-IKGKCPFL---H-KI---E------K-
bthur0005_28310_Bthu_228959344                          --------------------------------------------------------------------------------------------------------------MFPCIPAQHGFTANHLRYGFIG--DPRDTNTSE--GFAALLKEYTECSR-D-TG-Q---YA------SLIVFIHTPIDL-LRETT--VEDFEHIYWSLLNKTSCLDE---MEWPTH-IPNDPMENTWEFCFHNESYFVYCATPAH--VNRQSRH---F-SCMMLALTPRWVLQ--GVMNSEKRS-RKLKNLIRQRLAAYDKAPIHPSLKD-YGEKDN--YEWQQYFLRD-DET---VPSKCPFS-----RI---I------K-
GYMC10_5619_Gsp._261409392                              M-SGLMR--LDDMDAAYGL---PE--E-W------------------------------------------------------------QREANVKLTEKM-TDRSA---KFPCIPAVQGHALNHFRYGFIN--RGESQEAAG--QLAALLHEFGLGSR-G-FG-P---YT------SLVVFINTEHGR-DSLEG--VEYYERLFWQLLSRTTEYDT---EPWPAD-LPGEPDDHLWEFCYAGEPYFVYCGTPAH--ELRQSRY---F-PYMMLAFTPRWVLK--QFNARARQA-EHTKVLIRKRLTEYDPVPPHPDLKF-YGAEDN--YEWKQYFLRD-NEE---SLSKCPFA-----RL---H------K-
Nmag_3207_Nmag_289582853                                T-QSLMD--QETLDRRV-QAGETP--Q-W------------------------------------------------------------VADHWESFREGL-LGERNNS-PFPCFFGAESVAAGEPIYTAVP--SMSDADALL--TLRDRILEYLDVYQ-D-HA-G---RA------SLVTFFKPPEEP-LS-----EREYHDALWHILQTLHLHDP---EPWPED-IPTDPDDKHWEFCLGGEPMFPTCRAPFY--DTRKSRY---CPIGLEITFQPRALFEDLNVTAEYEAG-QRARDVIQDRLEEYDGVCPHADLGD-WGVDGD--REWPQYMLSS-DEE-Q-APAECPIT-----VT---R------E-
bthur0011_27640_Bthu_228921767                          M-TMLYD--KDSLQKNITN---LP--H-W------------------------------------------------------------QQEAFRSFKLKM-TDKDK---PFPCIPAQHGFTANHLRYGFIG--DPRDTNTSA--DFATLLKEYTECSR-D-TG-Q---YA------SLIVFIHTPIDL-LRETT--VEDFEHIYWSLLNTTSRLDE---MEWPMH-IPNDPMENTWEFCFHNESYFVYCATPAH--VNRQSRH---F-SCMMLALTPRWVLQ--GIMNSEKRS-RKLKNLIRQRLAAYDKAPIHPSLKD-YGEKDN--YEWQQYFLRD-DET---IPSKCPFS-----RI---I------K-
rrnAC2099_Hmar_55378806                                 V-GKLQD--QETLAARV-ERGDAP--E-W------------------------------------------------------------VGAHWRTFREGL-TGERNGS-PFPCFFGAESVRDGEPLYTAVP--SMSDPDALF--DLGQTLLKYLDTYQ-D-HS-E---RA------SLVTFFRPPERP-LT-----EAEYHDRLWHILQFLHVHDP---EPWPED-IPTDPDDPHWEFCFGGKPMFPTCRAPFY--DTRKSRY---CPVGLEITFQPRALFEDLNVTADTEAG-QHARDVIQGRLEEYDGVCPHADIGD-WGVEGD--REWPQYMLSS-DEN-A-SPDECPIT-----VT---R------E-
BgramDRAFT_4605_Bgra_170694643                          DWKSRII--AGKATGAAVS---AP--A-W------------------------------------------------------------LDASYATLREQV-LDPA-----YPCFFGTMAERRGEMFYSFV------NGRDLR--DLPATMQTFAELAV-R-PEYR---RN------NIAVFFEPDPEP-LSHDA-----YRTLFWGILQRLHDIDP---DPAADQ-QP-DPMDEAWEFSYAGVQMFVVCACPSF--RARHSRN---LGPGMVLLFQPRSVFV--DTITNKVIG-REARNQVRKRLETWDDIPAHPDLGF-YGDPGN--LEWKQYFLDD-ANA-P-IEERCPFL---K-RR---A------Q-
BcerKBAB4_1518_Bwei_163939507                           K-SYLLD--NEGMKNRV-D---IP--N-W------------------------------------------------------------VAKEFHDFSSVV-LEP-----TFPCYFGLTALKKNELRYSFLS------HNDWS--HLPNTMLTFLELMK-E----RPIVRR------GFFLFVEPECEE----KS--IEYYRDYFWKVLQYLHEQDN---QLWPKQ-IPKNPDHHLWEFSFGGEPIFAFGNAPAY--KQRKTRH---LGNSLVIGFQPRTIFD--GLEGDRPKG-SYSRQMVRERVEKWDQLPKHPNISH-YGDPNH--REWKQYFIGD-DIE-P-IKGKCPFH---H-RV---E------K-
bcere0006_14550_Bcer_229172344                          E-SYLLD--NEGMRTRT-D---IP--N-W------------------------------------------------------------VTKEFQNFSSVV-LEP-----TFPCYFGLTALKKNELRYSFLS------HNDWS--HLPHTMLSFLELMK-E----RPIVRR------GFFLFVEPECEE----QS--LEYYRDYFWKVLQYLHENDD---QTWPKQ-IPEDPDHYLWEFSFGGEPIFAFGNAPAY--KQRKTRH---LGNSLVIGFQPRTIFD--GLEGDRPKG-AYSRQTVRDRVEKWDQLPKHPNISH-YGDPDH--REWKQYFIGD-DVE-P-IKGKCPFL---H-KL---E------K-
B14911_27060_Bsp._89096427                              M-QLISD--ISGLKK--TD---C-----W------------------------------------------------------------EEDALRAFQAKL-GNKKD---RFPCIPATQGHALQQLRYGFAG--DPRNISSAG--ELAEMMREFTVLSR-S-FG-S---YT------SLIVFFETNEQL-AEQGT--VEEFETLFWQQMNLLAERDR---EKWPDH-IPEDPHDPLWEFCSHGEQYFMYCATPAH--VKRQSRY---F-PYFMLAITPRWVLD--EFNSAPERA-SKVKSKIRDRLAAYDSIGAHPHLNS-YGSDNN--YEWQQYFLRD-DDS---ALSKCPFH-----RM---L------A-
bcere0021_14840_Bcer_229090660                          K-SYLLD--NEGMRTRT-D---IP--N-W------------------------------------------------------------VAKEFKNFSNVV-LEP-----TFPCYFGVTALKKNELRYSFLS------HNDWS--HLPHTMLSFLELMK-E----RPVVRR------GFFLFVEPEYEE----QS--IEYYRDYFWKVLQYLHENDD---QTWPKQ-IPEDPDHYLWEFSFGGEPIFAFGNAPAY--KQRKTRH---LGNSLVIGFQPRTIFD--GLEGDRPKG-AYSRQTVRDRVEKWDQLPKHPNISH-YGDPEH--REWKQYFIGD-DVE-P-IKGKCPFL---H-KI---E------K-
BT9727_1485_Bthu_49480529                               K-SYLLD--NEGMRMRT-D---IP--N-W------------------------------------------------------------VAKEFENFSNVV-LEP-----TFPCYFGLTALKKNELRYSFLS------HNDWS--HLPHTMLSFLELMK-E----RPIVRR------GFFLFVEPECEE----QS--IEYYRDYFWKVLQYLHENDD---QTWPKQ-IPEDPDHYLWEFSFGGEPIFAFGNAPAY--KQRKTRH---LGNSLVIGFQPRTIFD--GLEGDRPKG-AYSRQTVRDRVEKWDQLPKHPNISH-YGDPEH--REWKQYFIGD-DVE-P-IKGKCPFL---H-KI---E------K-
bcere0028_14690_Bcer_229029378                          E-SYLLD--NEGMRTRT-D---IP--N-W------------------------------------------------------------VTKEFQNFSSVV-LEP-----TFPCYFGLTALKKNELRYSFLS------HNDWS--HLPHTMLSFLELMK-E----RPIVRR------GFFLFVEPECEE----QS--LEYYRDYFWKVLQYLHENDD---QTWPKQ-IPEDPDHYLWEFSFGGEPIFAFGNAPAY--KQRKTRH---LGNSLVIGFQPRTIFD--GLEGDRPKG-AYSRQTVRERVEKWDQLPKHPNISH-YGDPDH--REWKQYFIGD-DVE-P-IKGKCPFL---H-KL---E------K-
BALH_1442_Bthu_118477139                                K-SYLLD--NEGMRTRT-D---IP--N-W------------------------------------------------------------VAKEFENFSNVV-LEP-----TFPCYFGVTALKKNELRYSFLS------HNDWS--HLPHTMLSFLELMK-E----RPVVRR------GFFLFVEPECEE----QS--IEYYRDYFWKVLQYLHENDD---QTWPKQ-IPEDPDHYLWEFSFGGEPIFAFGNAPAY--KQRKTRH---LGNSLVIGFQPRTIFD--GLEGDRPKG-AYSRQTVRDRVEKWDQLPKHPNISH-YGDPEH--REWKQYFIGD-DVE-P-IKGKCPFL---H-KI---E------K-
BC03BB108_1572_Bcer_196046593                           K-SYLLD--NEGMRTRT-D---IP--N-W------------------------------------------------------------VAKEFENFSNVV-LEP-----TFPCYFGVTALKKNELRYSFLS------HNDWS--HLPNTMLSFLELMK-E----RPVVRR------GFFLFVEPECEE----QS--IEYYRDYFWKVLQYLHENDD---QTWPKQ-IPEDPDHYLWEFSFGGEPIFAFGNAPAY--KQRKTRH---LGNSLVIGFQPRTIFD--GLEGDRPKG-AYSRQTVRDRVEKWDQLPKHPNISH-YGDPEH--REWKQYFIGD-DVE-P-IKGKCPFL---H-KI---E------K-
bcere0014_14480_Bcer_229132515                          E-SYLLD--NEGMKNRV-D---IP--N-W------------------------------------------------------------VAKEFHDFSSVV-LEP-----TFPCYFGLTALKKNELRYSFLS------HNDWS--HLPNTMLTFLELMK-E----RPIVRR------GFFLFVEPECEE----KS--IEYYRDYFWKVLQYLHEQDN---QLWPKQ-IPKNPDHHLWEFSFGGEPIFAFGNAPAY--KQRKTRH---LGNSLVIGFQPRTIFD--GLEGDRPKG-SYSRQMVRERVEKWDQLPKHPNISH-YGDPNH--REWKQYFIGD-DIE-P-IKGKCPFH---H-RV---E------K-
BCZK1475_Bcer_52143762                                  K-SYLLD--NEGMRMRT-D---IP--N-W------------------------------------------------------------VAKEFKNFSNVV-LEP-----TFPCYFGLTALKKNELRYSFLS------HNDWS--HLPHTMLSFLELMK-E----RPIVRR------GFFLFVEPECEE----QS--IEYYRDYFWKVLQYLHENDD---QTWPKQ-IPEDPDHYLWEFSFGGEPIFAFGNAPAY--KQRKTRH---LGNSLVIGFQPRTIFD--GLEGDRPKG-AYSRQTVRDRVEKWDQLPKHPNISH-YGDPEH--REWKQYFIGD-DVE-P-IKGKCPFL---H-KI---E------K-
ycgG_Bsub_1805382                                       V-NGIYT--KSFLERIQEE---LP--E-W------------------------------------------------------------QRIAFELLAETL-GDDAD---TFPCIPGRQAFLTDQLRIAFAG--DPRENRTAE--ELAPLLAEYGKISR-D-TG-K---YA------SLVVLFDTPEDL-AEIIR--LKRMKSCFGAFKIDLVIKTK---KNGRKT-FRPILSIINGSFVLTASRISFCALHRGT--RQEEAGA---F-LFFVVTFQPRWVFD--DLNGSTAFG-RNMSRLIRSRLEAYDQAPIHPQLGW-YGGKDN--REWKQYFLRD-DEK---QVSKCPFS-----YL---K------N-
Bxe_C0002_Bxen_91780073                                 ---------NVEASNSADL---PA--G-W------------------------------------------------------------RDA-YRSYRETL-DRPD-----YPCFFGQAAERRGEMFYAF-------DGERPD--VARMTMRRFVELGR-D-AALA---RH------SLAMFCTPDPAL-VSHAE-----FLRRFWYILRQLHEADD---MPMRPA-DP-D--DPLWEFSFGGRKMFVVGTGPTY--RRRRSRV---VGNGIVLLFQPRELFT--DPTTGKPIS-AEVRRQIHARMLAYDAMPVHPDIGF-YGDPRN--REWKQYCLPD-DNT-P-VTGRCPFA---S-R-------------
bcere0030_15530_Bcer_229016988                          E-SYLLD--NEGMKNRV-D---IP--N-W------------------------------------------------------------VAKEFHDFSNVV-LEP-----TFPCYFGLTALKKNELRYSFLS------HNDWG--HLPNTMLSFLELMK-E----RPIVRR------GFFLFVEPECEE----KS--IEYYRDYFWKVLQYLHENDD---QTWPKQ-IPTDPDHYLWEFSFGGEPIFAFGNAPAY--KQRKTRH---LGNSLVIGFQPRTIFE--GLEGDRPKG-SYSRQMVRERVEKWDQLPKHPNISH-YGDPNH--REWKQYFIGD-DIE-P-IKDKCPFH---H-KI---E------K-
BC1001DRAFT_5695_Bsp._282889120                         DWKSRII--AGRATGAAVP---AP--A-W------------------------------------------------------------LDASYATLREQV-LDPA-----YPCFFGTMAERRGEMFYSFV------NGRDLR--DLPATMQTFAELAS-Q-PAYR---RN------NIAVFFEPDPQP-LSHDA-----YRTLFWGILQRLHDIDP---DPSADQ-QP-DPMDEAWEFSYAGVQMFVVCACPSF--RARHSRN---LGPGMVLLFQPRSVFV--DTITNKVIG-REARNQVRKRLETWDDIPAHPDLGF-YGDPGN--LEWKQYFLDD-ANA-P-IEERCPFL---K-RR---A------Q-
ED21_25953_Esp._149184227                               -------------------------------------------------------------------------MRPGLLAVEDRLAAR-LTDDFRAHIDRT---------SFPCVGAKSALAKGLLEIVIAF--DMRSAWDDL--RIHDRLLGWANAYR-D----DPEGLR------SIAVIFSQPSSL-----D--EKEFERLLWKRLQSFAEKDNWLGQPYDPA-VSPDPDDPHFSLSFGGQAFFVVGLHPA---ASRPARR---F-SHPVMVFNLHDQFE--QLRESGRY--ERMREAILERDRQLAG-DMNPMLAR-HGESSEA-RQYSGRMVGE-DWV-------CPFR-----DP---R------L-
MADE_01231_Amac_196156050                               -------------------------------------------------------------------------------------------------MEDD---------EFPCVGAKSALTKQQIHINQYD--DFRCDSNET--DILASIHKFVETFT-L----KRDMYS------SLVVTFEQPDTI-----S--ESEFDKVLWEKLQRLHEVDACL-KSWDSK-VSSDPHSAQFSLSLGGKGFFVIGLHPN---ASRKSRR---F-SNPAIVFNLHEQFE--LLRDQKRF--EAFRDHIRKRDVAYCG-SKNTLLAN-HGDSSEV-YQYSGKKHSE-NWE-------CPFK-----QV---K------H-
Rru_A3044_Rrub_83594374                                 -------------------------------------------------------------MLATSQTSQTARRPG-AAPSPSSLAAP-LVRRFRGFIEDG---------RFPCVGAKAALSRDRLAIVVAG--DIRLAGEDR--RIHAALVRMVRSYQ-H----DKAMFR------SLAVIFESPRRI-----G--EPAFERHLWARLNSLHRIDRR-HFGWSAA-VSPDPADPHFGMSFAGAGFFVVGLHPG---ASRPARR---F-VRPTLVFNMHDQFE--RLRAEGRY--DKMRATILERDRKLAG-SINPMLAA-FGERSEA-RQYSGRVVPE-GWR-------CPFH-----AE---T------T-
GFO_1924_Gfor_120436270                                 ------------------------------------------------------------------------------------MKRN-LEKIYDDWILAE---------DHPCIMAQTVFSQKNIIIENYK--RFGISGSTE--KLLKDLENYIENYD-F----SSNEFQ------SFIAVFPESEIH-----N--EDEFELVLWKQLSDLKKLDN---RPWDPS-VSRNPEDVNFSFSIAGRAFYVVGMHPE---SSRISRR---S-PYPSIAFNLHDQFE--KLREMGVY--QNVRNKIRDRDMGLQG-SINPMLED-FGENSEA-RQYSGKSTDK-DWT-------CPFH-----Q-------------
Plav_3248_Plav_154253686                                ---------------------------------------------------------------------------------MTQGKHS-LCEEFRAFVRQT---------EFPCVGAKSALSRDQLVLMTAA--DIRCPADDR--KIVDGIYAFLREHE-A----RPRLFT------SFAVVFQGPQDL-----G--EMQFEQHLWERLSALHKLDAE-DFAWSDE-VSADPNSPDFGFSLGGHAFFIVGLHPG---SSRTARR---L-PHPAIVFNLHEQFQ--NLRQNGQY--NRIKETIIHRDAKLDG-APNPMIAE-HGTVSEA-RQYSGRPVSG-RWR-------CPFA-----R-------------
AmacA2_010100018851_Amac_239996513                      ---------------------------------------------------------------------------------MSLLKPN-LTERFLSFVGEA---------DFPCVGAKTALNKSQIHVNQYD--DIRCPLNET--DMLASIHQFVESFD-L----ERDMYS------SLVATFELPDTI-----S--ETEFDRVLWEKLQRLHEIDACL-KPWDSK-VSSDPHNAQFSLSLGGKGFFVVGLHPN---ASRKSRR---F-PYPAIVFNLHEQFD--MLRKQKRF--EAFKDHIRKRDTAYCG-SKNELLAN-HGDSSEV-YQYSGVKHSE-NWE-------CPFK-----RI---K------H-
GFO_0001_Gfor_120434373                                 -----------------------------------------------MKENMGTIEKKNR-----------------KRCLLKDENSK-IRKEFENFILVK---------DHPCIMAQTIFSMDQVVLNSYS--DFGSLKTAK--NLMKDLENYIAEYD-F----KSNDFK------TFLAVFPDSPEY-----S--EIDFEKILWEQLQNLHESDN---KDWDHE-VSQDSSEENFSFSLGGKAFYIVGLHPN---SSRIARR---S-PYPAIAFNLHWQFE--KLREMGTY--ETVRDRIRQRDIELQG-NMNPMLED-FGASSEA-KQYSGRKVGK-EWK-------CPFH-----KN---N--------
mma_1650_Jsp._152981633                                 ----------------------------MSTLADKTESNPFDSAQALENSNYGAFVDKKLVAGEH------------LDEKPAPLLGF-IHDSFRALILNP---------QFSCVGAKSAIQNGGYRIGHYD--ELATPTATA--GLAHDLYHFLSEQP-A----LEGEFS------TFVATFSQPVIH-----S--EEQFEKLLWEQLQLLHEADAKV-HQWDPN-VSDDPANPHFAFSFAEQSFFVVGIHPA---ASRYTRK---F-AWPALVFNLHAQFE--KLREDGKF--ERMQEVIRSREQALQG-SINENLSE-HGTISDA-RQYAGRSVAD-DWE-------CPFS-----SK---N------K-
Patl_0917_Patl_109897242                                ----------------------------------------------------------------------------------MNSQGE-RPSSIENFIKQD---------GFPCVGAKIALNKDQIVTRDFG--DIFSNQNNQ--NILDELYQFITIYN-S----SKPLYF------SFVATFKNSILT-----S--EKEFEDALWSKLQNLYNRDS-MTHTWDRR-VSDNPDDSEFSFSLGGEAFFIIGLSPF---AERRARR---F-EHPTIVFNLHSQFQ--ILKETNKF--TALRDRIRTNEEVFSG-SPNPNLYD-HGELSEA-RQYSGRAVSN-SWR-------CPFS-----TR---K------K-
RvanDRAFT_3140_Rvan_283824582                           ----------------------------------------------------------------------------------MTEITP-KHNSVDYFVSSA---------SFPCVAAKSASARGNITEMTYG--DMNEATKDA--AILDDLGEFIAT---A----HDESLR------SFVAAFE---------------AFEEAMWERLQALHDLDTRF-CAWDTR-VSCDPASPDFSFSLRGEAFFVIGLHPN---ASRLSRR---Y-FRPALVFNLHEQFE--TLRRQGRY--DRMRDTIRKRDAALCG-SANPALRN-FGDRSEA-ANTADAPRLM-TGD-------APFG-----LD---A------R-
M446_2566_Mesp_170740784                                -----------------------------------------------------------------------------MTFPEDDADHP-LANRFRAFLRRP---------AFPCVGAKSALQRGQLRLVVAG--DLAAGRDDD--RIYPALLAFVARTR-E----APGLFQ------SFAVLFEGPTSL-----T--EEAFETHLWARIQALSDRDSRVGLPYDPR-VRADPDDPHFALSLGGEAFFVVGLHPG---ASRPARR---F-EAPALVFNLREQFV--RLRAEGRY--ESLRAAILDRDLAWTG-SINPMLAR-HGETSEA-RQYSGRLVGE-AWT-------CPFR-----RR---E------T-
Mpop_2379_Mpop_188581631                                -----------------------------------------------------------------------------MLLPTDDTGHP-LAERFRHFIRQQ---------PFPCVGAKSALSRGQLKVLVAR--DIASDVDDA--RIYPALLAFICRYR-A----RRDQFQ------SFAVLFEDARPL-----S--EEAFEAALWKRMQSLSDRDSGLGHPQDSR-VSADPDDPHFSMSLGGEGFFIVGLHPG---ASRKARR---F-EHPVLVFNLHDQFE--RLRAEGRY--DRLRDSIVERDVAWAG-SVNPMLAQ-HGERSAA-RQFSGRAVPD-DWV-------CPYR-----RR---E------G-
Mchl_2686_Mchl_218530638                                -----------------------------------------------------------------------------MLLPTDDTGHP-LAERFRHFIRQQ---------PFPCVGAKSALSRGQLKVLVAR--DVASDSDDA--RIYPALLAFICRYR-A----RRDQFQ------SFAVVFEGPHHL-----S--EEAFEAALWRRMQSLSDRDSELGHAQDPR-VSANPEDTHFSMSLGGEGFFIVGLHPG---ASRKARR---F-EHPVLVFNLHDQFE--RLRAEGRY--DRLRDSIVERDVAWTG-SINPMLAQ-HGERSAA-RQFSGRAVPD-DWV-------CPYH-----RQ---E------G-
Mext_2464_Mext_163851887                                -----------------------------------------------------------------------------MLLPTDDTGHP-LAERFRHFIRQQ---------PFPCVGAKSALSRGQLKVLVAR--DVASDSDDA--RIYPALLAFICRYR-A----RRDQFQ------SFAVVFEGPHHL-----S--EEAFEAALWRRMQSLSDRDSELGHAQDPR-VSANPEDTHFSMSLGGEGFFIVGLHPG---ASRKARR---F-EHPVLVFNLHDQFE--RLRAEGRY--DRLRDSIVERDVAWTG-SVNPMLAQ-HGERSAA-RQFSGRAVPD-DWV-------CPYH-----RQ---E------G-
METDI3192_Mext_254561605                                -----------------------------------------------------------------------------MLLPTDDTGHP-LAERFRHFIRQQ---------PFPCVGAKSALSRGQLKVLVAR--DVASDGDDA--RIYPALLAFICRYR-A----RRDQFQ------SFAVVFEGPHHL-----S--EEAFEAALWRRMQSLSDRDSELGHAQDPR-VSANPEDTHFSMSLGGEGFFIVGLHPG---ASRKARR---F-EHPVLVFNLHDQFE--RLRAEGRY--DRLRDSIVERDVAWTG-SVNPMLAQ-HGERSAA-RQFSGRAVPD-DWV-------CPYH-----RQ---E------G-
MexAM1_META1p2427_Mext_240139010                        -----------------------------------------------------------------------------MLLPTDDTGHP-LAERFRHFIRQQ---------PFPCVGAKSALSRGQLKVLVAR--DVASDSDDA--RIYPALLAFICRYR-A----RRDQFQ------SFAVVFEGPHHL-----S--EEAFEAALWRRMQSLSDRDSELGHAQDPR-VSANPEDTHFSMSLGGEGFFIVGLHPG---ASRKARR---F-EHPVLVFNLHDQFE--RLRAEGRY--DRLRDSIVERDVAWTG-SANPMLAQ-HGERSAA-RQFSGRAVPD-DWV-------CPYH-----RQ---E------G-
DR_0614_Drad_15805641                                   ---------------------------------------------------------MQP-----------------TRGPVSARAAE-LNAAFREKILAP---------DFSCVAAKASMNTSAYALGVYG--ELGSLSATX--GLAHDLARFVQDQD-D----LGSDFT------SFIATFDSPEGM-----T--EPQFEERLWQQLRALHQLDT---APYSEE-VSADPHSNKFGFSFAGRGFFIIGLHPG---SSRVARA---F-PAPALVFNAHRQFQ--KLRDTGRF--ERMQQTIRSRELKLQG-SLNPNLAN-YGEAPEA-RQYSGRAVEP-GWV-------APFP-----NT---PTAGRCPF-
ShygA5_010100002160_Shyg_256773741                      ------------------------------------------------------------------------------------MTVD-ARRDVEEFMLGE---------RFACLAGRSAWRRGGITHRHYD--LLGGEDSAR--LLALDLAEFVSSAD-W----SARTFT------SFIATFEQPRGV-----D--ELRFEELLWQQLQLLHEQDADF-HHWAEG-YSSDPQSPEFAFSVAGHPFFVIGLHET---HRRFGRR---P-PFPMLAFNSHEQFD--RIKDAGMW--DRLAEKIRKQDIQLQG-DINPNLYE-YEELSET-RRYSGRPKPA-DWQ-------CPFA-----AR---E------S-
JNB_14243_Jsp._84497361                                 ---------------------------------------------------------MKQ-----------------TKNPSTVPTEDDVLAQLAPWVLDP---------EYPCLGARAVFSRERAHVVTTG--RLGSASSSF--DVYRALRDF-GEEA-D----PSQGFT------SLIAAFGGEPPL-----T--EFTFEDRLWTTLQHLHDFDD---TPWNSE-VSDDPADVDFSFSVGGVAYFVIGLHPH---ASRDARR---T-PWPVLVFNLHSQFE--ELKASGRY--DRMRDLIRGRDLHLQG-SVNPMVAD-HGVISEA-RQYSGRAVSA-EWQ-------APLE-----QA---SAT---PL-
Mrad2831_4911_Mrad_170751294                            -----------------------------------------------------------------------------MQLPTNDEHHP-VAARFRDFIQHA---------PFPCVGAKSALSRGQMKIVVAR--DITSDRDDT--RIYPALLAFICRYR-A----QPSLFQ------SFAVLFEGPRTL-----T--EEAFEAALWRRVQSLSDTDQRLGQAYDPR-VAADPDDPHFSLSLGGQGFFLVGLHPG---ASRRARR---F-ETPALVFNLHDQFE--RLRAEGRY--ERLRTAIVDRDAAWTG-TINPMLAQ-HGESSAA-RQFSGRAVPD-DWA-------CPFR-----RG---E------A-
Mpop_0164_Mpop_188579443                                -----------------------------------------------------------------------------MQLPRDDTNHP-QAEAFRAFIRDA---------GFPCVGAKSALTKGQMRVLVAR--DITSAWDDM--RIYPALMAFAARYR-R----QPDLFQ------SFAVVFEGPSDL-----D--EEGFERHLWNRVQSLADKDAWLGHPWDGR-VAPEPDSPHFSLSFAGEAFFVVGLHPN---ASRPARR---F-SSPALVFNLHAQFE--QLREAGRY--EKLRSSILQRDEALAG-SVNPMLAR-HGELSEA-RQYSGRVVDE-AWR-------CPFR-----PR---A------T-
RPB_1618_Rpal_86748742                                  -----------------------------------------------------------------------------MTPPS-------LEDEFRNFIHAA---------DFPCVGAKSALSKGTLHVLVAR--DIRSNWDDR--RIYDGITRVVRDYR-E----NRALFQ------SFAVIFEGPTDI-----D--EQAFEKFLWARAESLTNKDTWLGRPHDER-VSSDPGDPHFSLSFGEEAFFIVGLHPN---ASRPARR---F-ERPVLVFNLHDQFE--QLREMGRY--ERLRAKIIERDVALAG-SPNPMLAR-HGETSEA-RQYSGRAVAEQEWV-------CPFN-----PV---D------R-
Mrad2831_1830_Mrad_170748248                            -----------------------------------------------------------------------------MHLPRDDREHP-RAEAFRDFIRNP---------PFPCVGAKSALSRGRMRIVVAR--DITSGWDDM--RIYPALLAFVAQYR-A----QPDLFQ------SFAVVFEGPSDL-----S--EEAFERNLWARAQSLTDKDAWLGQPHDAR-VAEDPDNPHFSLSFGGEAFFVVGLHPQ---ASRPARR---F-SSPVLVFNLHAQFE--RLRAEGRY--EKLRAAILERDEALAG-SVNPMLAR-HGEASEA-RQYSGRAVDA-DWR-------CPMK-----GR---P------A-
ZPR_0378_Zpro_295132257                                 -------------------------------------------------------------------------------MTSDKKATK-VYNSFQDFIITN---------DHPCIMAKTVFSMNLVNLRTYN--NLGNLNQTK--ELYKDLKAYIKNYD-F----ESNQFE------TFIAVFPNSPEF-----T--EIQFEEQLWIQLNQLNAVDE---YDWDPS-VSNNPNDDNFSFSIAGKSFYIVGLHPN---SSRKARR---S-PYPAIAFNLHWQFE--KLRKMGTY--DRVKTSIRERDEQFSG-STNPMLKD-FGSQSEA-RQYSGRKVGE-LWE-------CPFK-----K-------------
FIC_00380_Fbac_255534538                                -----------------------------------------------MAVACSFKIQFME-----------------TSTPSTFDLNT-IDRDIQSFVVDG---------GHPCIMAKSVFKMKNYDLHAYA--DMQGETQIK--ELLADLETYLQDAG-R----DSMNFR------SFVAVFPNNKFT-----D--EISFENALWATLQQLHEADE---MPWDES-VSRNPDDAEFSFSLLGKAFYVIGLHTQ---SSRMARQ---A-PYTTLVFNLHQQFE--KLREIGTY--QNVRDMIRKNDEKLQG-SINPVLKD-FGDDSES-RQYSGRNVES-NWK-------CPFH-----HK---HN-------
NT01EI_1675_Eict_238919576                              ----------------------ME----Y----------------A-----------IKQ-----------------------------IINDFESFVKSK---------SFCCIGARRAQAENNLLY-FPS---MHPIEDIS--NVYLFLEDFACNQI-K----NKRFYS--AFY-SAVVFFENKKIA-----S--EDDFENFLWEVLKELNSIDSQK-YQWASG-YSRNTRDDNFAFSIAEEAFFIVGLHPF---AHRLSRN---F-KCPALVFNHHEIFS--ALRLSGHF--DKYKAVIRKNEMHIQG-SVNRQLSD-FGKNQK---------LRN-----------MPVL---T-KI------FLS-K-
Psyrpa2_010100013775_Psyr_289648803                     V-RHLLE--SDTALE-------MG----W------------------------------------------------------------RVQAYRQFEQVL-SDK-----GFPCLFGRRANKSGSCLLLFIP-----CENEQQ--ALRDGMEAYVKFVN-D-TPLEDRLFN------PLIVIFEKTDFN-----T--LAEEQAYAWATLQHLHDGDR---TPWPAK-ACTDPEVFEWTYHFAGLPMFINMSFPRH--SAMKSRS---LGGHIVFVVNPRENFDE-VASAETESG-RKVTEKIRQRIADYNNGVVPDTLGF-FGDRSS--LEWKQYQLYE-EGG-L-SLSRCPLH---I-KV---D------K-
PSPTOT1_0894_Psyr_213968587                             V-RHLLE--SDTALE-------MG----W------------------------------------------------------------RVQAYRQFEQVL-SDK-----GFPCLFGRRANKSGSCLLLFIP-----CEHEQQ--ALRDGMEEYVKFVN-D-TPLEDRLFN------PLIVIFEKNDFN-----S--LAEEQAYAWATLQHLHDGDR---SPWPAK-ACKDPEVFEWTYHFAGLPMFINMSFPRH--SAMKSRS---LGGHIVFVVNPRENFDE-VASAETESG-RKVREKIRQRIADYNNGVVPDTLGF-FGDRSS--LEWKQYQLYE-EGG-L-ALSRCPLH---I-KV---D------K-
PSPTO_1884_Psyr_28869088                                V-RHLLE--SDTALE-------MG----W------------------------------------------------------------RVQAYRQFEQVL-SDK-----GFPCLFGRRANKSGSCLLLFIP-----CEHEQQ--ALRDGMEEYVKFVN-D-TPLEDRLFN------PLIVIFEKNDFN-----S--LAEEQAYAWATLQQLHDGDR---SPWPAK-ACTDPEVFEWTYHFAGLPMFINMSFPRH--SAMKSRS---LGGHIVFVVNPRENFDE-VASAETESG-RKVREKIRQRIADYNNGVVPDTLGF-FGDRSS--LEWKQYQLYE-EGG-L-ALSRCPLH---I-KV---D------K-
PsyrptA_020100006391_Psyr_257482863                     V-RHLLE--SDTALE-------MG----W------------------------------------------------------------RVQAYRQFEQVL-SDK-----GFPCLFGRRANKSGSCLLLFIP-----CENEQQ--ALRDGMEAYVKFVN-D-TPLEDRLFN------PLIVIFEKTDFN-----T--LAEEQAYAWATLQHLHDGDR---TPWPAK-ACTDPEVFEWTYHFAGLPMFINMSFPRH--SAMKSRS---LGGHIVFVVNPRENFDE-VASAETESG-RKVREKIRQRIADYNNGVVPDTLGF-FGDRSS--LEWKQYQLYE-EGG-L-SLSRCPLH---I-KV---D------K-
ETA_27510_Etas_188534871                                V--IFSH--AEVIES-------LHNFSEW------------------------------------------------------------KRKAWRQFSIKL-HDS-----EFPCIFSKSAWKAKSIKFVFCE-----KRKDSEYLDFLHGLVSYSDYIN-D-TPLSKRLLS------PLVVFF-SPEYY-----I--NKNQHETGWEALNWAHARDS---KPWPEN-ISVSPEDAEWTFYFNGIQFFINMSTQNH--RILRNRN---LGAHLTFVINARENFDA-VANGNTKAG-RQLREHIRERVREYNGGVFPSELGF-YGDDAN--LEWKQYQLEE-SGT-E-RPQQCPFR---H-RKTT----------
PputW619_1901_Pput_170721085                            L--ELAA--EHVRNT-------QH----W------------------------------------------------------------THKTIQHFRNIL-ANP-----DFPCLFGRKAVAGESCHILFAR-----AEQLAD--DIAQGLAEYVRTIA-P-VPVKQRIGS------PLVVFLETAADS-----S--LAEQQALAWKVLRGVHARDP---HPWPQA-IPADPHDSGWSFCYAGMALFINMNFPGH--HQMKSRN---LGNHITFVINPRENFDE-VANADTESG-KRIRARIRERVQHYNDGVMPDSLGF-FGQADN--FEWKQYQLQE-AGS-L-NPSRCPFH---A-PV--HA------T-
PputGB1_1995_Pput_167033003                             L--ELAA--EHGPNM-------QH----W------------------------------------------------------------TYKTIEHFRSVL-ANP-----EFPCLFGRKAVNGETCHILFAR-----AEQLAD--DIAQGLADYVRTVT-Q-ITAKQRIGS------PLVVFLETPAGS-----N--LAEQQALAWKVLRGVHARDP---HPWPQG-IPTDPDDSGWSFCYAGMPLFINMNFPGH--QQMKSRN---LGQHVTFVINPRANFDE-VANANTESG-KRIRERIRERVHHYNDGVVPDTLGA-FGDADN--YEWKQYQLQE-AGS-L-NPARCPFH---A-HAAHLA------T-
PP_2389_Pput_26989113                                   L--ELAA--EHGHTM-------QH----W------------------------------------------------------------TFKTIEHFRSIL-ANP-----EFPCLFGRKAVNGETCHILFAR-----AEQLAD--DIAQGLANYVRTVA-P-IAPKQRIGS------PLVVFLETAAGS-----S--LAEQQALAWKVLRGVHARDP---HPWPQG-IPTDPDDTGWSFCYAGMPLFINMNFPGH--QQMKSRN---LGQHITFVINPRENFDE-VANANTESG-KRIRERIRERVHHYNDGVVPDTLGF-FGDTDN--YEWKQYQLQE-AGS-L-NPSRCPFH---A-HAAHQP------T-
PSEEN3369_Pent_104782413                                L--ELAD--EHVQNR-------HH----W------------------------------------------------------------THKTIEHFKETL-AHP-----DFPCLFGRKAVTARTCHIIFAR-----AQQLAD--DIARGLADYVRTVE-P-LPIKQRIGH------PLLVFLETAPNT-----T--LSDQQALAWDVLRQVHARDP---QPWPEA-VPQDPHDAHWSFCFAGMPLFINMSFPGH--LQMKSRN---LGKHIAFVINARESFDE-VASAQTESG-QRIRARIRDRVRHYNDGVMPDTLGF-FGHGDN--FEWKQYQLQE-PGS-L-NPARCPFH---T-----QA------T-
Psyrpo1_010100009961_Psyr_237799153                     V-RHLLE--SDTAQE-------MG----W------------------------------------------------------------RVQAYRQFEQVL-SDK-----GFPCLFGRRANKSGSCLLLFIP-----REHEQQ--ALRDGMEEYVRFVN-D-TPLEDRLFN------PLIVIFERNDFN-----S--LAEEQAYAWATLQHLHDDDR---SPWPAD-ACSNPEIFEWTYHFAGLPMFINMSFPRH--SAMKSRS---LGGHIVFVVNPRENFDE-VASAETDSG-RKVREKIRQRVADYNNGVVPDTLGF-FGDRNS--LEWKQYQLYE-EGG-L-ALSRCPLH---I-KV---D------K-
FG11502.1_Gzea_46139975                                 H-GGIFT--RDDVESQF-D---DN--S-W------------------------------------------------------------QGLAYHDFRSTL-LAKGRAMKTFPCVYATMGFRSGDHRFVFLESDNPSEPRNVR--KVATALAEYLRIST-S-LG-P---NT------SLVIIGAPSEKE-RT-----IEEYNHTFWDMLRGLRICDP---KAWPKD-IPQDTEDAKWTFCFSGQPIFPVMLTPAH--QERWSRH---M-SVPLIALQPKWVLD--NLLQTPEKR-KSAQSKVRGLLQKYDTIGISPDLTD-YGTTGT--SEIRQLCLQD-KNE---SVQ-CPYR-----NF---D------S-
MGG_11305_Mory_145614158                                K-VELLD--RYQVELTY-P---QG--T-W------------------------------------------------------------QRQAYDDFVGVL-ISPSK---TFPCVYGTKGFKANELDFVFLDSEELGSAATAK--LGAKAILEYHQVLE-G-RG-R---NI------SLVMLCPPPERE-RS-----VHEYHDAFWAFLHRLRQLDP---KPWPAQ-IPRATGHVKWCMNFDGVEAFFAVLTPAH--RQRLSRH---A-PNFAMVYQPRYIFD--AVFKNARYR-ESATKMIRGLVDRYDEIPHSPEISD-YGLPGT--TESRQYFLLD-ENV---PSK-CPYE-----SL---D------D-
DS26_Mpin_154091375                                     S-ARLYT--KEQVESNF-D---MN--A-W------------------------------------------------------------QRLAYEEFKTTI-LAKGTGLSTFPCVYATMGYRAGDHRYVFLESDDPSEPRNVR--IVAPALRAYLRMSS-T-LG-D---NT------SMVIMAAPTEGDPKS-----VEEYNHSFWEMLRGLRIWDA---KPWPKE-FPQDTQNEKWTYCFDGTPLFPVALTPAH--QKRWSRH---A-PVPLIALQPKWVLD--KLLSTTEKR-EAATGKVRKLLKQYDQTEISPDLTT-YGDVGT--SEVHQLCLRD-ENE---TAD-IPYQ-----DF---D------KG
Veis_0872_Veis_121607862                                T-SVLSR--RRDIAERY-A---KT--S-W------------------------------------------------------------EQILFSEFSTSL-ESSNR---PFPCIFGVRGFKLDQLRYVFQ------ENLDLD--LTSAALQEFVRDAR-S-FG-P---NT------SLVIF-TRPEEI-KS-----IDAYQEEFWKILKGLADRDH---TPWPEH-MPTEITHPEWEFCFAGEPVFVVCNTPAH--IFRQSRR---A-SSFMLTFQPRWVFD--KILGTEKST-QTAFGAVRKRIAHYDFLPVSPKLGK-YGHPGV--LESEQYFLDD-RNQ---GGSSCPFQ-----AL---G------E-
BBta_5822_Bsp._148257087                                R-LFL-R--KDEVRASY-R---VS--S-W------------------------------------------------------------QSVLFSEFEAQM-SSDAR---PFPCIFGVAGYRQDQLRYLFL------DPLDTD--ALGKQLALFVSESR-S-HG-P---NT------SLVLF-TRPRPV-QT-----LDAYYKKFWRILDQLARIDK---QPWPAH-IPQTIDDPMWEFSFAGEPIFVVCSTPAH--VMRQSRR---S-SAFMLTFQPRWVFE--KILGTDRAA-AKAFGEVRKRLAPFDTTTASPLLGR-YGDPEG--REYKQYFLYD-DNE---PPPACPFH-----RL---A------Q-
RBY4I_3722_Rbac_254467109                               T-KIIYS--RVEIDSAF-D---AT--T-W------------------------------------------------------------QKKIFNELATNF-RSRSR---LFPCTFGVAGFEADQLRFAFS------ENMDPG--EVSSALKCYLKDAK-S-FG-K---NT------SLLVL-SRPGPI-QS-----LEHYRARFWSTLDGIHQTDD---TEWPET-IPAQIDSAGWEWCFGGEQIFVVCNTPAH--VNRQSRR---F-SSFMLTFQPRWVFN--GILDTRETA-EKATSKIRSRILQYDLINPSEDLGL-YGDPDN--REFAQYFLDD-ENR----AATCPFH-----SF---T------K-
MCYG_06782_Mcan_238844301                               S-SKILK--RKELETTF-G---PT--T-W------------------------------------------------------------QRQSYDAFQAAV-CSTDP--VAFPCVYATKGFKAKEHRYIFLDSEDMNNKKNID--SLAAALKEYLTTPQ-SELG-P---NT------SLVVLFPIVDSR-LT-----ARDYHQKYWDCLRALRKVDT---KAWPAN-IPTDTDTPLWKFCFNGEPVFSAAMTPSH--EKRRSRY---A-PCFCIVFQPNFVFD--ILFATELKK-KAAISKVRGLLADYDEVPISPELKN-YGDVTG--RESKQYFIMD-ENH---SSPPCPYS-----SL---D--------
TRV_07646_Tver_291181972                                G-SKILK--RKEVEETF-G---PT--T-W------------------------------------------------------------QRQSYDAFQAAV-CSTDP--VAFPCVYATKGFKAKEHRYIFLDSEDMNNKKNIA--TLAAALKEYLTTPQ-SELG-P---NT------SLVVLFPVINSQ-LS-----PQEYHQKYWDCLRALRKVDT---KPWPSH-IPTDTDTPLWKFCFNGEPVFSAAMTPSH--EKRRSRY---A-PCFCIVFQPNFVFD--ILFATDLKK-KAAISKVRGLLADYDEVPISPELKN-YGDATG--RESKQYFIMD-ENQ---SSPPCPYT-----SL---D------V-
ARB_07020_Aben_291178279                                G-SKILK--RKEVEETF-G---PT--T-W------------------------------------------------------------QRQSYDAFQAAV-CSTDP--VAFPCVYATKGFKAKEHRYIFLDSEDMNNKKNIA--TLAAALKEYLTTPQ-SELG-P---NT------SLVVLFPIINSQ-LS-----PQEYHQKYWDCLRALRKVDT---KPWPSH-IPTDTDTPLWKFCFNGEPVFSAAMTPSH--EKRRSRY---A-PCFCIVFQPNFVFD--ILFATDLKK-KAAISKVRGLLADYDEVPISPELKN-YGDATG--RESKQYFIMD-ENQ---SSPPCPYT-----SL---D------V-
FRAAL4158_Faln_111223559                                A-TDALR--EEILDLAIGP---MP--G-W------------------------------------------------------------GTGCARDIMATL-KSDSE---PFPCVFAVAAAHRGGLRFGFVE--DLRDERTWS--VLPDILATYLSVYQ-S-IS-R---DT------SLVVFFGSGEGA-AGQDNDDVLSYEQRFWSILSYLHDRDG---EPWPSD-IPTDTDDPGWEFSFRGTPIFVVCNTPAH--RRLRSRS---G-PVFTITFQPRWVFT--GLEPTSPRG-AAARKVIRERLARTDEVDSTPLLGS-YGDPAN--REWKQYYLRD-DAS---VADSCPFHAGAGRRS---A------D-
FraEuI1cDRAFT_2091_Fsp._280961469                       L-EDAFH--EEALDLAVGP---MP--A-W------------------------------------------------------------GAGCARDMITTL-RSDAE---PFPCVFAVAAANSGGLRFGFVD--DLDDERTWS--VLPDILASYLATYQ-S-IS-R---NT------SLVVFFGSGNGT-AGQESDDVLGYERRFWSLLRYLHDSDG---EPWPSD-IPTDTDDPAWEFSFRGSPIFVVCNTPAH--RRLRSRS---N-PVFNITFQPRWVFE--GLAPTTPRG-AAARKVIRERLRRMDEVDSTPLLGS-YGDPAN--REWKQYFLRD-DAT---VAEKCPF------RA---K------W-
consensus/100%                                          .................................................................................................................C..u.................................h..h.......................sh.....................b..............s......................h....................b.s...........h.h.....h...................l................................................hsh....................
consensus/95%                                           .............................................................................................h..h..............aPCh.u..u.......h..................h...h..a.......................shhhhh.................bp..hW..hp.....D.......s....s.ps.p..a.hsh.s..hF.....P......b.sR..........hshp.p..h...................lb.b...hp..s....l...aG.......p...b.h.............hPh....................
consensus/90%                                           .............................................................................................h..h..............FPCh.u..u.......h.hh...............h...h..a.p..p..................shhhhh.................ap..hW..Lp..p..D......hs.p.hs.ss.p..aphsh.s.shFh.s..P......RbsR..........hshp.+..hp...h...........p..lbpR...hs..s.ps.l...aG...p...pa..b.l.c.p.........CPh....................
consensus/85%                                           .............................................................................................ap.h...h..........FPCh.u..ubp.sphbh.hh.........s....ph...h.pa.p..p......p...b.......shhlhhps...............ap..hWp.Lp.lpp.D......Ws.p.hs.sP.p..apash.ubshFh.s..P......RboR....h.....hshp.+..hp...h.sp........pp.lbpR...as..s.ps.l...aG...p..bpap.b.l.-.p.p.......CPa....................
consensus/80%                                           ............................a..............................................................p.ap.F...h.........sFPCh.u.pubp.sphbh.hh........ps....pl...hbpa.p..p.p....p...b.......shhlhhps...............acp.hWp.Lp.Lpp.D......Ws.p.ls.-P.p..WpasasGbshFh.s..Ps....pRboRp...h.s.hhlshpP+..Fp...l.ssp.......pp.lRpRl..as..s.pPpl...aG..pp..bEap.hhl.-.c.p.......CPa......p.............
consensus/75%                                           ....h.......h...............W..............................................................p.ap.F.p.h.........sFPCh.u.puhppsph+hsahs.......ps.p..pl..shbpahp..p.p....p...bp......uhhlhhcs..p......s...p.acp.hWphLp.Lpp.D....psWPpp.lspDPpc..WpFsFsGcshFhhs.sPua...pRboRp...h.s.hhlshpPR..Fp...lpssp.bu..b.+p.lRpRl..aD..s.pPpls..aGp.sp..bEapQYhl.-.-.p......pCPF......p...........p.
consensus/70%                                           ....lhp..pp.h..........s....W..............................................................p.appF.p.h..p......sFPCh.ubpuhppspl+asFhs......pps.p..cls.shbpalc..p.p....p...bp......uhhlhhcs.ppb.....s...c.acpbhWphLp.Lpc.Dp...psWPpp.lPpDPpc..WpFsFsGEshFhhs.sPua..ppRcoRp...h.sshhlshpPR.lF-..slpssp.bu..b.+p.lRpRl..aD.hs.pPslup.aGs.sp..bEWpQYhl.D.-.p.....spCPF......c...........p.
```


---


8. List and domain architectures of MPRF-like acetyltransferases in Bacteria and Eukaryotes
  


```
#1;7xTM+MprF_like-GNAT+K-tRS
240168025    7xTM+MprF_GNAT+OBfold_anticodonB+K-tRS                                                                                                                                                                                                                                                                                                      lysS                       1103   Mycobacterium kansasii ATCC 12478                              bacteria>actinobacteria                         lysyl-tRNA synthetase [Mycobacterium kansasii ATCC 12478].           
296439737    7xTM+MprF_GNAT+OBfold_anticodonB+K-tRS                                                                                                                                                                                                                                                                                                      lysX                       1105   Mycobacterium marinum M                                        bacteria>actinobacteria                         RecName: Full=Lysylphosphatidylglycerol biosynthesis bifunctional    
183982461    7xTM+MprF_GNAT+OBfold_anticodonB+K-tRS                                                                                                                                                                                                                                                                                                      lysS                       1097   Mycobacterium marinum M                                        bacteria>actinobacteria                         lysyl-tRNA synthetase 2 LysX [Mycobacterium marinum M].              
296439734    7xTM+MprF_GNAT+OBfold_anticodonB+K-tRS                                                                                                                                                                                                                                                                                                      lysX                       1105   Mycobacterium ulcerans Agy99                                   bacteria>actinobacteria                         RecName: Full=Lysylphosphatidylglycerol biosynthesis bifunctional    
118617265    7xTM+MprF_GNAT+OBfold_anticodonB+K-tRS                                                                                                                                                                                                                                                                                                      lysS                       1097   Mycobacterium ulcerans Agy99                                   bacteria>actinobacteria                         lysyl-tRNA synthetase [Mycobacterium ulcerans Agy99].                
296038682    7xTM+MprF_GNAT+OBfold_anticodonB+K-tRS                                                                                                                                                                                                                                                                                                      lysS                       1121   Rhodococcus equi ATCC 33707                                    bacteria>actinobacteria                         lysine--tRNA ligase [Rhodococcus equi ATCC 33707].                   
253799320    7xTM+MprF_GNAT+OBfold_anticodonB+K-tRS                                                                                                                                                                                                                                                                                                      lysS                       1174   Mycobacterium tuberculosis KZN 1435                            bacteria>actinobacteria                         lysyl-tRNA synthetase 2 lysX [Mycobacterium tuberculosis KZN 1435].  
15841096     7xTM+MprF_GNAT+OBfold_anticodonB+K-tRS                                                                                                                                                                                                                                                                                                      lysS                       1185   Mycobacterium tuberculosis CDC1551                             bacteria>actinobacteria                         lysyl-tRNA synthetase [Mycobacterium tuberculosis CDC1551].          
31792827     7xTM+MprF_GNAT+OBfold_anticodonB+K-tRS                                                                                                                                                                                                                                                                                                      -                          1172   Mycobacterium bovis AF2122/97                                  bacteria>actinobacteria                         lysyl-tRNA synthetase [Mycobacterium bovis AF2122/97].               
289757744    7xTM+MprF_GNAT+OBfold_anticodonB+K-tRS                                                                                                                                                                                                                                                                                                      TBEG_02947                 1172   Mycobacterium tuberculosis T85                                 bacteria>actinobacteria                         lysyl-tRNA synthetase [Mycobacterium tuberculosis T85].              
121637548    7xTM+MprF_GNAT+OBfold_anticodonB+K-tRS                                                                                                                                                                                                                                                                                                      lysS                       1172   Mycobacterium bovis BCG str. Pasteur 1173P2                    bacteria>actinobacteria                         lysyl-tRNA synthetase [Mycobacterium bovis BCG str. Pasteur          
15608778     7xTM+MprF_GNAT+OBfold_anticodonB+K-tRS                                                                                                                                                                                                                                                                                                      -                          1172   Mycobacterium tuberculosis H37Rv                               bacteria>actinobacteria                         lysyl-tRNA synthetase [Mycobacterium tuberculosis H37Rv].            
289753728    7xTM+MprF_GNAT+OBfold_anticodonB+K-tRS                                                                                                                                                                                                                                                                                                      TBGG_00844                 1172   Mycobacterium tuberculosis EAS054                              bacteria>actinobacteria                         lysyl-tRNA synthetase [Mycobacterium tuberculosis EAS054].           
289447250    7xTM+MprF_GNAT+OBfold_anticodonB+K-tRS                                                                                                                                                                                                                                                                                                      TBNG_02145                 1172   Mycobacterium tuberculosis CPHL_A                              bacteria>actinobacteria                         lysyl-tRNA synthetase 2 lysX [Mycobacterium tuberculosis CPHL_A].    
254775578    7xTM+MprF_GNAT+OBfold_anticodonB+K-tRS                                                                                                                                                                                                                                                                                                      lysS                       1085   Mycobacterium avium subsp. avium ATCC 25291                    bacteria>actinobacteria                         lysyl-tRNA synthetase [Mycobacterium avium subsp. avium ATCC         
118467307    7xTM+MprF_GNAT+OBfold_anticodonB+K-tRS                                                                                                                                                                                                                                                                                                      lysS                       1075   Mycobacterium avium 104                                        bacteria>actinobacteria                         lysyl-tRNA synthetase [Mycobacterium avium 104].                     
296439739    7xTM+MprF_GNAT+OBfold_anticodonB+K-tRS                                                                                                                                                                                                                                                                                                      lysX                       1177   Mycobacterium avium 104                                        bacteria>actinobacteria                         RecName: Full=Lysylphosphatidylglycerol biosynthesis bifunctional    
289443094    7xTM+MprF_GNAT+OBfold_anticodonB+K-tRS                                                                                                                                                                                                                                                                                                      TBLG_00196                 1172   Mycobacterium tuberculosis T46                                 bacteria>actinobacteria                         lysyl-tRNA synthetase 2 lysX [Mycobacterium tuberculosis T46].       
289750188    7xTM+MprF_GNAT                                                                                                                                                                                                                                                                                                                              TBDG_03601                 794    Mycobacterium tuberculosis T92                                 bacteria>actinobacteria                         lysyl-tRNA synthetase [Mycobacterium tuberculosis T92].              
215445821    7xTM+MprF_GNAT+OBfold_anticodonB+K-tRS                                                                                                                                                                                                                                                                                                      lysS                       1111   Mycobacterium tuberculosis T85                                 bacteria>actinobacteria                         lysyl-tRNA synthetase [Mycobacterium tuberculosis T85].              
215404058    7xTM+MprF_GNAT+OBfold_anticodonB+K-tRS                                                                                                                                                                                                                                                                                                      lysS                       1111   Mycobacterium tuberculosis 02_1987                             bacteria>actinobacteria                         lysyl-tRNA synthetase [Mycobacterium tuberculosis 02_1987].          
215430532    7xTM+MprF_GNAT+OBfold_anticodonB+K-tRS                                                                                                                                                                                                                                                                                                      lysS                       1111   Mycobacterium tuberculosis EAS054                              bacteria>actinobacteria                         lysyl-tRNA synthetase [Mycobacterium tuberculosis EAS054].           
260186588    7xTM+MprF_GNAT+OBfold_anticodonB+K-tRS                                                                                                                                                                                                                                                                                                      lysS                       1111   Mycobacterium tuberculosis CPHL_A                              bacteria>actinobacteria                         lysyl-tRNA synthetase [Mycobacterium tuberculosis CPHL_A].           
41407449     7xTM+MprF_GNAT+OBfold_anticodonB+K-tRS                                                                                                                                                                                                                                                                                                      lysS                       1177   Mycobacterium avium subsp. paratuberculosis K-10               bacteria>actinobacteria                         lysyl-tRNA synthetase [Mycobacterium avium subsp. paratuberculosis   
118468939    7xTM+MprF_GNAT+OBfold_anticodonB+K-tRS                                                                                                                                                                                                                                                                                                      lysS                       1089   Mycobacterium smegmatis str. MC2 155                           bacteria>actinobacteria                         lysyl-tRNA synthetase [Mycobacterium smegmatis str. MC2 155].        
296439733    7xTM+MprF_GNAT+OBfold_anticodonB+K-tRS                                                                                                                                                                                                                                                                                                      lysX                       1106   Mycobacterium smegmatis str. MC2 155                           bacteria>actinobacteria                         RecName: Full=Lysylphosphatidylglycerol biosynthesis bifunctional    
219557559    7xTM+MprF_GNAT+OBfold_anticodonB+K-tRS                                                                                                                                                                                                                                                                                                      lysS                       1111   Mycobacterium tuberculosis T17                                 bacteria>actinobacteria                         lysyl-tRNA synthetase [Mycobacterium tuberculosis T17].              
145224128    7xTM+7xTM+MprF_GNAT+OBfold_anticodonB+K-tRS                                                                                                                                                                                                                                                                                                 lysS                       1101   Mycobacterium gilvum PYR-GCK                                   bacteria>actinobacteria                         lysyl-tRNA synthetase [Mycobacterium gilvum PYR-GCK].                
120404304    7xTM+7xTM+MprF_GNAT+OBfold_anticodonB+K-tRS                                                                                                                                                                                                                                                                                                 lysS                       1100   Mycobacterium vanbaalenii PYR-1                                bacteria>actinobacteria                         lysyl-tRNA synthetase [Mycobacterium vanbaalenii PYR-1].             
296164539    7xTM+MprF_GNAT+OBfold_anticodonB+K-tRS                                                                                                                                                                                                                                                                                                      lysS2                      1087   Mycobacterium parascrofulaceum ATCC BAA-614                    bacteria>actinobacteria                         lysine--tRNA ligase [Mycobacterium parascrofulaceum ATCC BAA-614].   
294630730    7xTM+MprF_GNAT                                                                                                                                                                                                                                                                                                                              SSTG_02731                 602    Streptomyces sp. e14                                           bacteria>actinobacteria                         lysyl-tRNA synthetase [Streptomyces sp. e14].                        
296439735    7xTM+MprF_GNAT+OBfold_anticodonB+K-tRS                                                                                                                                                                                                                                                                                                      lysX                       1133   Mycobacterium leprae Br4923                                    bacteria>actinobacteria                         RecName: Full=Lysylphosphatidylglycerol biosynthesis bifunctional    
126435577    7xTM+UPF0182+MprF_GNAT+OBfold_anticodonB+K-tRS                                                                                                                                                                                                                                                                                              lysS                       1111   Mycobacterium sp. JLS                                          bacteria>actinobacteria                         lysyl-tRNA synthetase [Mycobacterium sp. JLS].                       
254821219    7xTM+MprF_GNAT+OBfold_anticodonB+K-tRS                                                                                                                                                                                                                                                                                                      lysS                       1089   Mycobacterium intracellulare ATCC 13950                        bacteria>actinobacteria                         lysyl-tRNA synthetase [Mycobacterium intracellulare ATCC 13950].     
15827728     7xTM+MprF_GNAT+OBfold_anticodonB+K-tRS                                                                                                                                                                                                                                                                                                      -                          1039   Mycobacterium leprae TN                                        bacteria>actinobacteria                         lysyl-tRNA synthetase [Mycobacterium leprae TN].                     
108799949    7xTM+7xTM+MprF_GNAT+OBfold_anticodonB+K-tRS                                                                                                                                                                                                                                                                                                 lysS                       1112   Mycobacterium sp. MCS                                          bacteria>actinobacteria                         lysyl-tRNA synthetase [Mycobacterium sp. MCS].                       
111020719    7xTM+MprF_GNAT+OBfold_anticodonB+K-tRS                                                                                                                                                                                                                                                                                                      lysS                       1114   Rhodococcus jostii RHA1                                        bacteria>actinobacteria                         lysyl-tRNA synthetase [Rhodococcus jostii RHA1].                     
256771171    7xTM+MprF_GNAT+OBfold_anticodonB+K-tRS                                                                                                                                                                                                                                                                                                      lysS                       1100   Streptomyces sp. C                                             bacteria>actinobacteria                         lysyl-tRNA synthetase [Streptomyces sp. C].                          
226362961    7xTM+MprF_GNAT+OBfold_anticodonB+K-tRS                                                                                                                                                                                                                                                                                                      lysS                       1114   Rhodococcus opacus B4                                          bacteria>actinobacteria                         putative lysyl-tRNA synthetase [Rhodococcus opacus B4].              
226303560    7xTM+MprF_GNAT+OBfold_anticodonB+K-tRS                                                                                                                                                                                                                                                                                                      lysS                       1104   Rhodococcus erythropolis PR4                                   bacteria>actinobacteria                         lysyl-tRNA synthetase [Rhodococcus erythropolis PR4].                
254383488    7xTM+MprF_GNAT+OBfold_anticodonB+K-tRS                                                                                                                                                                                                                                                                                                      SSAG_03141                 1100   Streptomyces sp. Mg1                                           bacteria>actinobacteria                         lysyl-tRNA synthetase [Streptomyces sp. Mg1].                        
299792650    7xTM+MprF_GNAT+OBfold_anticodonB+K-tRS                                                                                                                                                                                                                                                                                                      lysS                       1107   Amycolatopsis mediterranei U32                                 bacteria>actinobacteria                         lysyl-tRNA synthetase, class II [Amycolatopsis mediterranei U32].    
21224696     7xTM+MprF_GNAT                                                                                                                                                                                                                                                                                                                              -                          589    Streptomyces coelicolor A3(2)                                  bacteria>actinobacteria                         integral membrane lysyl-tRNA synthetase [Streptomyces coelicolor     
256818519    7xTM+MprF_GNAT                                                                                                                                                                                                                                                                                                                              SgriT_010100035984         566    Streptomyces griseoflavus Tu4000                               bacteria>actinobacteria                         integral membrane lysyl-tRNA synthetase [Streptomyces griseoflavus   
169629405    7xTM+MprF_GNAT+OBfold_anticodonB+K-tRS                                                                                                                                                                                                                                                                                                      lysS                       1110   Mycobacterium abscessus ATCC 19977                             bacteria>actinobacteria                         lysyl-tRNA synthetase [Mycobacterium abscessus ATCC 19977].          
229492238    7xTM+MprF_GNAT+OBfold_anticodonB+K-tRS                                                                                                                                                                                                                                                                                                      RHOER0001_5574             1118   Rhodococcus erythropolis SK121                                 bacteria>actinobacteria                         putative lysyl-tRNA synthetase [Rhodococcus erythropolis SK121].     
254385657    7xTM+MprF_GNAT                                                                                                                                                                                                                                                                                                                              SSAG_05381                 616    Streptomyces sp. Mg1                                           bacteria>actinobacteria                         integral membrane lysyl-tRNA synthetase [Streptomyces sp. Mg1].      
29833838     7xTM+MprF_GNAT+OBfold_anticodonB+K-tRS                                                                                                                                                                                                                                                                                                      lysS                       1093   Streptomyces avermitilis MA-4680                               bacteria>actinobacteria                         lysyl-tRNA synthetase [Streptomyces avermitilis MA-4680].            
291297601    7xTM+MprF_GNAT+OBfold_anticodonB+K-tRS                                                                                                                                                                                                                                                                                                      Snas_0064                  1100   Stackebrandtia nassauensis DSM 44728                           bacteria>actinobacteria                         lysyl-tRNA synthetase [Stackebrandtia nassauensis DSM 44728].        
294630634    7xTM+MprF_GNAT                                                                                                                                                                                                                                                                                                                              SSTG_02635                 603    Streptomyces sp. e14                                           bacteria>actinobacteria                         lysyl-tRNA synthetase [Streptomyces sp. e14].                        
296439732    7xTM+MprF_GNAT+OBfold_anticodonB+K-tRS                                                                                                                                                                                                                                                                                                      lysX                       1128   Nocardia farcinica                                             bacteria>actinobacteria                         RecName: Full=Lysylphosphatidylglycerol biosynthesis bifunctional    
54022067     7xTM+MprF_GNAT+OBfold_anticodonB+K-tRS                                                                                                                                                                                                                                                                                                      lysS                       1061   Nocardia farcinica IFM 10152                                   bacteria>actinobacteria                         lysyl-tRNA synthetase [Nocardia farcinica IFM 10152].                
256803625    7xTM+MprF_GNAT                                                                                                                                                                                                                                                                                                                              SvirD4_010100022704        571    Streptomyces viridochromogenes DSM 40736                       bacteria>actinobacteria                         integral membrane lysyl-tRNA synthetase [Streptomyces                
256786996    7xTM+MprF_GNAT                                                                                                                                                                                                                                                                                                                              SlivT_010100021118         601    Streptomyces lividans TK24                                     bacteria>actinobacteria                         integral membrane lysyl-tRNA synthetase [Streptomyces lividans       
21221824     7xTM+MprF_GNAT                                                                                                                                                                                                                                                                                                                              -                          601    Streptomyces coelicolor A3(2)                                  bacteria>actinobacteria                         integral membrane lysyl-tRNA synthetase [Streptomyces coelicolor     
297192930    7xTM+MprF_GNAT                                                                                                                                                                                                                                                                                                                              SSDG_04934                 611    Streptomyces pristinaespiralis ATCC 25486                      bacteria>actinobacteria                         integral membrane lysyl-tRNA synthetase [Streptomyces                
284034397    7xTM+MprF_GNAT+OBfold_anticodonB+K-tRS                                                                                                                                                                                                                                                                                                      Kfla_6532                  1117   Kribbella flavida DSM 17836                                    bacteria>actinobacteria                         lysyl-tRNA synthetase [Kribbella flavida DSM 17836].                 
297201407    7xTM+MprF_GNAT                                                                                                                                                                                                                                                                                                                              SSEG_04429                 603    Streptomyces sviceus ATCC 29083                                bacteria>actinobacteria                         integral membrane lysyl-tRNA synthetase [Streptomyces sviceus ATCC   
252125129    13xTM+MprF_GNAT                                                                                                                                                                                                                                                                                                                             CORMA0001_0527             843    Corynebacterium matruchotii ATCC 14266                         bacteria>actinobacteria                         lysine--tRNA ligase [Corynebacterium matruchotii ATCC 14266].        
284990815    7xTM+OBfold_anticodonB+K-tRS+DUF2339+MprF_GNAT                                                                                                                                                                                                                                                                                              Gobs_2319                  1132   Geodermatophilus obscurus DSM 43160                            bacteria>actinobacteria                         lysyl-tRNA synthetase [Geodermatophilus obscurus DSM 43160].         
284043972    7xTM+OBfold_anticodonB+K-tRS+MprF_GNAT                                                                                                                                                                                                                                                                                                      Cwoe_2515                  1118   Conexibacter woesei DSM 14684                                  bacteria>actinobacteria                         lysyl-tRNA synthetase [Conexibacter woesei DSM 14684].               
294787605    13xTM+MprF_GNAT                                                                                                                                                                                                                                                                                                                             HMPREF9017_01504           892    Parascardovia denticolens F0305                                bacteria>actinobacteria                         lysine--tRNA ligase [Parascardovia denticolens F0305].               
296392770    7xTM+MprF_GNAT+OBfold_anticodonB+K-tRS+K-tRSd                                                                                                                                                                                                                                                                                               Srot_0336                  1091   Segniliparus rotundus DSM 44985                                bacteria>actinobacteria                         lysyl-tRNA synthetase [Segniliparus rotundus DSM 44985].             
271964174    7xTM+MprF_GNAT+OBfold_anticodonB+K-tRS                                                                                                                                                                                                                                                                                                      Sros_2661                  1098   Streptosporangium roseum DSM 43021                             bacteria>actinobacteria                         Lysyl-tRNA synthetase (class II)-like protein [Streptosporangium     
269128167    7xTM+MprF_GNAT+OBfold_anticodonB+K-tRS                                                                                                                                                                                                                                                                                                      Tcur_3970                  1113   Thermomonospora curvata DSM 43183                              bacteria>actinobacteria                         lysyl-tRNA synthetase [Thermomonospora curvata DSM 43183].           
38234687     7xTM+MprF_GNAT+OBfold_anticodonB+K-tRS                                                                                                                                                                                                                                                                                                      -                          1049   Corynebacterium diphtheriae NCTC 13129                         bacteria>actinobacteria                         lysyl-tRNA synthetase [Corynebacterium diphtheriae NCTC 13129].      
255326539    7xTM+MprF_GNAT+OBfold_anticodonB+K-tRS                                                                                                                                                                                                                                                                                                      ROTMU0001_1526             1085   Rothia mucilaginosa ATCC 25296                                 bacteria>actinobacteria                         lysine--tRNA ligase [Rothia mucilaginosa ATCC 25296].                
283458954    7xTM+MprF_GNAT+OBfold_anticodonB+K-tRS                                                                                                                                                                                                                                                                                                      RMDY18_19500               1116   Rothia mucilaginosa DY-18                                      bacteria>actinobacteria                         lysyl-tRNA synthetase [Rothia mucilaginosa DY-18].                   
256666356    7xTM+MprF_GNAT+OBfold_anticodonB+K-tRS                                                                                                                                                                                                                                                                                                      lysS                       1103   Streptomyces sp. AA4                                           bacteria>actinobacteria                         lysyl-tRNA synthetase [Streptomyces sp. AA4].                        
256375052    7xTM+MprF_GNAT+OBfold_anticodonB+K-tRS                                                                                                                                                                                                                                                                                                      lysS                       1094   Actinosynnema mirum DSM 43827                                  bacteria>actinobacteria                         lysyl-tRNA synthetase [Actinosynnema mirum DSM 43827].               
50955081     13xTM+MprF_GNAT                                                                                                                                                                                                                                                                                                                             lysX                       796    Leifsonia xyli subsp. xyli str. CTCB07                         bacteria>actinobacteria                         lysyl-tRNA synthetase [Leifsonia xyli subsp. xyli str. CTCB07].      
296934611    7xTM+MprF_GNAT+OBfold_anticodonB+K-tRS                                                                                                                                                                                                                                                                                                      lysS2                      1110   Rothia dentocariosa ATCC 17931                                 bacteria>actinobacteria                         lysine--tRNA ligase [Rothia dentocariosa ATCC 17931].                
297571583    7xTM+MprF_GNAT+OBfold_anticodonB+K-tRS                                                                                                                                                                                                                                                                                                      Arch_1018                  1062   Arcanobacterium haemolyticum DSM 20595                         bacteria>actinobacteria                         lysyl-tRNA synthetase [Arcanobacterium haemolyticum DSM 20595].      
269794500    7xTM+MprF_GNAT+OBfold_anticodonB+K-tRS                                                                                                                                                                                                                                                                                                      Sked_11780                 1147   Sanguibacter keddieii DSM 10542                                bacteria>actinobacteria                         lysyl-tRNA synthetase (class II) [Sanguibacter keddieii DSM 10542].  
219683605    13xTM+MprF_GNAT                                                                                                                                                                                                                                                                                                                             lysX                       922    Bifidobacterium animalis subsp. lactis AD011                   bacteria>actinobacteria                         lysyl-tRNA synthetase [Bifidobacterium animalis subsp. lactis        
291008865    7xTM+MprF_GNAT+OBfold_anticodonB+K-tRS                                                                                                                                                                                                                                                                                                      lysS                       1099   Saccharopolyspora erythraea NRRL 2338                          bacteria>actinobacteria                         lysyl-tRNA synthetase [Saccharopolyspora erythraea NRRL 2338].       
134101270    7xTM+MprF_GNAT+OBfold_anticodonB+K-tRS                                                                                                                                                                                                                                                                                                      lysS                       1098   Saccharopolyspora erythraea NRRL 2338                          bacteria>actinobacteria                         lysyl-tRNA synthetase [Saccharopolyspora erythraea NRRL 2338].       
297625605    7xTM+MprF_GNAT                                                                                                                                                                                                                                                                                                                              lysX                       687    Propionibacterium freudenreichii subsp. shermanii CIRM-BIA1    bacteria>actinobacteria                         Lysyl-tRNA synthetase [Propionibacterium freudenreichii subsp.       
261338083    13xTM+MprF_GNAT                                                                                                                                                                                                                                                                                                                             BIFGAL_03579               930    Bifidobacterium gallicum DSM 20093                             bacteria>actinobacteria                         lysine--tRNA ligase [Bifidobacterium gallicum DSM 20093].            
229489879    7xTM+MprF_GNAT                                                                                                                                                                                                                                                                                                                              RHOER0001_5510             866    Rhodococcus erythropolis SK121                                 bacteria>actinobacteria                         lysyl-tRNA synthetase [Rhodococcus erythropolis SK121].              
282854731    7xTM+MprF_GNAT+OBfold_anticodonB+K-tRS                                                                                                                                                                                                                                                                                                      lysS                       1109   Propionibacterium acnes J139                                   bacteria>actinobacteria                         lysine--tRNA ligase [Propionibacterium acnes J139].                  
295395018    7xTM+MprF_GNAT+OBfold_anticodonB+K-tRS                                                                                                                                                                                                                                                                                                      HMPREF0183_0726            1125   Brevibacterium mcbrellneri ATCC 49030                          bacteria>actinobacteria                         probable lysyl-tRNA synthetase [Brevibacterium mcbrellneri ATCC      
289426405    7xTM+MprF_GNAT+OBfold_anticodonB+K-tRS                                                                                                                                                                                                                                                                                                      lysS                       1081   Propionibacterium acnes SK187                                  bacteria>actinobacteria                         lysine--tRNA ligase [Propionibacterium acnes SK187].                 
289428675    7xTM+MprF_GNAT+OBfold_anticodonB+K-tRS                                                                                                                                                                                                                                                                                                      lysS                       1081   Propionibacterium acnes J165                                   bacteria>actinobacteria                         lysine--tRNA ligase [Propionibacterium acnes J165].                  
295131136    7xTM+MprF_GNAT+OBfold_anticodonB+K-tRS                                                                                                                                                                                                                                                                                                      lysS_2                     1081   Propionibacterium acnes SK137                                  bacteria>actinobacteria                         lysine--tRNA ligase [Propionibacterium acnes SK137].                 
262200161    7xTM+MprF_GNAT+OBfold_anticodonB+K-tRS                                                                                                                                                                                                                                                                                                      Gbro_0127                  1138   Gordonia bronchialis DSM 43247                                 bacteria>actinobacteria                         lysyl-tRNA synthetase [Gordonia bronchialis DSM 43247].              
50843089     7xTM+MprF_GNAT+OBfold_anticodonB+K-tRS                                                                                                                                                                                                                                                                                                      lysS                       1120   Propionibacterium acnes KPA171202                              bacteria>actinobacteria                         lysyl-tRNA synthetase [Propionibacterium acnes KPA171202].           
299791723    7xTM+MprF_GNAT                                                                                                                                                                                                                                                                                                                              lysS                       571    Amycolatopsis mediterranei U32                                 bacteria>actinobacteria                         lysyl-tRNA synthetase [Amycolatopsis mediterranei U32].              
296139451    7xTM+MprF_GNAT+OBfold_anticodonB+K-tRS                                                                                                                                                                                                                                                                                                      Tpau_1737                  1120   Tsukamurella paurometabola DSM 20162                           bacteria>actinobacteria                         lysyl-tRNA synthetase [Tsukamurella paurometabola DSM 20162].        
68536277     7xTM+MprF_GNAT+OBfold_anticodonB+K-tRS                                                                                                                                                                                                                                                                                                      lysS                       1082   Corynebacterium jeikeium K411                                  bacteria>actinobacteria                         lysyl-tRNA synthetase [Corynebacterium jeikeium K411].               
260578976    7xTM+MprF_GNAT+OBfold_anticodonB+K-tRS                                                                                                                                                                                                                                                                                                      lysX                       1050   Corynebacterium jeikeium ATCC 43734                            bacteria>actinobacteria                         lysine--tRNA ligase [Corynebacterium jeikeium ATCC 43734].           
227494365    7xTM+MprF_GNAT+OBfold_anticodonB+K-tRS                                                                                                                                                                                                                                                                                                      lysS                       1084   Actinomyces coleocanis DSM 15436                               bacteria>actinobacteria                         lysyl-tRNA synthetase [Actinomyces coleocanis DSM 15436].            
184201797    7xTM+MprF_GNAT+OBfold_anticodonB+K-tRS                                                                                                                                                                                                                                                                                                      lysS                       1099   Kocuria rhizophila DC2201                                      bacteria>actinobacteria                         lysyl-tRNA synthetase [Kocuria rhizophila DC2201].                   
269219313    7xTM+MprF_GNAT+OBfold_anticodonB+K-tRS                                                                                                                                                                                                                                                                                                      HMPREF0972_01964           1167   Actinomyces sp. oral taxon 848 str. F0332                      bacteria>actinobacteria                         lysine--tRNA ligase [Actinomyces sp. oral taxon 848 str. F0332].     
111018637    7xTM+MprF_GNAT                                                                                                                                                                                                                                                                                                                              RHA1_ro01638               455    Rhodococcus jostii RHA1                                        bacteria>actinobacteria                         lysyl-tRNA synthetase [Rhodococcus jostii RHA1].                     

#;7xTM+MprF_like-GNAT+K-TRSOB
15673099     7xTM+MprF_GNAT+OBfold_anticodonB                                                                                                                                                                                                                                                                                                            ylcG                       921    Lactococcus lactis subsp. lactis Il1403                        bacteria>firmicutes                             hypothetical protein L129283 [Lactococcus lactis subsp. lactis       
125624256    7xTM+MprF_GNAT+OBfold_anticodonB                                                                                                                                                                                                                                                                                                            llmg_1448                  917    Lactococcus lactis subsp. cremoris MG1363                      bacteria>firmicutes                             hypothetical protein llmg_1448 [Lactococcus lactis subsp. cremoris   
281491613    7xTM+MprF_GNAT+OBfold_anticodonB                                                                                                                                                                                                                                                                                                            lysS                       921    Lactococcus lactis subsp. lactis KF147                         bacteria>firmicutes                             lysyl-tRNA synthetase [Lactococcus lactis subsp. lactis KF147].      
116511956    7xTM+MprF_GNAT+OBfold_anticodonB                                                                                                                                                                                                                                                                                                            LACR_1219                  917    Lactococcus lactis subsp. cremoris SK11                        bacteria>firmicutes                             hypothetical protein LACR_1219 [Lactococcus lactis subsp. cremoris   

#;13xTM(13xTM(UPF0104))+MprF_like-GNAT
257896236    13xTM(UPF0104)+MprF_GNAT                                                                                                                                                                                                                                                                                                                    EFVG_01190                 863    Enterococcus faecium Com12                                     bacteria>firmicutes                             lysyl-tRNA synthetase [Enterococcus faecium Com12].                  
257884910    13xTM(UPF0104)+MprF_GNAT                                                                                                                                                                                                                                                                                                                    EFRG_01214                 863    Enterococcus faecium 1,231,501                                 bacteria>firmicutes                             lysyl-tRNA synthetase [Enterococcus faecium 1,231,501].              
294614877    13xTM(UPF0104)+MprF_GNAT                                                                                                                                                                                                                                                                                                                    EfmE1636_0962              866    Enterococcus faecium E1636                                     bacteria>firmicutes                             lysyl-tRNA synthetase [Enterococcus faecium E1636].                  
293556710    13xTM(UPF0104)+MprF_GNAT                                                                                                                                                                                                                                                                                                                    EfmE1039_2015              863    Enterococcus faecium E1039                                     bacteria>firmicutes                             lysyl-tRNA synthetase [Enterococcus faecium E1039].                  
257898874    13xTM(UPF0104)+MprF_GNAT                                                                                                                                                                                                                                                                                                                    EFWG_01222                 863    Enterococcus faecium Com15                                     bacteria>firmicutes                             lysyl-tRNA synthetase [Enterococcus faecium Com15].                  
227522493    13xTM(UPF0104)+MprF_GNAT                                                                                                                                                                                                                                                                                                                    HMPREF0519_0202            855    Lactobacillus hilgardii ATCC 8290                              bacteria>firmicutes                             lysyl-tRNA synthetase (class II) [Lactobacillus hilgardii ATCC       
293570220    13xTM(UPF0104)+MprF_GNAT                                                                                                                                                                                                                                                                                                                    EfmE980_0036               863    Enterococcus faecium E980                                      bacteria>firmicutes                             lysyl-tRNA synthetase [Enterococcus faecium E980].                   
260662711    13xTM(UPF0104)+MprF_GNAT                                                                                                                                                                                                                                                                                                                    HMPREF0513_00722           862    Lactobacillus fermentum 28-3-CHN                               bacteria>firmicutes                             lysyl-tRNA synthetase [Lactobacillus fermentum 28-3-CHN].            
116628000    13xTM(UPF0104)+MprF_GNAT                                                                                                                                                                                                                                                                                                                    STER_1235                  851    Streptococcus thermophilus LMD-9                               bacteria>firmicutes                             lysyl-tRNA synthetase (class II) [Streptococcus thermophilus         
227530165    13xTM(UPF0104)+MprF_GNAT                                                                                                                                                                                                                                                                                                                    HMPREF0549_1344            861    Lactobacillus vaginalis ATCC 49540                             bacteria>firmicutes                             lysyl-tRNA synthetase (class II) [Lactobacillus vaginalis ATCC       
228476865    13xTM(UPF0104)+MprF_GNAT                                                                                                                                                                                                                                                                                                                    STRSA0001_1327             851    Streptococcus salivarius SK126                                 bacteria>firmicutes                             lysyl-tRNA synthetase [Streptococcus salivarius SK126].              
116333241    13xTM(UPF0104)+MprF_GNAT                                                                                                                                                                                                                                                                                                                    LVIS_0592                  870    Lactobacillus brevis ATCC 367                                  bacteria>firmicutes                             lysyl-tRNA synthetase (class II) [Lactobacillus brevis ATCC 367].    
227896457    13xTM(UPF0104)+MprF_GNAT                                                                                                                                                                                                                                                                                                                    HMPREF0531_1770            867    Lactobacillus plantarum subsp. plantarum ATCC 14917            bacteria>firmicutes                             Lysyl-tRNA synthetase (class II) [Lactobacillus plantarum subsp.     
257877969    13xTM(UPF0104)+MprF_GNAT                                                                                                                                                                                                                                                                                                                    EFPG_00055                 863    Enterococcus faecium 1,230,933                                 bacteria>firmicutes                             lysyl-tRNA synthetase [Enterococcus faecium 1,230,933].              
227891215    13xTM(UPF0104)+MprF_GNAT                                                                                                                                                                                                                                                                                                                    HMPREF0545_0714            868    Lactobacillus salivarius ATCC 11741                            bacteria>firmicutes                             Lysyl-tRNA synthetase (class II) [Lactobacillus salivarius ATCC      
227517949    13xTM(UPF0104)+MprF_GNAT                                                                                                                                                                                                                                                                                                                    HMPREF0348_0932            843    Enterococcus faecalis TX0104                                   bacteria>firmicutes                             Lysyl-tRNA synthetase (class II) [Enterococcus faecalis TX0104].     
257865687    13xTM(UPF0104)+MprF_GNAT                                                                                                                                                                                                                                                                                                                    EGAG_00558                 847    Enterococcus casseliflavus EC30                                bacteria>firmicutes                             lysyl-tRNA synthetase [Enterococcus casseliflavus EC30].             
227533722    13xTM(UPF0104)+MprF_GNAT                                                                                                                                                                                                                                                                                                                    HMPREF0530_1008            880    Lactobacillus paracasei subsp. paracasei ATCC 25302            bacteria>firmicutes                             lysyl-tRNA synthetase (class II) [Lactobacillus paracasei subsp.     
257875315    13xTM(UPF0104)+MprF_GNAT                                                                                                                                                                                                                                                                                                                    ECBG_00570                 848    Enterococcus casseliflavus EC20                                bacteria>firmicutes                             lysyl-tRNA synthetase [Enterococcus casseliflavus EC20].             
116495719    13xTM(UPF0104)+MprF_GNAT                                                                                                                                                                                                                                                                                                                    LSEI_2267                  869    Lactobacillus casei ATCC 334                                   bacteria>firmicutes                             lysyl-tRNA synthetase (class II) [Lactobacillus casei ATCC 334].     
239630124    13xTM(UPF0104)+MprF_GNAT                                                                                                                                                                                                                                                                                                                    LBPG_02186                 869    Lactobacillus paracasei subsp. paracasei 8700:2                bacteria>firmicutes                             lysyl-tRNA synthetase [Lactobacillus paracasei subsp. paracasei      
191639207    13xTM(UPF0104)+MprF_GNAT                                                                                                                                                                                                                                                                                                                    LCABL_24490                869    Lactobacillus casei BL23                                       bacteria>firmicutes                             Lysyl-tRNA synthetase (Class II) [Lactobacillus casei BL23].         
229552940    13xTM(UPF0104)+MprF_GNAT                                                                                                                                                                                                                                                                                                                    HMPREF0539_2197            891    Lactobacillus rhamnosus LMS2-1                                 bacteria>firmicutes                             Lysyl-tRNA synthetase (class II) [Lactobacillus rhamnosus LMS2-1].   
258509265    13xTM(UPF0104)+MprF_GNAT                                                                                                                                                                                                                                                                                                                    LGG_02270                  869    Lactobacillus rhamnosus GG                                     bacteria>firmicutes                             Lysyl-tRNA synthetase (Class II) [Lactobacillus rhamnosus GG].       
199599023    13xTM(UPF0104)+MprF_GNAT                                                                                                                                                                                                                                                                                                                    LRH_08533                  869    Lactobacillus rhamnosus HN001                                  bacteria>firmicutes                             Lysyl-tRNA synthetase (class II) [Lactobacillus rhamnosus HN001].    
227873902    13xTM(UPF0104)+MprF_GNAT                                                                                                                                                                                                                                                                                                                    HMPREF6123_2062            862    Oribacterium sinus F0268                                       bacteria>firmicutes                             lysyl-tRNA synthetase (class II) [Oribacterium sinus F0268].         
116617503    13xTM(UPF0104)+MprF_GNAT                                                                                                                                                                                                                                                                                                                    LEUM_0379                  844    Leuconostoc mesenteroides subsp. mesenteroides ATCC 8293       bacteria>firmicutes                             lysyl-tRNA synthetase (class II) [Leuconostoc mesenteroides subsp.   
257868984    13xTM(UPF0104)+MprF_GNAT                                                                                                                                                                                                                                                                                                                    EGBG_00572                 848    Enterococcus gallinarum EG2                                    bacteria>firmicutes                             lysyl-tRNA synthetase [Enterococcus gallinarum EG2].                 
227363859    13xTM(UPF0104)+MprF_GNAT                                                                                                                                                                                                                                                                                                                    HMPREF0535_0802            873    Lactobacillus reuteri MM2-3                                    bacteria>firmicutes                             Lysyl-tRNA synthetase (class II) [Lactobacillus reuteri MM2-3].      
227544026    13xTM(UPF0104)+MprF_GNAT                                                                                                                                                                                                                                                                                                                    HMPREF0534_0612            873    Lactobacillus reuteri CF48-3A                                  bacteria>firmicutes                             Lysyl-tRNA synthetase (class II) [Lactobacillus reuteri CF48-3A].    
217964156    13xTM(UPF0104)+MprF_GNAT                                                                                                                                                                                                                                                                                                                    LMHCC_0869                 865    Listeria monocytogenes HCC23                                   bacteria>firmicutes                             lysyl-tRNA synthetase [Listeria monocytogenes HCC23].                
282883361    13xTM(UPF0104)+MprF_GNAT                                                                                                                                                                                                                                                                                                                    HMPREF0628_0980            842    Peptoniphilus lacrimalis 315-B                                 bacteria>firmicutes                             lysine--tRNA ligase [Peptoniphilus lacrimalis 315-B].                
254873884    13xTM(UPF0104)+MprF_GNAT                                                                                                                                                                                                                                                                                                                    LmonocytogFSL_030100014077 906    Listeria monocytogenes FSL N1-017                              bacteria>firmicutes                             lysyl-tRNA synthetase [Listeria monocytogenes FSL N1-017].           
227528796    13xTM(UPF0104)+MprF_GNAT                                                                                                                                                                                                                                                                                                                    HMPREF0542_2239            857    Lactobacillus ruminis ATCC 25644                               bacteria>firmicutes                             Lysyl-tRNA synthetase (class II) [Lactobacillus ruminis ATCC         
258540450    13xTM(UPF0104)+MprF_GNAT                                                                                                                                                                                                                                                                                                                    LC705_02259                660    Lactobacillus rhamnosus Lc 705                                 bacteria>firmicutes                             lysyl-tRNA synthetase (Class II) [Lactobacillus rhamnosus Lc 705].   
227552705    13xTM(UPF0104)+MprF_GNAT                                                                                                                                                                                                                                                                                                                    HMPREF0352_2647            850    Enterococcus faecium TX1330                                    bacteria>firmicutes                             Lysyl-tRNA synthetase (class II) [Enterococcus faecium TX1330].      
257887743    13xTM(UPF0104)+MprF_GNAT                                                                                                                                                                                                                                                                                                                    EFSG_01295                 863    Enterococcus faecium 1,141,733                                 bacteria>firmicutes                             lysyl-tRNA synthetase [Enterococcus faecium 1,141,733].              
293572671    13xTM(UPF0104)+MprF_GNAT                                                                                                                                                                                                                                                                                                                    EfmE980_2391               857    Enterococcus faecium E980                                      bacteria>firmicutes                             lysyl-tRNA synthetase [Enterococcus faecium E980].                   
293553656    13xTM(UPF0104)+MprF_GNAT                                                                                                                                                                                                                                                                                                                    EfmE1039_1022              857    Enterococcus faecium E1039                                     bacteria>firmicutes                             lysyl-tRNA synthetase [Enterococcus faecium E1039].                  
255024373    MprF_GNAT                                                                                                                                                                                                                                                                                                                                   LmonocyFSL_020200014721    202    Listeria monocytogenes FSL J1-208                              bacteria>firmicutes                             lysyl-tRNA synthetase [Listeria monocytogenes FSL J1-208].           
229550671    13xTM(UPF0104)+MprF_GNAT                                                                                                                                                                                                                                                                                                                    HMPREF0345_2883            863    Enterococcus faecalis ATCC 29200                               bacteria>firmicutes                             Lysyl-tRNA synthetase (Class II) [Enterococcus faecalis ATCC         
238856223    13xTM(UPF0104)+MprF_GNAT                                                                                                                                                                                                                                                                                                                    ENTFA0001_0719             863    Enterococcus faecalis TUSoD Ef11                               bacteria>firmicutes                             lysine--tRNA ligase [Enterococcus faecalis TUSoD Ef11].              
227821502    13xTM(UPF0104)+MprF_GNAT                                                                                                                                                                                                                                                                                                                    NGR_c09290                 870    Rhizobium sp. NGR234                                           bacteria>proteobacteria>alphaproteobacteria     lysyl-tRNA synthetase 2 [Rhizobium sp. NGR234].                      

#Fungal homologs; D-TRS+Fem/MurM
119497261    D-tRS+MprF_GNAT                                                                                                                                                                                                                                                                                                                             NFIA_022050                948    Neosartorya fischeri NRRL 181                                  eukaryota>fungi>ascomycota                      aspartyl-tRNA synthetase, cytoplasmic [Neosartorya fischeri NRRL     
291188744    D-tRS+MprF_GNAT                                                                                                                                                                                                                                                                                                                             TRV_01140                  947    Trichophyton verrucosum HKI 0517                               eukaryota>fungi>ascomycota                      aspartate-tRNA ligase, putative [Trichophyton verrucosum HKI 0517].  
291175868    D-tRS+MprF_GNAT                                                                                                                                                                                                                                                                                                                             ARB_01269                  947    Arthroderma benhamiae CBS 112371                               eukaryota>fungi>ascomycota                      aspartate-tRNA ligase, putative [Arthroderma benhamiae CBS 112371].  
145229355    D-tRS+MprF_GNAT                                                                                                                                                                                                                                                                                                                             An01g05630                 952    Aspergillus niger CBS 513.88                                   eukaryota>fungi>ascomycota                      hypothetical protein An01g05630 [Aspergillus niger].                 
67516065     D-tRS+MprF_GNAT                                                                                                                                                                                                                                                                                                                             AN0314.2                   956    Aspergillus nidulans FGSC A4                                   eukaryota>fungi>ascomycota                      hypothetical protein AN0314.2 [Aspergillus nidulans FGSC A4].        
296811126    D-tRS+MprF_GNAT                                                                                                                                                                                                                                                                                                                             MCYG_05770                 946    Arthroderma otae CBS 113480                                    eukaryota>fungi>ascomycota                      aspartyl-tRNA synthetase [Arthroderma otae CBS 113480].              
258566145    D-tRS+MprF_GNAT                                                                                                                                                                                                                                                                                                                             UREG_06784                 938    Uncinocarpus reesii 1704                                       eukaryota>fungi>ascomycota                      hypothetical protein UREG_06784 [Uncinocarpus reesii 1704].          
169766764    D-tRS+MprF_GNAT                                                                                                                                                                                                                                                                                                                             AO090005000838             954    Aspergillus oryzae RIB40                                       eukaryota>fungi>ascomycota                      hypothetical protein [Aspergillus oryzae RIB40].                     
255950584    D-tRS+MprF_GNAT                                                                                                                                                                                                                                                                                                                             Pc22g21630                 967    Penicillium chrysogenum Wisconsin 54-1255                      eukaryota>fungi>ascomycota                      Pc22g21630 [Penicillium chrysogenum Wisconsin 54-1255].              
134114419    D-tRS+MprF_GNAT                                                                                                                                                                                                                                                                                                                             CNBG4380                   884    Cryptococcus neoformans var. neoformans B-3501A                eukaryota>fungi>basidiomycota                   hypothetical protein CNBG4380 [Cryptococcus neoformans var.          
164663385    D-tRS+MprF_GNAT                                                                                                                                                                                                                                                                                                                             MGL_0589                   995    Malassezia globosa CBS 7966                                    eukaryota>fungi>basidiomycota                   hypothetical protein MGL_0589 [Malassezia globosa CBS 7966].         
70990316     D-tRS+MprF_GNAT                                                                                                                                                                                                                                                                                                                             AFUA_1G02570               947    Aspergillus fumigatus Af293                                    eukaryota>fungi>ascomycota                      aspartyl-tRNA synthetase, cytoplasmic [Aspergillus fumigatus         
256720940    D-tRS+MprF_GNAT                                                                                                                                                                                                                                                                                                                             NECHADRAFT_96752           812    Nectria haematococca mpVI 77-13-4                              eukaryota>fungi>ascomycota                      hypothetical protein NECHADRAFT_96752 [Nectria haematococca mpVI     
295663653    D-tRS+MprF_GNAT                                                                                                                                                                                                                                                                                                                             PAAG_05664                 968    Paracoccidioides brasiliensis Pb01                             eukaryota>fungi>ascomycota                      aspartyl-tRNA synthetase [Paracoccidioides brasiliensis Pb01].       
212539668    D-tRS+MprF_GNAT                                                                                                                                                                                                                                                                                                                             PMAA_051900                951    Penicillium marneffei ATCC 18224                               eukaryota>fungi>ascomycota                      aspartyl-tRNA synthetase, cytoplasmic [Penicillium marneffei ATCC    
121703221    D-tRS+MprF_GNAT                                                                                                                                                                                                                                                                                                                             ACLA_031840                958    Aspergillus clavatus NRRL 1                                    eukaryota>fungi>ascomycota                      aspartyl-tRNA synthetase, cytoplasmic [Aspergillus clavatus NRRL     
226287399    D-tRS+MprF_GNAT                                                                                                                                                                                                                                                                                                                             PADG_07732                 968    Paracoccidioides brasiliensis Pb18                             eukaryota>fungi>ascomycota                      aspartyl-tRNA synthetase [Paracoccidioides brasiliensis Pb18].       
225677864    D-tRS+MprF_GNAT                                                                                                                                                                                                                                                                                                                             PABG_06235                 968    Paracoccidioides brasiliensis Pb03                             eukaryota>fungi>ascomycota                      aspartyl-tRNA synthetase [Paracoccidioides brasiliensis Pb03].       
154305343    D-tRS+MprF_GNAT                                                                                                                                                                                                                                                                                                                             BC1G_08966                 524    Botryotinia fuckeliana B05.10                                  eukaryota>fungi>ascomycota                      hypothetical protein BC1G_08966 [Botryotinia fuckeliana B05.10].     
115396750    D-tRS+MprF_GNAT                                                                                                                                                                                                                                                                                                                             ATEG_04836                 909    Aspergillus terreus NIH2624                                    eukaryota>fungi>ascomycota                      aspartyl-tRNA synthetase [Aspergillus terreus NIH2624].              
58269164     D-tRS+MprF_GNAT                                                                                                                                                                                                                                                                                                                             CNG00400                   884    Cryptococcus neoformans var. neoformans JEC21                  eukaryota>fungi>basidiomycota                   aspartate--tRNA ligase [Cryptococcus neoformans var. neoformans      
156040994    D-tRS+MprF_GNAT                                                                                                                                                                                                                                                                                                                             SS1G_11475                 968    Sclerotinia sclerotiorum 1980 UF-70                            eukaryota>fungi>ascomycota                      hypothetical protein SS1G_11475 [Sclerotinia sclerotiorum 1980].     
154280104    D-tRS+MprF_GNAT                                                                                                                                                                                                                                                                                                                             HCAG_04705                 973    Ajellomyces capsulatus NAm1                                    eukaryota>fungi>ascomycota                      hypothetical protein HCAG_04705 [Ajellomyces capsulatus NAm1].       
242803977    D-tRS+MprF_GNAT                                                                                                                                                                                                                                                                                                                             TSTA_021090                946    Talaromyces stipitatus ATCC 10500                              eukaryota>fungi>ascomycota                      aspartyl-tRNA synthetase, cytoplasmic [Talaromyces stipitatus ATCC   
240279712    D-tRS+MprF_GNAT                                                                                                                                                                                                                                                                                                                             HCDG_03115                 949    Ajellomyces capsulatus H143                                    eukaryota>fungi>ascomycota                      aspartyl-tRNA synthetase [Ajellomyces capsulatus H143].              
225562887    D-tRS+MprF_GNAT                                                                                                                                                                                                                                                                                                                             HCBG_00621                 971    Ajellomyces capsulatus G186AR                                  eukaryota>fungi>ascomycota                      aspartyl-tRNA synthetase [Ajellomyces capsulatus G186AR].            
116203221    D-tRS+MprF_GNAT                                                                                                                                                                                                                                                                                                                             CHGG_09495                 916    Chaetomium globosum CBS 148.51                                 eukaryota>fungi>ascomycota                      hypothetical protein CHGG_09495 [Chaetomium globosum CBS 148.51].    
169602527    OBfold_anticodonB+D-tRS                                                                                                                                                                                                                                                                                                                     SNOG_04267                 857    Phaeosphaeria nodorum SN15                                     eukaryota>fungi>ascomycota                      hypothetical protein SNOG_04267 [Phaeosphaeria nodorum SN15].        
239609470    OBfold_anticodonB+D-tRS+MprF_GNAT                                                                                                                                                                                                                                                                                                           BDCG_01577                 941    Ajellomyces dermatitidis ER-3                                  eukaryota>fungi>ascomycota                      aspartyl-tRNA synthetase [Ajellomyces dermatitidis ER-3].            
189203561    OBfold_anticodonB+D-tRS+MprF_GNAT                                                                                                                                                                                                                                                                                                           PTRG_07784                 1041   Pyrenophora tritici-repentis Pt-1C-BFP                         eukaryota>fungi>ascomycota                      aspartyl-tRNA synthetase [Pyrenophora tritici-repentis Pt-1C-BFP].   
171682808    OBfold_anticodonB+D-tRS+MprF_GNAT                                                                                                                                                                                                                                                                                                           PODANSg3375                914    Podospora anserina S mat+                                      eukaryota>fungi>ascomycota                      hypothetical protein [Podospora anserina S mat+].                    
85096905     OBfold_anticodonB+D-tRS+MprF_GNAT                                                                                                                                                                                                                                                                                                           NCU07082                   915    Neurospora crassa OR74A                                        eukaryota>fungi>ascomycota                      hypothetical protein NCU07082 [Neurospora crassa OR74A].             
46110188     OBfold_anticodonB+D-tRS+MprF_GNAT                                                                                                                                                                                                                                                                                                           FG01976.1                  1033   Gibberella zeae PH-1                                           eukaryota>fungi>ascomycota                      hypothetical protein FG01976.1 [Gibberella zeae PH-1].               
119189367    OBfold_anticodonB+D-tRS+MprF_GNAT                                                                                                                                                                                                                                                                                                           CIMG_04731                 937    Coccidioides immitis RS                                        eukaryota>fungi>ascomycota                      hypothetical protein CIMG_04731 [Coccidioides immitis RS].           
145602945    OBfold_anticodonB+D-tRS+MprF_GNAT                                                                                                                                                                                                                                                                                                           MGG_13783                  932    Magnaporthe oryzae 70-15                                       eukaryota>fungi>ascomycota                      hypothetical protein MGG_13783 [Magnaporthe oryzae 70-15].           
261354597    OBfold_anticodonB+D-tRS+MprF_GNAT                                                                                                                                                                                                                                                                                                           VDBG_03134                 730    Verticillium albo-atrum VaMs.102                               eukaryota>fungi>ascomycota                      aspartyl-tRNA synthetase [Verticillium albo-atrum VaMs.102].         
240111265    OBfold_anticodonB+D-tRS+MprF_GNAT                                                                                                                                                                                                                                                                                                           CPC735_071000              937    Coccidioides posadasii C735 delta SOWgp                        eukaryota>fungi>ascomycota                      aspartyl-tRNA synthetase, putative [Coccidioides posadasii C735      
71006800     OBfold_anticodonB+D-tRS+MprF_GNAT                                                                                                                                                                                                                                                                                                           UM01909.1                  1135   Ustilago maydis 521                                            eukaryota>fungi>basidiomycota                   hypothetical protein UM01909.1 [Ustilago maydis 521].                
289617341    OBfold_anticodonB+D-tRS+MprF_GNAT                                                                                                                                                                                                                                                                                                           SMAC_03897                 915    Sordaria macrospora                                            eukaryota>fungi>ascomycota                      unnamed protein product [Sordaria macrospora].                       
261196496    OBfold_anticodonB+D-tRS+MprF_GNAT                                                                                                                                                                                                                                                                                                           BDBG_04515                 941    Ajellomyces dermatitidis SLH14081                              eukaryota>fungi>ascomycota                      aspartyl-tRNA synthetase [Ajellomyces dermatitidis SLH14081].
```


---


9. List of species abbreviations used in the MtRS phylogeny
  

|  |  |
| --- | --- |
| Aae | *Aquifex aeolicus* |
| Abut | *Arcobacter butzleri* |
| Acit | *Acidovorax citrulli* |
| Adeh | *Anaeromyxobacter dehalogenans* |
| Aful | *Archaeoglobus fulgidus* |
| Amir | *Actinosynnema mirum* |
| Asal | *Aliivibrio salmonicida* |
| Asp. | *Anaeromyxobacter sp.* |
| Aper | *Aeropyrum pernix* |
| Atha | *Arabidopsis thaliana* |
| Avar | *Anabaena variabilis* |
| BEll | *bacterium Ellin514* |
| Babo | *Brucella abortus* |
| Bbur | *Borrelia burgdorferi* |
| Bcet | *Brucella ceti* |
| Bhyo | *Brachyspira hyodysenteriae* |
| Blon | *Bifidobacterium longum* |
| Bsp. | *Brucella sp.* |
| Bsub | *Bacillus subtilis* |
| Bsui | *Brucella suis* |
| Bthe | *Bacteroides thetaiotaomicron* |
| Bxen | *Burkholderia xenovorans* |
| Caci | *Catenulispora acidiphila* |
| Cgle | *Chryseobacterium gleum* |
| CKor | *Candidatus Korarchaeum* |
| CPro | *Candidatus Protochlamydia* |
| CSol | *Candidatus Solibacter* |
| Caci | *Catenulispora acidiphila* |
| Caur | *Chloroflexus aurantiacus* |
| Ccav | *Chlamydophila caviae* |
| Ccre | *Caulobacter crescentus* |
| Cper | *Clostridium perfringens* |
| Cpha | *Chlorobium phaeovibrioides* |
| Cpin | *Chitinophaga pinensis* |
| Ctep | *Chlorobium tepidum* |
| Ctra | *Chlamydia trachomatis* |
| Cvio | *Chromobacterium violaceum* |
| Dbac | *Desulfomicrobium baculatum* |
| Ddad | *Dickeya dadantii* |
| Ddis | *Dictyostelium discoideum* |
| Dkam | *Desulfurococcus kamchatkensis* |
| Drad | *Deinococcus radiodurans* |
| Dthe | *Dictyoglomus thermophilum* |
| Ecol | *Escherichia coli* |
| Fnuc | *Fusobacterium nucleatum* |
| Fsp. | *Frankia sp.* |
| Gaur | *Gemmatimonas aurantiaca* |
| Gsul | *Geobacter sulfurreducens* |
| Gvio | *Gloeobacter violaceus* |
| Haur | *Herpetosiphon aurantiacus* |
| Hbut | *Hyperthermus butylicus* |
| Hinf | *Haemophilus influenzae* |
| Hpyl | *Helicobacter pylori* |
| Hsap | *Homo sapiens* |
| Hwal | *Haloquadratum walsbyi* |
| Ihos | *Ignicoccus hospitalis* |
| Lbuc | *Leptotrichia buccalis* |
| Linn | *Listeria innocua* |
| Lint | *Leptospira interrogans* |
| Lpla | *Lactobacillus plantarum* |
| Maer | *Microcystis aeruginosa* |
| Mgen | *Mycoplasma genitalium* |
| Minf | *Methylacidiphilum infernorum* |
| Mjan | *Methanocaldococcus jannaschii* |
| Mlep | *Mycobacterium leprae* |
| Mlot | *Mesorhizobium loti* |
| Mpul | *Mycoplasma pulmonis* |
| Mxan | *Myxococcus xanthus* |
| Nequ | *Nanoarchaeum equitans* |
| Nmar | *Nitrosopumilus maritimus* |
| Pacn | *Propionibacterium acnes* |
| Paer | *Pseudomonas aeruginosa* |
| Pcar | *Pelobacter carbinolicus* |
| Pdis | *Parabacteroides distasonis* |
| Pent | *Pseudomonas entomophila* |
| Pfur | *Pyrococcus furiosus* |
| Pmar | *Persephonella marina* |
| Pmar | *Prochlorococcus marinus* |
| Pmar | *Persephonella marina* |
| Pmob | *Petrotoga mobilis* |
| Ptor | *Picrophilus torridus* |
| Rbal | *Rhodopirellula baltica* |
| Rcas | *Roseiflexus castenholzii* |
| Rsol | *Ralstonia solanacearum* |
| Sare | *Salinispora arenicola* |
| Scel | *Sorangium cellulosum* |
| Scoe | *Streptomyces coelicolor* |
| Smel | *Sinorhizobium meliloti* |
| Smut | *Streptococcus mutans* |
| Spom | *Schizosaccharomyces pombe* |
| Srub | *Salinibacter ruber* |
| Ssol | *Sulfolobus solfataricus* |
| Taci | *Thermoplasma acidophilum* |
| Telo | *Thermosynechococcus elongatus* |
| Tmar | *Thermotoga maritima* |
| Tpal | *Treponema pallidum* |
| Tthe | *Thermus thermophilus* |
| Tyel | *Thermodesulfovibrio yellowstonii* |
| Wsuc | *Wolinella succinogenes* |

---
